# *In vivo* continuous evolution of genes and pathways in yeast

**DOI:** 10.1038/ncomms13051

**Published:** 2016-10-17

**Authors:** Nathan Crook, Joseph Abatemarco, Jie Sun, James M. Wagner, Alexander Schmitz, Hal S. Alper

**Affiliations:** 1 Department of Chemical Engineering, The University of Texas at Austin, 200 East Dean Keeton Street, Stop C0400, Austin, Texas 78712, USA; 2 Institute for Cellular and Molecular Biology, The University of Texas at Austin, 2500 Speedway Avenue, Austin, Texas 78712, USA

## Abstract

Directed evolution remains a powerful, highly generalizable approach for improving the performance of biological systems. However, implementations in eukaryotes rely either on *in vitro* diversity generation or limited mutational capacities. Here we synthetically optimize the retrotransposon Ty1 to enable *in vivo* generation of mutant libraries up to 1.6 × 10^7^ l^−1^ per round, which is the highest of any *in vivo* mutational generation approach in yeast. We demonstrate this approach by using *in vivo*-generated libraries to evolve single enzymes, global transcriptional regulators and multi-gene pathways. When coupled to growth selection, this approach enables *in vivo* continuous evolution (ICE) of genes and pathways. Through a head-to-head comparison, we find that ICE libraries yield higher-performing variants faster than error-prone PCR-derived libraries. Finally, we demonstrate transferability of ICE to divergent yeasts, including *Kluyveromyces lactis* and alternative *S. cerevisiae* strains. Collectively, this work establishes a generic platform for rapid eukaryotic-directed evolution across an array of target cargo.

## Abstract (web summary)

Directed evolution is a powerful technique for generating improved biological systems through repeated rounds of mutagenesis and selection. Here the authors engineer the yeast retrotransposon Ty1 to enable the creation of large mutant libraries *in vivo* and use this system to generate improved variants of single enzymes and multigene pathways.

## Graphical abstract

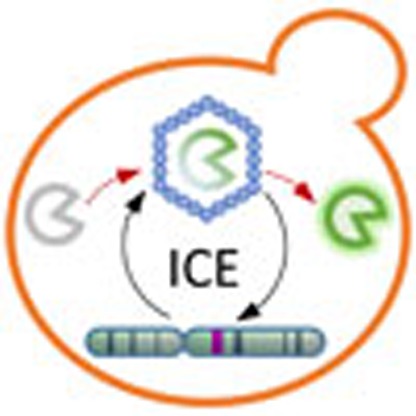

Directed evolution^1^,^2^ serves as a critical bridge between sub-optimal and optimal biological components, even in light of rational design approaches^3^,^4^,^5^. This approach has generated solutions to engineering problems^6^,^7^, established novel functions^8^,^9^ and provided insights into evolution^10^,^11^. As opposed to adaptive evolution, directed evolution aims to identify beneficial mutations within a gene or pathway of interest. Unfortunately, traditional *in vitro* mutagenesis is encumbered by long and costly design–build–test cycles, restrictive requirements for hands-on manipulation of nucleic acids and intrinsic limitations of host transformation efficiency. These limitations become especially poignant when attempting to optimize larger genetic systems (for example, entire pathways including regulatory DNA), especially in more industrially and medically relevant eukaryotic systems. Indeed, the throughput of novel microfluidics-based screening technologies currently outpaces throughput for generation of genetic diversity in these systems^12^. Next-generation evolution techniques aim to accelerate the discovery of improved variants through continuous rounds of mutagenesis/selection on specific DNA cargo with reduced costs using *in vivo* diversity generation. It has been demonstrated that mutational throughput can be increased in *Escherichia coli* in an *in vivo* continuous process using phage, enabling the rapid evolution of parts^13^,^14^,^15^,^16^. However, this approach is best suited for phenotypes linkable to phage growth (for example, DNA-binding proteins) and cannot be applied to eukaryotes. Genome-editing technologies (such as MAGE^17^ and CRISPR-Cas9 (refs 18, 19)) have enabled discovery of sequence–function relationships across a wide range of species but remain, at least at present, a method for introducing finite, defined mutations across several base pairs (ideal when important structural features are known) and are not well-suited for kilobase-scale-directed evolution applications (which are necessary when structural features are largely unknown). In yeast, recent proof-of-concept demonstrations of continuous evolution (1) have suffered from low mutagenic rates and the necessity for mutant expression from weak promoters^20^, or (2) require *in vitro* library generation^21^. Thus, none of these new methods are well suited for the deep mesoscale optimization (that is, generation of all single-nucleotide substitutions to multi-kilobase pathways and gene networks) necessary for evolution of complex multi-part systems or continuous evolution in eukaryotes.

To fill this gap, we establish a scalable, *in vivo* mutagenesis system in yeast by engineering its native retroelement Ty1 (Fig. 1). By tuning the expression of key regulators of Ty1 transposition, we increase library size achievable using this system and confirm its ability to impart a useful error rate to an encoded cargo gene. Next, we apply this system to the directed evolution of a variety of synthetic parts, including single enzymes, regulatory factors and multi-enzyme pathways, realizing substantial and significant improvements to performance in each case. We further demonstrate that ICE enables the recovery of superior mutants more quickly than error-prone PCR. Finally, we show that ICE enables *in vivo* mutant generation across divergent strains of yeast, indicating its applicability towards a wide range of eukaryotic systems.

## Results

### Implementation of ICE

**Figure 1:**
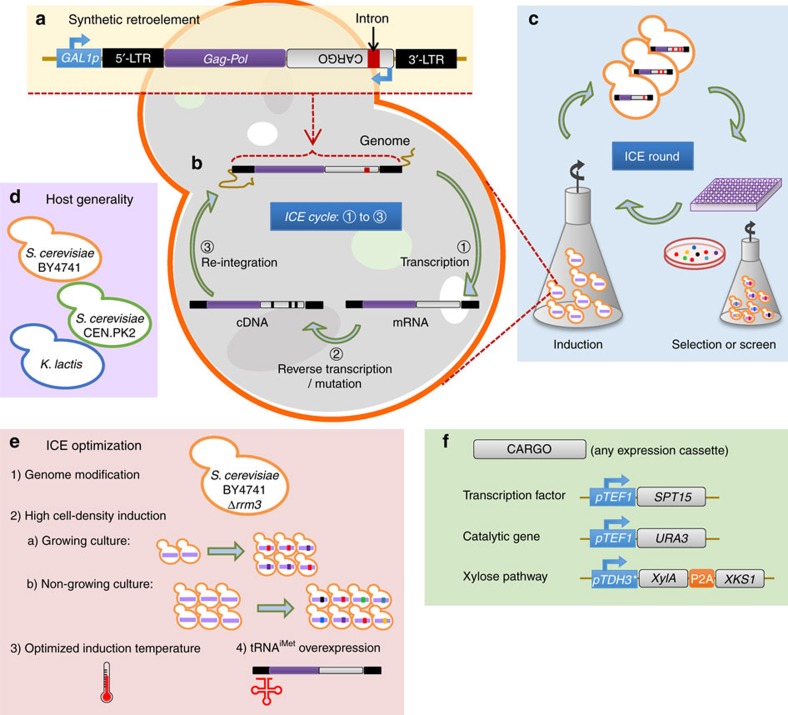
Rationale and schematic for ICE in yeast. In the operational scheme of ICE: (**a**) genetic cargo of interest is cloned into the genome of an inducible Ty1 retrotransposon; (**b**) on induction of retroelement transcription, the encoded reverse transcriptase is expressed, converts the Ty1 genome (including the cargo) into cDNA in an error-prone manner and then this cassette is re-integrated into a stable genomic locus. This process is defined as one cycle; (**c**) the procedure of inducing mutagenesis to a bulk culture and selecting for improved variants is analogously defined as one round. In this work, we (**d**) apply this approach to several divergent strains and species of yeast, (**e**) iteratively improve the efficiency of Ty1 retrotransposition through deletion of *rrm3*, reducing temperature, increasing cell density and increasing expression of limiting cellular components, and (**f**) apply this improved system to the evolution of transcriptional activators, single enzymes and multi-enzyme pathways.

To establish this method, we turned to the native yeast long terminal repeat (LTR) retrotransposon Ty1. The replication cycle of Ty1 proceeds via an RNA intermediate that is converted into complementary DNA through an encoded reverse transcriptase^22^. Previous studies have demonstrated the potential for heterologous gene expression from Ty1 when inserted between *Ty1RT* and the 3′-LTR^23^,^24^. Thus, we reasoned that the error-prone nature of Ty1 replication^25^,^26^ coupled with the capacity for continuous retrotransposon cycling could enable a unique mechanism for *in vivo*-directed mutagenesis of synthetic DNA (denoted here as ‘cargo') in eukaryotes a manner that is scalable with cell count (Fig. 1a). In such a scheme, we define one cycle of *in vivo* mutagenesis as the per-cell process of Ty1-cargo transcription, reverse transcription and re-integration to a stable genetic context (Fig. 1b). As yeast cell densities can routinely exceed 10^10^ l^−1^ (and even 10^12^ l^−1^ in controlled fermentations), library size can easily exceed that of current *in vitro* techniques even with low mutation or transposition rates. A complete round of *in vivo* continuous evolution (ICE), in analogy to traditional directed evolution, is achieved at the culture level by allowing multiple cycles to occur through simple cell outgrowth, screening the resulting *in vivo* library and isolating the best variant. As such, we hypothesized that this approach would enable high-throughput, hands-off, scalable mutagenesis of desired parts and pathways (Fig. 1c). For some applications, rounds may occur continuously and growth-associated phenotypes can be selected in tandem with mutagenesis, thus enabling ICE. In other applications, independent rounds may be desirable to segregate dominant mutations from background genetic drift. We demonstrate both modes of operation in this work.

**Figure 2:**
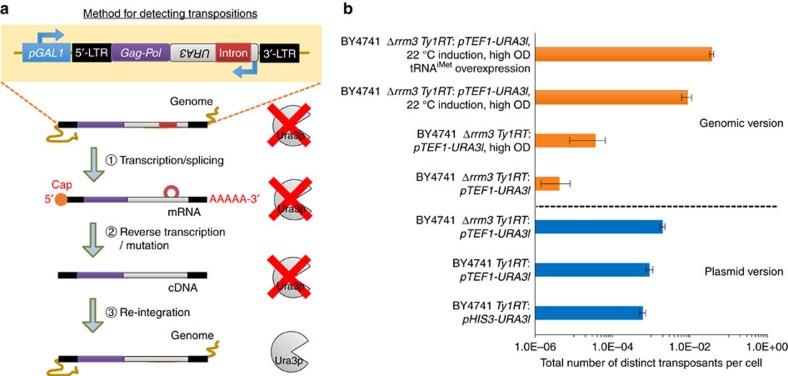
Iterative improvement of synthetic Ty1 transposition rate and scheme for detection of retrotransposition. (**a**) *URA3* is inserted into the retroelement in the reverse orientation relative to transcription from the *pGAL1* promoter. The presence of an intron in the same transcriptional direction as *pGAL1* prevents mRNA originating from the *URA3* promoter from being correctly spliced and initiating Ura3p synthesis. On transcription from *pGAL1*, the intron is spliced. This mRNA cannot give rise to Ura3p due to *URA3* being present in the reverse orientation on this transcript. However, once mRNA is converted into cDNA, a functional *URA3* expression cassette is formed and integration of this cDNA into the genome ensures a heritable *URA3*^*+*^ phenotype. (**b**) Strain background, induction conditions and expression of critical Ty1 components were modified to improve transposition rates of the synthetic retroelement. Error bars for the plasmid version represent 95% confidence intervals obtained via fluctuation analysis of biological triplicates and error bars for the genomic version represent the s.d. of biological triplicates.

To implement and optimize this approach, we adapted a previously described galactose-inducible Ty1 retrotransposon to include a prototrophic marker containing an intron (*URA3I*) between *Ty1RT* and the 3′-LTR in the reverse orientation relative to Ty1 transcription^24^,^27^ (Fig. 2a). This system enables rigorous characterization and optimization of retroelement performance, as transcription, splicing, reverse transcription and re-integration are all necessary to confer uracil prototrophy. As our first implementation and proof-of-concept, *Saccharomyces cerevisiae* BY4741 containing this synthetic, inducible Ty1 on a plasmid was exposed to galactose at low cell density to induce retrotransposition and plated on selective media to measure transposition rate via gained uracil prototrophy after 3 days of growth (see Methods). This phenotype was seen with a frequency of 6.1 × 10^−4^ per cell (Fig. 2b) and not observed when this strain was grown in glucose (which represses *pGAL1*). After demonstrating basal functionality of the plasmid-based synthetic retroelement through induction at low cell density, we wished to develop strategies for increasing transposition rate of Ty1. To this end, we investigated cargo expression level, gene knockouts, cell density, induction temperature and initiator methionine transfer RNA expression level as potential drivers of increased transposition rate. Taken together, this series of iterative design cycles (Fig. 1e) increased the transposition rate (and thus potential library size per round) by over 50-fold to 3.7 × 10^−2^ per cell in simple shake flasks (Fig. 2b).

### Tuning cargo expression increases transposition rate

**Figure 3:**
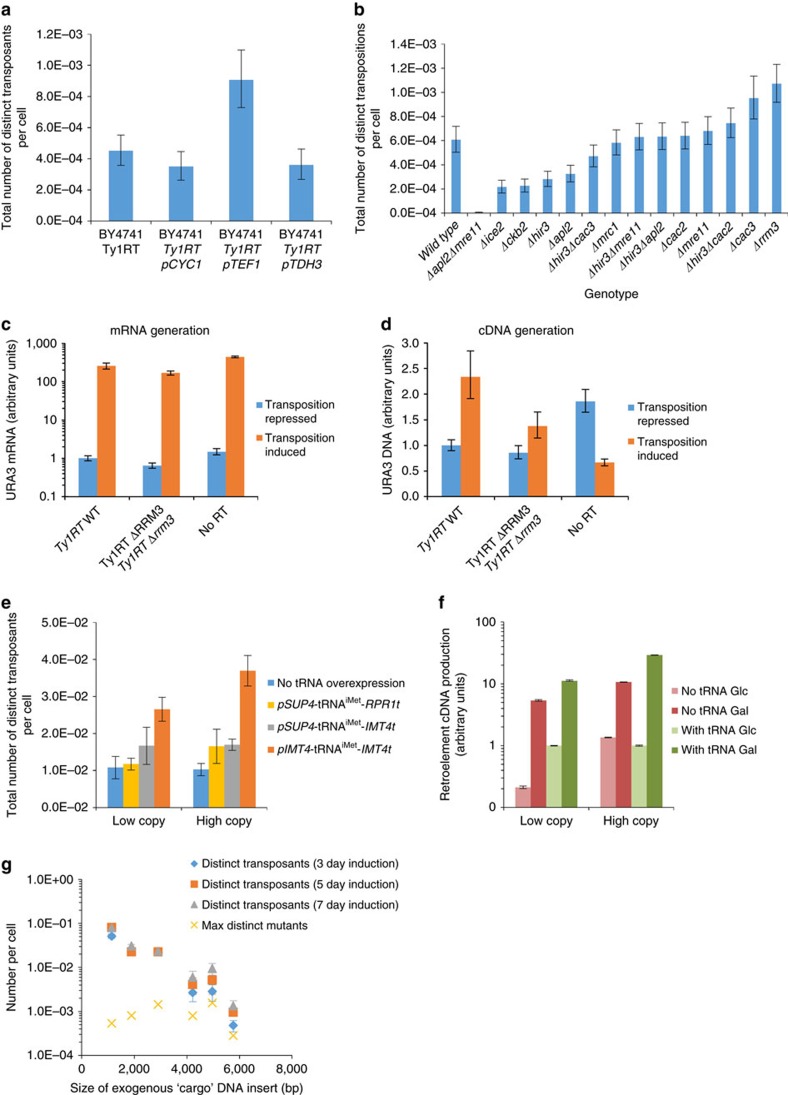
Improvement of Ty1 transposition. (**a**) Substitution of alternative promoters in retroelement. ‘Distinct transposants' refers to the number of unique cells in which Ty1 underwent a full retrotransposition cycle at least once. This uniqueness explicitly excludes daughter cells arising from the original transposed variant. (**b**) Transposition rates for BY4741 knockout strains. Transcript (**c**) and cDNA levels (**d**) of engineered Ty1 retroelements. (**e**) Transposition rate of strains overexpressing the initiator methionine tRNA *IMT4.* (**f**) tRNA^iMet^ overexpression improves cDNA synthesis. Low-copy and high-copy data were collected on different days and hence are normalized to their respective ‘with tRNA Glc' values. (**g**) Transposition rate and mutation rate conferred by retroelements containing cargo of various sizes. ‘Max distinct mutants' refers to the maximum number of mutants attainable in a cargo of a particular length, given a 0.15 kb^−1^ mutation rate and the maximum number of distinct transposants attainable for a particular cargo size (maximum is calculated over 3, 5 and 7-day induction times). Strains containing the appropriate retroelement were exposed to galactose at high OD for (**g**) and low OD for **a** and **b** for 3 days and then plated on uracil-deficient media to count transposants. For **c** and **d**, cells were exposed to the appropriate carbon source at high OD for 3 days. Total DNA and RNA was extracted after induction and nucleic acid levels were quantified using quantitative reverse transcriptase–PCR. For **e** and **f**, strains containing a genomically integrated retroelement were exposed to galactose at 22 °C at high OD for 3 days. Error bars in **a** and **b** represent 95% confidence intervals from biological triplicates. Error bars in **e** and **g** represent the s.d. of biological triplicates. For **c**,**d** and **f** error bars represent the s.d. of technical triplicates.

We first investigated the effect of cargo transcription rate on Ty1 transposition. Although strong promoters (such as *pTDH3*) are desirable for cargo overexpression, their high transcription rate may interfere with that of *pGAL1*, thus lowering transposition rate and library size. Out of three yeast promoters (*pCYC1*, *pTEF1* and *pTDH3*, representing low, medium and high transcriptional output, respectively^28^; Supplementary Fig. 1a), we observed the highest transposition rate when *pTEF1* drove expression of *URA3* (Figs 2b and 3a). We used this promoter in future benchmarking experiments.

### Rrm3 deletion increases transposition rate

Ty1 replication is known to be highly regulated by various host factors^29^. To evaluate a coupling between genotype and function, we performed an extensive literature search and used the yeast haploid knockout collection to identify knockout phenotypes, which enabled increased rates of Ty1 replication^29^,^30^,^31^. Of the various genotypes tested (Fig. 3b), deletion of *rrm3* most significantly increased transposition rates in *S. cerevisiae* BY4741 (Fig. 2b). Rrm3p plays a role in DNA repair, which may influence retrotransposition, as it is dependent on homologous recombination^29^. Several other combinations of targets were evaluated, but these combinations did not exceed the transposition rate beyond that of *Δrrm3* alone (Fig. 3b). Therefore, *S. cerevisiae* BY4741 *Δrrm3* was used for all subsequent experiments.

### Genomic integration of Ty1 decreases transposition

Subsequently, we elected to move the retroelement into the genome, to gain a more accurate picture of retroelement behaviour in its final context. We used BY4741 *Δrrm3* with a genomically integrated, *pTEF1*-containing retroelement at high optical density (OD) and determined that the transposition rate was significantly inhibited compared with the plasmid-based retroelement (Fig. 2b).

### Transposition at high cell density increases library size

In all initial experiments, Ty1 transposition was induced when cells were at a low OD and continued as cells divided. However, this growth can significantly reduce effective library sizes, as mutations that occur early during growth can dominate the resulting culture during outgrowth^32^. We aimed to increase library sizes by inducing transposition for the same length of time (3 days), but at a much higher initial cell density (OD_600_=1). In this condition, additional cell growth would have a greatly reduced effect on library size. This condition significantly increased the retrotransposition rate (Fig. 2b) and all subsequent inductions were carried out at high cell density.

### Reducing induction temperature increases transposition

We next made use of the known temperature sensitivity of Ty1 (ref. 33) by inducing transposition at a lower temperature (22 °C). This modification greatly improved transposition rate (Fig. 2b). Interestingly, it also increased basal activation of our inducible Ty1 retroelement in the absence of a *cis-*encoded reverse transcriptase (Supplementary Fig. 1b), which could be due to the activation of endogenous Ty1 elements that are natively repressed at 30 °C.

### Increasing tRNA^iMet^ expression increases transposition

Based on real-time PCR experiments (Fig. 3c,d), we noted that induction of Ty1 RNA levels by *pGAL1* greatly exceeded resulting cDNA levels produced by *Ty1RT*. As Ty1 replication is primed by the yeast initiator methionine tRNA (tRNA^iMet^), we hypothesized that the concentration of this tRNA may be limiting transposition rates. By overexpressing tRNA^iMet^ from several promoters, we observed greatly improved transposition rates (Figs 2b and 3e). In particular, by overexpressing tRNA^iMet^ using its native promoter and terminator on a high-copy plasmid, transposition rate could be improved by ∼3.5-fold and this increase was accompanied by a corresponding increase in cDNA levels (Fig. 3f). All subsequent experiments used this overexpression strategy.

### Characterizing the effect of cargo length on transposition

As it is highly desirable to include long sequences consisting of multi-gene pathways in the inducible retroelement, the effect of transcript length on transposition rate was characterized. Specifically, we inserted gene fragments as additional cargo between the *URA3* reporter gene and the reverse transcriptase gene, and then measured the resulting transposition rates. In addition, we measured transposition rates after 3, 5 and 7 days of high cell-density induction in *S. cerevisiae* BY4741 *Δrrm3*. These experiments clearly revealed a negative correlation between cargo length and retrotransposition rate. However, lengthening the induction time from 3 to 7 days increased the number of retrotransposition events, especially for constructs containing the longest sequences (Fig. 3g). Importantly, relatively high transposition rates were maintained within approximately an order of magnitude as cargo size increased to roughly 5 kb, indicating that Ty1 is capable of generating diversity to a multi-gene pathway. It should be noted that this experiment combined each pathway element on the same mutagenesis cassette. However, it is also possible to distribute several multi-gene mutagenesis cassettes across the genome to enable simultaneous evolution on multiple segments of longer cargo.

### Characterizing the effect of terminators on transposition

When expressing multi-gene pathways in yeast, it is common to include a promoter before each gene and a terminator afterward. When inserting a multi-gene pathway into the Ty1 mutagenesis cassette, however, a terminator with bidirectional activity can significantly affect transposition, as the entire retroelement must be transcribed before reverse transcription. To characterize this effect, several native and synthetic terminators were inserted after the *URA3* reporter gene in the synthetic retroelement^34^. These experiments showed that including terminators inside the retroelement can lower the rate of transposition, with several terminators eliminating activity altogether (Supplementary Fig. 1c).

Although we did identify several terminators that reduced transposition rate to within one order of magnitude, we instead opted to use ribosome-cleavable 2A sites, which allow a single promoter to drive expression of a fusion peptide that then self-cleaves during translation^35^. This strategy allowed the evolution of multi-gene pathways, such as the xylose pathway evolved here, without including any terminators between genes. In addition, it allows multi-gene pathways to be expressed from a single promoter, reducing the length of DNA needed in the cargo and thus increasing the rate of transposition (Fig. 3g). Including 2A sequences as opposed to terminators thus allows our approach to attain a significantly higher library size for multi-gene pathways through two mechanisms: it avoids terminators and it reduces cargo length by only requiring a single promoter. However, it should be noted that 2A sites are as of yet unoptimized for use in yeast, such that cleavage efficiency may not be 100% in all contexts and thus may pose an issue for pathways in which the generation of fusion proteins would be undesirable. For these cases, we recommend the integration of multiple distinct mutagenesis cassettes into the same strain to enable the simultaneous directed evolution of pathway components.

### Measurement of mutation rate enabled by ICE

**Figure 4:**
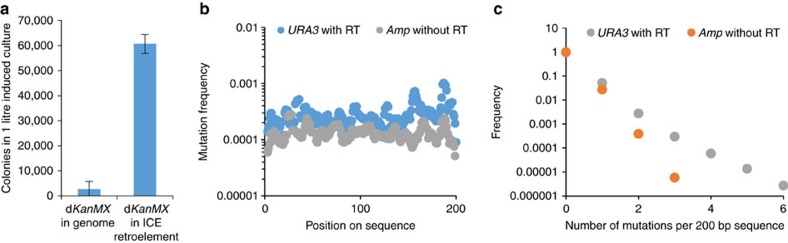
Measurement of Ty1 mutagenesis. (**a**) Characterization of d*KanMX* reversion with or without ICE retroelement integrated in the genome. Cells were exposed to galactose and then plated on G418-containing media to count colonies. For (**a**), error bars represent s.d. of technical triplicates. Mutation rates in Ty1 cDNA were determined through next-generation sequencing. Spatial distribution of mutation rates (**b**) and frequencies of observing a given number of mutations (**c**) of sequenced Ty1 cargo (*URA3*), as well as DNA not exposed to *Ty1RT* (*Amp*).

Next, we undertook a mutation reversion experiment to investigate the error rate of this approach in comparison with random drift/mutation. To do so, a non-functional *KanMX* antibiotic marker was constructed by inserting an artificial stop codon (generating d*KanMX*). Cells containing either a genomically integrated copy of d*KanMX* (as a control for random drift/adaptive evolution) or a copy integrated as cargo in the optimized Ty1 retroelement (the ‘mutagenesis cassette') were exposed to galactose and plated on G418-containing media. The number of G418-resistant colonies observed conservatively demonstrated a 20-fold higher reversion rate using *in vivo* mutagenesis over random drift (Fig. 4a). In addition, sequencing isolated colonies from this experiment demonstrated that out of 49 sequenced resistant colonies, 43 were found to have a mutation reverting the artificial stop codon (Supplementary Table 1), thus demonstrating *in vivo-*generated mutations as the mode of action.

Next, we evaluated the full mutational rate and spectrum conferred during a single round of *in vivo* mutagenesis using next-generation sequencing. This analysis indicated that *URA3* was mutated at rates of 0.15 kb^−1^ (Fig. 4b,c and also see Supplementary Note 1), a value that generally agrees with previously reported *in vitro* values for transcription/reverse transcription in Ty1 (0.05–0.25 kb^−1^)^25^,^26^. Moreover, the observed *dKanMX* reversion rate is consistent with this mutation rate (see Supplementary Note 2) and indicates that this system can effectively sample up to 1.6 × 10^7^ distinct mutants per litre (Fig. 3g). This library potential is the highest reported for any *in vivo*, continuous directed evolution approach in yeast (Supplementary Table 2). The overall spectrum of Ty1RT-based mutagenesis was found to be similar to other commonly used *in vitro* and *in vivo* mutagenic polymerases (Table 1), and occurred uniformly along the cargo (Fig. 4b). This result demonstrates that the increase in mutation frequency achieved in this work is effective at evolving cargo of interest in a manner previously unexplored for eukaryotic systems.

With the *in vivo* mutagenesis system in place, we next investigated the utility of *in vivo-*generated libraries (along with different modes of selection) in the directed evolution of three broad classes of genetic cargo: small-molecule-converting enzymes, transcriptional regulators and multi-enzyme pathways (Fig. 1f). In these experiments, both regulatory regions and coding regions were subjected to mutation and selection (in contrast to common *in vitro* searches), to enable the evolutionary process to alter both expression and protein properties in its search for improved phenotypes, and to take advantage of the expanded library size afforded by this method. In each case, we used a genomically integrated Ty1 element in a BY4741 *Δrrm3* strain background, in which the cargo of interest was integrated between *Ty1RT* and the 3′-LTR in the reverse orientation relative to Ty1 transcription and interrupted by an intron to facilitate mutant recovery. During evolution of *URA3* and *SPT15*, top mutants from each round were isolated and re-introduced to a fresh strain to eliminate any concurrent strain adaptation, whereas evolution of xylose catabolism proceeded in a continuous manner, demonstrating ICE. The intent of these experiments was to investigate the breadth of evolvable cargo and the variety of experimental designs compatible with retrotransposon-generated libraries. It is clear that each of these experiments can serve as a launching point for further in-depth mechanistic analyses of mutant function, as well as testing of different and subsequent evolutionary trajectories. As a mutagenesis technique, this approach is inherently phenotype agnostic and could be further applied to additional targets that require alternative screening modalities.

### Evolution of improved Ura3p substrate specificity

**Figure 5:**
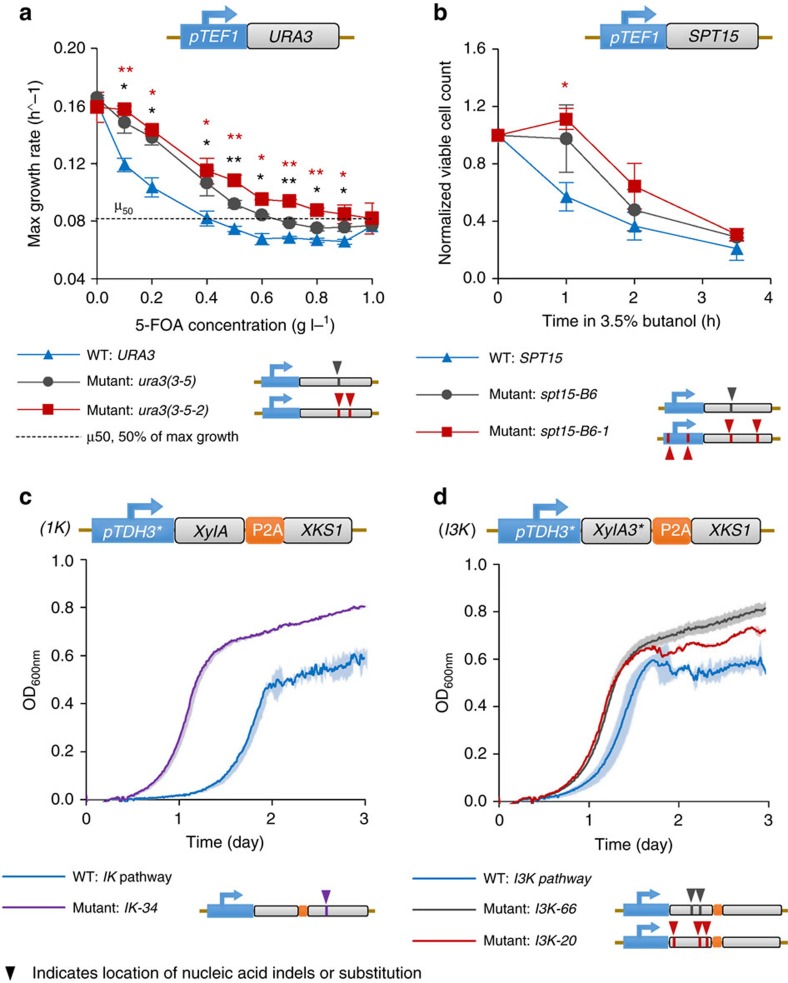
Implementation of *in vivo*-generated libraries for directed evolution of three distinct classes of genetic cargo. Three broad classes of genetic cargo, transcriptional regulators, single enzymes and multi-gene pathways, were used as targets for *in vivo* mutagenesis. (**a**) *URA3* variants with altered specificity were characterized by growth rate in uracil-deficient, 5-FOA containing media. (**b**) *spt15* variants were characterized by survival in killing concentrations of 1-butanol. (**c**,**d**) Xylose pathway variants were characterized through growth rate in xylose-containing media. *IK* pathway variants are pictured in **c** and *I3K* pathway variants are pictured in **d**. In **a** and **b**, error bars represent the s.e.m. derived from biological triplicates, one grey star indicates significantly higher values for the first round mutant versus wild-type at *P*<0.05 via a Mann–Whitney *U*-test, two grey stars indicate significantly higher values for the first round mutant versus wild-type at *P*<0.005 via a Mann–Whitney *U*-test, one red star indicates significantly higher values for the second round mutant versus wild-type at *P*<0.05 via a Mann–Whitney *U*-test and two red stars indicate significantly higher values for the second round mutant versus wild-type at *P*<0.005 via a Mann–Whitney *U*-test. In **c** and **d**, shaded areas represent the s.d. of biological triplicates.

Our first target involved engineering the substrate specificity of *URA3*, which encodes orotidine-5′-phosphate decarboxylase. Ura3p is an efficient catalyst that converts orotidine-5′-phosphate to uridine-5′-phosphate, yet also converts 5-fluoro-orotic acid (5-FOA) into 5-fluorouracil, a toxic compound. As a means to demonstrate the ability to modify enzyme promiscuity in a novel manner, we sought to isolate *URA3* variants with a decreased ability to convert 5-FOA to 5-fluorouracil, while maintaining their ability to enable uracil biosynthesis. Such variants have not, to our knowledge, been reported. To improve enzyme specificity for orotidine-5′-phosphate, we undertook simultaneous induction of mutagenesis and selection in uracil-deficient, 5-FOA-containing media, after which top variants were re-transformed into a fresh strain to exclude any adaptive, genomic mutations (see Methods). After two rounds, we isolated the best mutant, *URA3(3-5-2)*, which significantly outperformed wild type (*P*<10^−5^, Fisher's method) and which conferred a 2.5-fold increased IC_50_ on 5-FOA to a fresh strain relative to a strain expressing the wild-type enzyme (Fig. 5a). This variant contained two coding mutations (Arg^145^Ile and Arg^186^Lys) (Supplementary Fig. 2a and Supplementary Note 3). Although further *in vitro* analysis of these variants may reveal the underlying mechanistic basis for improved specificity, this experiment indicated that *in vivo*-generated libraries can be used to evolve substrate specificities using simultaneous mutagenesis and selection.

### Comparison of ICE to error-prone PCR

**Figure 6:**
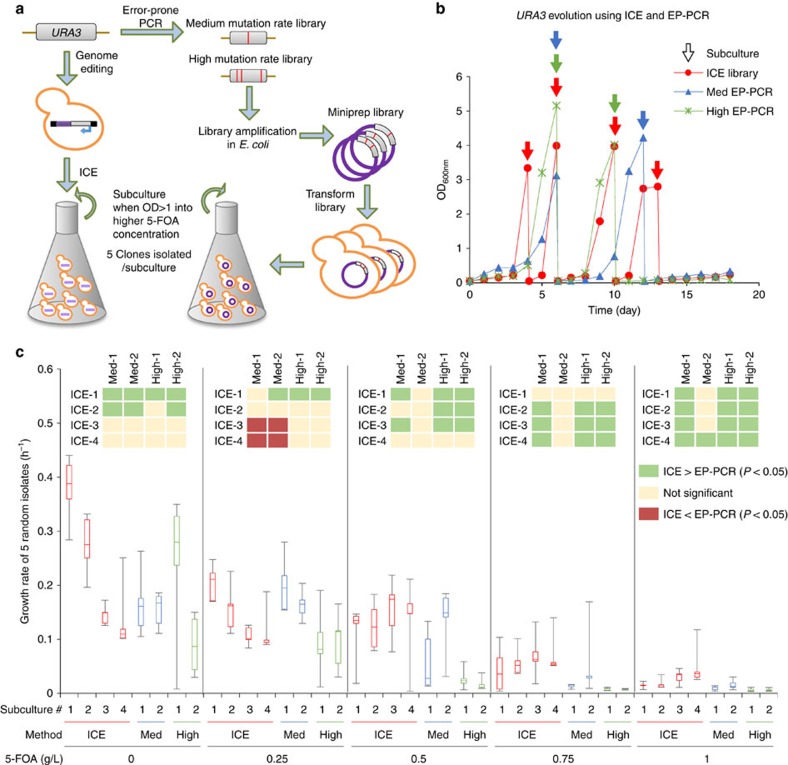
Comparison of ICE to error-prone PCR. The ability of ICE to generate 5-FOA-resistant mutants of *URA3* was evaluated in parallel with error-prone PCR. (**a**) Workflow for parallel evolution experiment is diagrammed. (**b**) Culture cell densities were monitored as selections progressed. Each time the OD_600_ of the culture exceeded 3, a portion was transferred to a new flask containing a 0.25 g l^−1^ increased concentration of 5-FOA (denoted by the presence of a coloured arrow). (**c**) Five random clones were isolated from each subculture (denoted by the number after the dash, for example, ICE-3 refers to clones isolated from the ICE library in subculture 3) and their growth was compared in varying concentrations of 5-FOA. Lines on boxplots in **c** represent, from top to bottom, maximum observed value, 75th percentile, median, 25th percentile and minimum observed value. *P*-values used to generate heatmaps were computed using a Mann–Whitney *U*-test.

Using this example, we sought to further compare the results obtained here using *in vivo*-derived mutagenesis with traditional *in vitro* mutagenesis. To this end, we generated two libraries of *URA3* variants through error-prone PCR using standard approaches, resulting in libraries of two differing mutagenic rates each roughly 10^5^ in size (see Methods). These libraries were selected head-to-head with the *in vivo* mutagenesis libraries in uracil-deficient, 5-FOA-containing media with equivalent cell density trigger thresholds for subculturing (Fig. 6a and also see Methods). On the basis of bulk growth rate and subculturing frequency, it was clear that the *in vivo* mutagenesis-derived libraries outperformed the error-prone libraries (reaching the threshold for subculturing four times versus two over the experimental time course; Fig. 6b). We next isolated five clones from each library/subculture and compared their growth in 5-FOA concentrations ranging from 0 to 1 g l^−1^ (Fig. 6c and Supplementary Fig. 4a,b). We found that the isolated strains derived from *in vivo* mutagenesis significantly (*P*<0.05, Mann–Whitney *U*-test) outperformed those derived from *in vitro* mutagenesis in the majority (125/240) of possible comparisons (growth rate, maximum OD_600_ and lag time). In 232/240 comparisons, *in vivo* mutagenesis was on par or better than traditional *in vitro* mutagenesis. This combined result is highly significant (*P*<10^−13^, Fisher's method), demonstrating the utility of an *in vivo* mutagenesis approach. These two results indicated (1) that Ty1-generated libraries yield improved variants faster than error-prone PCR and (2) that Ty1-derived mutants significantly outperform those derived from error-prone PCR.

### Spt15p evolution for improved 1-butanol tolerance

Our second target was the gene encoding the global transcriptional regulator Spt15p, the TATA-box binding protein^36^. Traditional *in vitro-*based evolution of this master transcriptional regulator has successfully improved complex phenotypes such as ethanol tolerance^6^, but no mutants have been reported which confer increased butanol tolerance. Here we aimed to use retrotransposon-generated libraries of *spt15* to identify dominant mutants conferring increased tolerance to 1-butanol (see Methods) with each round consisting of mutagenesis in a fresh strain background and selection in non-inducing conditions. Through two subsequent, iterative rounds, we were able to identify a variant (*spt15-B6-1*) that improved the tolerance of a fresh strain nearly twofold after 1 h in 3.5% 1-butanol (Fig. 5b, *P*<0.05, Mann–Whitney *U*-test). This mutant also improved growth of a fresh strain of yeast upwards of 44% in 1.4% 1-butanol (Supplementary Fig. 3h, *P*<0.05, Mann–Whitney *U*-test). Although we selected for tolerance, not growth rate, the collective performance of this mutant indicated that it significantly outperformed wild type (*P*<0.01, Fisher's method) in both tolerance and growth characteristics. This mutant contained two coding mutations (Arg^98^His and Gly^192^Ser) near the DNA-binding domain and two indel mutations in the promoter region (Fig. 5b, Supplementary Fig. 2b and Supplementary Note 4). These results indicate the potential of our approach to simultaneously mutate coding and regulatory elements. Moreover, this approach also generated these input libraries in a single, highly automatable step (transfer to galactose-containing media) with significantly reduced labour intensity compared with prior *in vitro* approaches with this transcription factor^6^.

### Pathway evolution for improved xylose catabolism

Our third and most complex target was the optimization of a multi-enzyme pathway containing a promoter, an isomerase and a kinase to enable increased xylose catabolism. Xylose catabolism is an industrially important phenotype for lignocellulose conversion^37^ and functional pathways have been established using an evolved xylose isomerase gene from *Piromyces spp.* (*xylA*)^38^ and enhanced through overexpression of xylulokinase (*XKS1*)^39^. As either of these two enzymes could serve as rate-limiting steps in this pathway, we established a multi-gene cassette encompassing a strong hybrid *TDH3* promoter (UAS_TEF_-UAS_CIT_-UAS_CLB_-P_TDH3_, referred to here as pTDH3*)^40^ driving these two coding regions through the use of a P2A site (see Supplementary Note 5)^35^. In a second, parallel evolution experiment, we substituted wild-type *XylA* for a previously identified mutant, xylA3*, that was shown to confer a 77% increase in xylose consumption rate^38^. These pathway constructs are named *IK* and *I3K*, respectively. Although *XylA* has been the focus of prior directed evolution studies, in no case has directed evolution been reported on the entire *XylA*-*XKS1* pathway simultaneously. For both arrangements, we aimed to identify mutations in a purely *in vivo*, continuous manner without intermediate re-transformation steps (Supplementary Fig. 5c) to demonstrate full continuous evolution. Thus, one continuous round of simultaneous mutagenesis and selection in ICE comprised many potential independent cycles.

After ICE of the *IK* multi-gene cassette over the course of one week, a superior isolate emerged, *IK-34*, which contains one coding mutation, Glu^164^Lys, in *xks1*. This isolate displayed a 21% increase in exponential growth rate (Fig. 5c) over the control and an 18 h shorter lag phase (Fig. 5c and Supplementary Fig. 3d). For *I3K*, two similarly performing superior isolates, *I3K-66* and *I3K-20*, emerged that both contain coding mutations in xylA3*. In these isolates, xylA3* contains one (Ile^433^Val, in addition to A1029G, a silent nucleotide change) and three (Ala^48^Ser, Ile^433^Val and Met^435^Ile) amino acid substitutions, respectively, and they display 14% and 16% improvements to exponential growth rate, respectively, (Fig. 5d) concomitantly with a 6 h decrease in lag time over wild-type *I3K*. It is interesting to note that the best mutants emerging from these two multi-gene cassettes were distinct (favouring xylose isomerase in one case and xylulokinase in the other), indicating the context-specific nature inherent to directed evolution. We then demonstrated through *in vitro* kinetic assays differences in the function of these mutant enzymes (see Supplementary Note 6). Taken together, performing ICE on entire catabolic pathways resulted in several proficient strains containing multiple mutations, which span the entire 4.6 kb pathway after just 1 week of continuous mutagenesis and selection, which is currently the longest pathway for which *in vivo* continuous directed evolution has been undertaken. As a result, we realized a significant acceleration to the process of creating pathway-wide mutants as compared with classical directed evolution techniques that require creating separate libraries of each gene and reassembling pathways *in vivo*^41^.

### Implementation of ICE in alternative yeast strains

**Figure 7:**
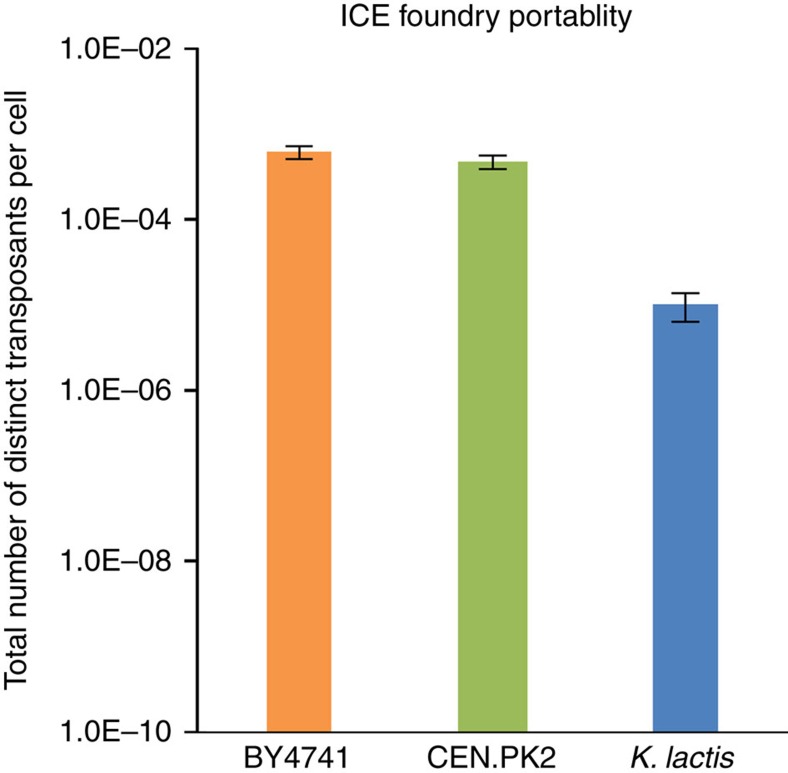
Transposition rates for alternate yeast strains. Transposition rates for wild-type BY4741 (low OD plasmid induction), CEN.PK2 (low OD plasmid induction) and *K. lactis* (high OD genomic induction). Error bars at the low OD condition represent 95% confidence intervals obtained from biological triplicates, whereas error bars at the high OD condition represent the s.d. of biological triplicates.

Finally, we wished to investigate the portability of the retrotransposon-assisted mutagenesis approach across divergent species of yeast. Specifically, we observed that alternative strains of *S. cerevisiae* such as CEN.PK2, as well as divergent species of yeast such as *Kluyveromyces lactis* were also able to support replication of the synthetic retroelement with only minimal modifications (Fig. 7). However, beneficial knockouts found in *S. cerevisiae* BY4741 did not transfer to CEN.PK2 and the lack of a knockout collection in this strain prohibited us from exploring this organism for further improved backgrounds. As Ty1 activity in *K. lactis* has only been recently demonstrated^42^ and the precise mechanism of Ty1 transposition in *K. lactis* is still uncharacterized, these preliminary results motivate future work investigating the species dependence of Ty1. Nevertheless, this result indicates that the general approach of using retrotransposons to undertake *in vivo* mutagenesis may be expanded to any other eukaryote which supports LTR retrotransposon activity^22^, making this a potentially powerful, broad-host approach.

## Discussion

In this work, we developed an *in vivo* mutagenesis system for yeast and applied it to the directed evolution of small-molecule-converting enzymes, regulatory proteins and metabolic pathways. Significantly, we have shown that this enables large, diverse mutant libraries in a continuous process, which is significantly faster, more effective, cheaper and less labour intensive than traditional *in vitro* techniques. Moreover, we have demonstrated that the ICE approach can surpass a traditional error-prone PCR library in both library and clone performance. Motivated by its utility demonstrated by this work, several challenges of ICE in its current form can be addressed to further its speed and effectiveness. Specifically, increasing the error rate for small cargo, increasing library sizes when evolving more than 5 kb, reducing the reintegration of mutants into alternative loci and reducing the potential for concurrent strain adaptation under certain modes of selection are goals for future designs. Nevertheless, this approach in its current instantiation is capable of supporting continuous evolution of parts and pathways as demonstrated here. This ICE approach complements powerful continuous and genome-scale engineering techniques in other organisms (Supplementary Table 2)^13^,^17^,^20^, can interface with any screening/selection technique and is the first demonstration of an ICE approach for optimization of small-molecule-converting enzymes and pathways (of which *XylA*-*XKS1* is the longest yet reported for any *in vivo* continuous directed evolution approach). Taken together, this work introduces the retroelement-assisted continuous evolution paradigm, demonstrates its utility for the directed evolution of a wide variety of phenotypes and indicates its unique potential to enable powerful new applications for the rapid evolution of cellular components across varied eukaryotic hosts.

## Methods

### Growth and transformation procedures for *E. coli* and yeast

For a summary of the following information in tabular form, see Supplementary Table 4. Yeast expression vectors were propagated in *E. coli* DH10β. *E. coli* strains were routinely cultivated in LB medium (10 g l^−1^ tryptone, 5 g l^−1^ yeast extract and 10 g l^−1^ sodium chloride) (Teknova) at 37 °C with 225 RPM orbital shaking. LB was supplemented with 100 μg ml^−1^ ampicillin (Sigma) when needed for plasmid maintenance and propagation. Yeast strains for which maintenance of auxotrophic markers was unnecessary were propagated in YPD (10 g l^−1^ yeast extract, 20 g l^−1^ peptone and 20 g l^−1^ glucose), YPG (10 g l^−1^ yeast extract, 20 g l^−1^ peptone and 20 g l^−1^ galactose) or YPX (10 g l^−1^ yeast extract, 20 g l^−1^ peptone and 20 g l^−1^ xylose). When required for plasmid maintenance, yeast strains were cultivated on a yeast synthetic complete (YSC) medium containing 6.7 g of Yeast Nitrogen Base (Difco) per litre, 20 g glucose per litre and a mixture of appropriate nucleotides and amino acids (CSM, MP Biomedicals, Solon, OH). For YSC medium containing galactose or xylose, glucose was omitted from the above recipe and replaced with 20 g l^−1^ galactose or xylose, respectively. All components were supplemented with 1.5% agar for solid media.

For *E. coli* transformations, 25 μl of electrocompetent *E. coli* DH10β (ref. 43) were mixed with 30 ng of ligated DNA and electroporated (2 mm Electroporation Cuvettes (Bioexpress) with Biorad Genepulser Xcell) at 2.5 kV. Transformants were rescued for 1 h at 37 °C in 1 ml SOC Buffer (Cellgro), plated on LB agar and incubated overnight. Single clones were amplified in 5 ml LB medium and incubated overnight at 37 °C. Plasmids were isolated (QIAprep Spin Miniprep Kit, Qiagen) and confirmed by Sanger sequencing.

For yeast transformations, 50 μl of chemically competent *S. cerevisiae* BY4741 were transformed with 1 μg of each appropriate purified plasmid according to established protocols^44^, plated on the appropriate medium and incubated for 3 days at 30 °C. Single colonies were picked into 1 ml of the appropriate medium and incubated at 30 °C. Plasmids were isolated from yeast using a Zymoprep Yeast Miniprep Kit II (Zymo Corporation) and transformed into *E. coli* for further amplification.

### Ligation cloning procedures

PCR reactions were performed with Q5 Hot-Start DNA Polymerase (NEB) according to the manufacturer's specifications. Digestions were performed according to the manufacturer's (NEB) instructions, with digestions close to the end of a linearized strand running overnight and digestions of circular strands running for 1 h at 37 °C. PCR products and digestions were purified with a QIAquick PCR Purification Kit (Qiagen). Phosphatase reactions were performed with Antarctic Phosphatase (NEB) according to the manufacturer's instructions and heat inactivated for 15 min at 65 °C. Ligations (T4 DNA Ligase, Fermentas) were performed overnight at 22 °C followed by heat inactivation at 65 °C for 20 min.

### Recombination cloning in yeast

One microgram of each PCR fragment was digested with DpnI and co-transformed into *S. cerevisiae* BY4741 according to the procedure described in ref. 44. This transformation mixture was then plated on the appropriate dropout medium and allowed to grow for 3 days at 30 °C. Yeast colonies from this plate were scraped and plasmids were extracted (Zymoprep Yeast Miniprep Kit, Zymo Research). This plasmid mixture was then transformed into *E. coli* DH10β and plated. Individual colonies were then amplified in liquid culture and plasmids were extracted. Correctly assembled plasmids were confirmed through restriction digestion and sequencing.

### Gibson assembly

Isothermal assembly of DpnI-digested PCR fragments was performed according to manufacturer's (NEB) instructions^45^.

### Vector and strain construction

All strains and vectors listed in this study were assembled according to the schemes listed in Supplementary Tables 5–14.

For all knockouts, a loxP-*KanMX*-loxP deletion cassette was constructed from plasmid PUG6 (ref. 46). One kilobase of homologous sequence was amplified from the region 5′ to the desired integration site and ligated at the 5′-end of the loxP-*KanMX*-loxP module. A second kilobase of homologous sequence amplified from the region 3′ to the integration site was then ligated at the 3′-end of the loxP-*KanMX*-loxP module. The whole gene disruption cassette was then amplified and transformed into *S. cerevisiae* BY4741 or CEN.PK2, using a standard lithium acetate transformation method^44^ or a version optimized for CEN.PK2 (ref. 46), respectively. Cells were then plated onto YPD plus G418 plates (200 μg ml^−1^ G418). After 1 day of growth, microcolonies were replicated onto new YPD plus G418 plates. The resulting colonies were amplified in 3 ml YPD+G418 and the genomic DNA was extracted using the Wizard Genomic DNA Purification kit (Promega). Correct knockouts were confirmed by PCR.

Confirmed single knockout strains were transformed with the Cre expression plasmid pSH47 (ref. 44). Cre recombinase was then induced by incubating cells in YPG medium for 24 h. Cells were subsequently streaked onto YPD and replica-plated onto YPD plus G418, to isolate colonies in which the G418 marker (*KanMX*) had been excised. The Cre expression plasmid in G418-sensitive colonies was removed by incubating cells in YPD plus 5-FOA for 24 h, thus excising the *URA3*-marked plasmid and yielding a clean version of the knockout strain with a single loxP site in the chromosome. Sequential gene knockouts were performed with the same protocol using this clean (*KanMX*-less) strain as a template, yielding a double-knockout strain.

For *K. lactis*, one kilobase of homologous sequence was amplified from the region 5′ to *KlURA3* with a short linker on the 3′-end. A second kilobase of homologous sequence was amplified from the region 3′ to *KlURA3* with the same short linker on the 5′-end. After Gibson assembly, the whole disruption cassette was PCR amplified and transformed into *K. lactis* CBS 2359 using the standard lithium acetate method. Cells were allowed to recover in YPD overnight, then were plated on YPD plus 5-FOA plates. After 5 days, colonies were replicated onto YPD plus 5-FOA and YSC−Ura plates. Correct knockouts were confirmed by growth on YPD plus 5-FOA, the absence of growth on YSC−Ura and by colony PCR.

Genomic integrations of engineered retroelements into the *TRP1* locus were performed with the aid of Cas9p^18^. Briefly, the parent strain was transformed with a *URA3*-marked plasmid expressing *CAS9* from the *pGAL1* promoter and a guide RNA targeted to the *TRP1* locus. Transformants were then transformed using standard procedures with a PCR product containing the retroelement of interest as well as homology to *TRP1*. This transformation mixture was then plated on media containing galactose to induce *CAS9* expression. Successful knock-ins were confirmed with PCR and the *CAS9* plasmid was excised through culture in 5-FOA.

### Analysis of transposition efficiency

For the following, it should be noted that the synthetic Ty1 retrotransposon (whether integrated on a plasmid or on the genome) contained an adjacent *HIS3* gene used for plasmid maintenance and as a selectable marker for genome integration, respectively. Therefore, we cultured all strains in histidine dropout media, to facilitate media equivalence between all tested strains. As described in the main text, the synthetic Ty1 retrotransposon contained an intron-containing *URA3* gene in the reverse orientation to provide a *URA3*^*+*^ phenotype if and only if retrotransposition occurs. Therefore, we plated induced cultures on media lacking histidine and uracil to count the number of cells containing an intact *URA3* gene, and hence which had a parent that underwent retrotransposition.

For low cell-density induction, three biological replicates of a yeast strain carrying the engineered retrotransposon of interest were used to inoculate 50 ml liquid cultures lacking histidine and containing galactose, thus inducing retroelement transcription. After 3 days of growth at 30 °C, cultures were plated on agar containing glucose and either lacking histidine or lacking both histidine and uracil, and allowed to grow for 3 days at 30 °C. Colonies were counted manually or through automated software^47^ and counts were used as inputs to the Fluctuation Analysis Calculator^48^ implementing the Ma *et al*.^49^ maximum likelihood estimation method. Calculated mutation rates per cell were divided by the time spent in galactose medium, to determine the transposition rate per cell per generation, as well as 95% confidence intervals. This value was then used to estimate a library size.

For high OD tests with *S. cerevisiae*, cells were first cultivated in 50 ml liquid cultures lacking histidine and containing glucose, and then resuspended in 50 ml liquid cultures lacking histidine and containing galactose to an initial OD of 1. After 3 days of growth at 30 °C, cultures were plated on agar containing glucose and either lacking histidine or lacking both histidine and uracil, and allowed to grow for 3 days at 30 °C. Colonies were counted manually or through automated software^47^ and counts were averaged. This average was used as an estimate for the number of transpositions, which occurred during the experiment, and this average was divided by the total number of cells, which had been exposed to galactose to obtain a per-cell measure of transposition rate.

For high OD tests with *K. lactis*, cells were cultivated in 2 ml liquid cultures of YSC plus 400 mg l^−1^ G418 containing glucose and then resuspended in 2 ml liquid cultures containing galactose to an initial OD of 1. After 3 days of growth at 22 °C, cultures were plated on YSC agar containing glucose and lacking uracil, and allowed to grow for 3 days at 30 °C. Colony counts and transposition estimates were performed as described above for *S. cerevisiae*.

### Quantitative PCR analysis

For determination of retroelement messenger RNA and cDNA levels, yeast strains carrying the appropriate retroelement were grown to mid-log phase (OD=0.5) in 5 ml YSC containing either glucose or galactose and lacking the appropriate amino acids. Total RNA was extracted (Ribopure Yeast Kit, Life Technologies) from half of each culture and converted to cDNA (High Capacity cDNA Reverse Transcription Kit, Life Technologies). Total DNA was extracted (Wizard Genomic DNA Purification Kit, Promega) from the other half of the culture. Quantitative PCR (qPCR) was conducted using 10 ng of either cDNA or total DNA (FastStart SYBR Green Master, Roche) using primers specific for an intronless *URA3* (*URA3*RTPCRF and *URA3*RTPCRR) and with *ALG9* as an internal standard (Alg9F and Alg9R).

### Next-generation sequencing

Ten replicates from BY4741 *Δrrm3* plus pGALmTy1-*Ty1RT*-*URA3I*-*pTEF1* were cultivated in 50 ml liquid cultures lacking histidine and containing glucose. After 3 days of growth at 30 °C, 1 ml culture was removed and the plasmids were extracted using Zymoprep Yeast Plasmid Miniprep Kit II (Zymo Research). The rest of the culture was then resuspended in 50 ml liquid cultures lacking histidine and containing galactose to an initial OD of 1. After 3 days of growth at 30 °C, 1 ml culture was extracted to obtain plasmids and 1 ml culture was plated on agar containing glucose, and either lacking histidine or lacking both histidine and uracil, and allowed to grow for 3 days at 30 °C. Colonies were counted manually or through automated software^50^ and counts were averaged. This average was then used as an estimate for the number of transpositions, which occurred during the experiment. Two sequencing primer pairs with different barcodes were used to amplify the ampicillin sequence region from fresh pGALmTy1-*Ty1RT*-*URA3I*-*pTEF1* plasmid and pGALmTy1-*Ty1RT*-*URA3I*-*pTEF1* plasmid extracted from yeast grown in glucose medium, and 20 barcoded primer pairs amplified the *URA3* sequence region from 20 minipreps of galactose cultures. The PCR products were purified and the concentrations were determined by nanodrop. A final concentration of 50 ng μl^−1^ sample was prepared by combining 22 PCR purified products, with a 5:2 molar basis of ampicillin amplicon to *URA3* amplicon. This mixture was then sequenced using an Illumina Miseq in 2 × 250 bp paired-end mode. All PCR fragments and their corresponding primers are listed below.

Paired-end reads were matched up and error-corrected using pandaseq^51^, using stringent quality filtering (threshold=0.95). Matched pairs were then divided up based on barcode sequence using sabre^52^, allowing for single-nucleotide mutations (as each barcode differed by at least 2 bp from all other barcodes) and barcodes were removed with the trimmingreads.pl script of the NGS QC toolkit^53^. After combining reads originating from the same culture into the same file, alignment to the unmutated amplicon was performed using ssaha2 (ref. 54). Custom python scripts were then used to extract the total number of mutations identified, their locations on the wild-type sequence and their frequency per mutant. All data originating from *URA3* was then averaged. Data in Table 1 was calculated by subtracting the mutation rates of the yeast-derived *Amp* amplicon from those of the averaged *URA3* amplicon. Ninety-five per cent confidence intervals for mutation counts were computed using the method of the Clopper–Pearson interval^55^.

### Analysis of ICE mutation rate using d*KanMX* reversion assay

Strains *S. cerevisiae* BY4741 *trp1*::Ty1-*Ty1RT*-d*KanMX*-*pTEF1* and *S. cerevisiae* BY4741 *trp1*::d*KanMX*-*pTEF1* were cultured in 2.0 ml YSC−His medium containing 2% glucose. These cells were then resuspended into 2.0 ml YSC−His containing 2% galactose at an OD of 1.0, in triplicate, for 3 days at 22 °C, to induce mutagenesis. each culture (1.0 ml) was then plated on YPD containing 400 mg l^−1^ G418. After 2 days, colonies were counted to establish the rate of reversion mutations. Thirty-nine colonies from *S. cerevisiae* BY4741 *trp1*::Ty1-*Ty1RT*-d*KanMX*-*pTEF1* were grown and genomic DNA was extracted. From each colony, the d*KanMX* marker was PCR amplified and sequenced to confirm the reversion mutations present.

### Evolutionary strategies

#### URA3 evolutionary strategy

During the first round of *URA3* evolution, a 50 ml culture containing *S. cerevisiae* BY4741 *Δrrm3 trp1::Ty1-Ty1RT-URA3I-pTEF1* was mutagenized for 3 days at 22 °C at stationary phase and then transferred to 500 ml of galactose-containing, uracil-deficient media, and allowed to grow (Supplementary Fig. 5a). Each day the OD of this culture attained a value >1, the culture was re-inoculated into fresh medium containing an increased concentration of 5-FOA (0, 0.1, 0.3, 0.5 and 1.0 g l^−1^). After each subculture, genomic DNA was isolated from a sample of bulk culture and mutants were recovered for sequencing.

For the second round of evolution, a 50 ml culture containing *S. cerevisiae* BY4741 *Δrrm3 trp1::Ty1-Ty1RT-URA3(3-5)I-pTEF1* was mutagenized for 3 days at 22 °C at stationary phase and then transferred to 500 ml of galactose-containing, uracil-deficient media with 0.3 g l^−1^ 5-FOA, and allowed to grow. Each day the OD of this culture attained a value >1, the culture was re-inoculated into fresh medium containing an increased concentration of 5-FOA (0.3, 0.5, 0.7 and 1.0 g l^−1^). After growth in 0.5 g l^−1^ 5-FOA, the culture was split and re-inoculated into either 0.7 or 1.0 g l^−1^ 5-FOA. After each subculture, genomic DNA was isolated from a sample of bulk culture and mutants were recovered for sequencing.

#### SPT15 evolutionary strategy

During the first round of *SPT15* evolution, 50 ml cultures of *S. cerevisiae* BY4741 *Δrrm3 trp1::Ty1-Ty1RT-SPT15I-pTEF1* were precultured in glucose media, after which they were induced in YSC−His+20 g l^−1^ galactose for 3 days (Supplementary Fig. 5b). Mutated cells were then pelleted and incubated in glucose-containing media for ∼2 h, to allow recovery and time for expression of *Spt15* mutants. Next, butanol was added to a final concentration of 2.5% and cells were incubated with shaking for 3 h in sealed flasks at 30 °C. At this point, genomic DNA was isolated from a sample of bulk culture and the *SPT15* gene was recovered for sequencing via PCR (see Mutant isolation).

To undertake a second round of ICE, cultures of *S. cerevisiae* BY4741 *Δrrm3 trp1::Ty1-Ty1RT-spt15-B6I-pTEF1* were induced in YSC−His+gal for 3 days, pelleted and incubated in glucose-containing media for ∼2 h, to allow recovery and time for expression of *spt15* mutants. Next, butanol was added to final concentration of 3.5% and cells were incubated with shaking for 3 h in sealed flasks at 30 °C. At this point, genomic DNA was isolated from a sample of bulk culture and the *SPT15* gene was recovered for sequencing via PCR (see Mutant isolation).

#### Xylose isomerase pathway evolutionary strategy

Strains *S. cerevisiae* BY4741*Δr-g-x trp1*::Ty1-*Ty1RT*-*XylAI*-P2A-*XKS1*-pTDH3* and *S. cerevisiae* BY4741*Δr-g-x trp1*::Ty1-*Ty1RT*-XylA3*I-P2A-*XKS1*-pTDH3* were cultured in 50 ml YSC−His medium containing 2% glucose (Supplementary Fig. 5c). These cells were then resuspended into 50 ml YSC−His containing 2% galactose at an OD of one for 3 days at 22 °C, to induce mutagenesis. Several 1 μl aliquots of culture were then plated on YSC−His or YPX plates. After 3 days, large colonies were identified. Large colonies were picked into a 96-well plate for growth analysis and clones exhibiting strong growth were subject to genomic DNA extraction and PCR, to sequence mutant pathways. In this way, three pathway mutants were isolated (Supplementary Table 3).

### Mutant isolation

Genomic DNA from a small (∼1 ml) volume of culture was isolated using crude total yeast DNA according to established procedures^56^. For *SPT15* and *URA3* selections, this DNA was pre-digested with AscI (which exists uniquely in our synthetic intron), to enable specific amplification of spliced copies of the cargo. In general, unspliced copies of the cargo also amplified due to incomplete digestion and thus spliced copies were isolated through gel extraction. Next, this extract was cloned into an expression vector for sequencing. For xylose pathway screening, pTDH3*-XylA(3*) and pTDH3*-XKS1 gene fragments were individually PCR amplified from total DNA extractions of isolates and cloned into an expression vector for sequencing.

### Methods of characterizing ICE mutants

#### *URA3* mutant growth analysis

First-round *URA3* mutants were tested by plating on solid media containing or lacking uracil and containing 0.3 g l^−1^ 5-FOA. After growth for 3 days, colony size was measured using a BioRad Gel Dock XR+ (Bio-Rad Laboratories, Inc.) and automated image analysis software^50^. After the second round of selection, the maximum rates of exponential growth were characterized for first- and second-round mutants using a Bioscreen C (Growth Curves USA). Briefly, selected strains were inoculated at OD=0.1 and OD measurements were taken every 15 min using continuous shaking for 3 days at 30 °C. Growth rates were calculated using a custom MATLAB script (available on request).

#### *URA3* mutant structural analysis

URA3p mutant structures were generated and visualized using PyMol with PDB 3GDK as a template. Rotamers introducing minimal steric clash were selected.

#### *SPT15* butanol tolerance testing

To evaluate the effects of the mutant *Spt15-B6* gene, a *pTEF1*-*Spt15-B6* cassette was cloned into a low-copy plasmid and transformed into a fresh strain of BY4741 *Δrrm3*. Two control strains were also made, containing a blank *pTEF1* plasmid and a *pTEF1*-*SPT15* wild-type plasmid. Each was grown up in media and then resuspended in fresh media at an OD of ∼0.05. The actual OD was measured and recorded, and then butanol was immediately added to the desired concentration. The culture tubes were sealed to prevent evaporation and the cultures were incubated at 30 °C. At various timepoints, the OD was measured and normalized against the original OD of each replicate culture.

For high-butanol testing, the same strains described above were precultured in glucose media, then diluted to an OD=0.05 and incubated overnight. The next day, they were resuspended at OD=1.0 in media containing either 3.5% or 3.7% butanol. A time-zero sample was immediately plated, to determine the number of cells in the culture. At intervals, a small volume was removed and diluted by 100-fold in water, then plated. On growth, the colonies were counted to create an estimate of how many cells remained alive. Each replicate was normalized to its own time-zero colony count.

#### Expression analysis

For determination of wild-type or mutant *SPT15* expression levels, cells expressing either *pTEF1*-*SPT15* or *pTEF1*-*Spt15-B6-1* were grown to early exponential phase and total RNA was extracted and converted to cDNA as described above for ‘Quantitative PCR analysis'. Quantitative reverse transcriptase–PCR was carried out using primers that spanned the *pTEF1* promoter and the *SPT15* gene (primers Spt15-qPCR2f and Spt15-qPCR2r), eliminating amplification of endogenous *SPT15*. *SPT15* cycle threshold numbers were compared with that of a reference gene (*ALG9*) as above.

#### *Spt15* mutant structural analysis

Spt15p mutant structures were generated and visualized using PyMol with PDB 1NH2 as a template.

#### Xylose pathway mutant growth analysis

Colonies identified from plate-based selection were streaked onto a fresh plate and inoculated in 0.5 ml YSC−His+glucose medium for a 96-deepwell plate growth test. The original strains without galactose induction were also inoculated in 0.5 ml YSC−His+glucose medium in a 96-deepwell plate as controls. All cultures were then transferred to 1 ml xylose medium for growth test at an initial OD of 0.01. Growth curves were monitored by withdrawing 120 μl culture into a 96-well plate at each time point, to read OD_600nm_ using a plate reader (Cytation 3, BioTek Instruments, Inc.). Isolates with improved growth rate were picked from the restreaked plate to extract genomic DNA as above. Each component of the pathway was then amplified and purified or ligated into p413-*pTEF1* for sequencing. Mutant strains were then inoculated in 1 ml YPX at an initial OD of 0.01 for growth test on a Bioscreen (Bioscreen C, Growth Curves USA).

#### Xylose isomerase kinetics analysis

For xylose isomerase kinetics analysis, mutant or wild-type strains were grown until mid-exponential growth phase (OD of 0.6–0.8) in selective media. Cells were collected by centrifuging at 3,000 *g* for 5 min at 4 °C. Total protein was extracted using Y-PER Plus (dialysable yeast protein extraction reagent, Thermo scientific) and protein concentration was determined using the Pierce BCA protein assay kit (Thermo Scientific). Xylose isomerase activity from these cell extracts was determined by measuring oxidation of NADH at 340 nm using a spectrophotometer^57^. Each 1 ml reaction contained 100 mM Tris-HCl pH 7.5, 0.15 mM NADH, 10 mM MgCl_2_, 2 U sorbitol dehydrogenase and the diluted protein samples. Kinetic parameters were determined within a range of 25–500 mM xylose. All enzyme assays were performed in biological triplicate.

#### XylA(3*) mutant structural analysis

Homology modelling of XylAp was performed by the Swiss-Model homology modelling server in automated mode and the resulting structure was refined through energy minimization^58^. Energy minimization was performed with the Tinker molecular mechanics package to a RMS of 0.01 kcal mol^−1^ Å^−1^ using the OPLS-aa force field and GBSA, to account for solvent effects^59^. Docking predictions were performed using this refined structure by Symmdock and GrammX for a dimer with C_2_ symmetry^60^,^61^. Six candidate dimer structures for XylAp were selected from among the results generated by these two servers on the basis of their similarity to existing xylose isomerase crystal structures and each candidate was then refined as above. These minimizations were run simultaneously on separate cores of an Intel core i7 processor. The dimer of lowest energy after minimization was selected as the template on which the mutations would be introduced. Mutations were introduced using PyMol and the rotamers introducing minimal steric clash were selected^62^. This mutant dimer was then refined as above. All protein structures were visualized with PyMol.

### Comparison of *in vivo* and *in vitro* mutagenesis

To compare the ICE-derived *URA3* library to one which could be obtained using traditional methods, error-prone PCR libraries of *URA3* were also constructed. *URA3* was amplified in an error-prone manner using the GeneMorph II Mutagenesis kit according to the manufacturer's instructions. This was done in duplicate to achieve either 4.5–9.0 mutations per kilobase (‘Medium') or 9–16 mutations per kilobase (‘High'). Both were ligated separately into a low-copy vector with the TEF1 promoter, to match the construct made for the ICE library. The mutant plasmid libraries of ∼10^5^ in size were then transformed into *E. coli* DH10-β using electroporation, which were then harvested from petri dishes. The plasmid libraries were then purified and transformed into wild-type By4741 yeast as described above. After transformation, the yeast libraries were grown to stationary phase. Concurrently, a new ICE-derived library of *pTEF1-URA3* was created as described above; however, with a reduced galactose-induction volume of just 1 ml. After 3 days of induction, the two error-prone libraries and the ICE library were each resuspended in uracil-deficient media containing 0.3 g l^−1^ 5-FOA to a final OD of 0.05 and allowed to grow. Each day the OD of this culture attained a value greater than ∼3.0, the culture was re-inoculated into fresh medium containing an increased concentration of 5-FOA (0.3, 0.5, 1.0, 1.5 and 2.0 g l^−1^). At each subculture, a sample was plated. For characterization of adaptation, five colonies from each plate were picked and grown to stationary phase. Their growth rates in media containing various concentrations of 5-FOA were characterized using a Bioscreen C (Growth Curves USA). Briefly, selected strains were inoculated at OD=0.1 and OD measurements were taken every 15 min using continuous shaking for 3 days at 30 °C. Growth rates were calculated using a custom MATLAB script (available on request).

### Flow cytometry

Yeast colonies were picked in triplicate from glycerol stock, grown in the appropriate medium to mid-log phase and analysed (LSRFortessa Flow Cytometer, BD Biosciences; excitation wavelength: 488 nm, detection wavelength: 530 nm). Day-to-day variability was mitigated by analysing all comparable transformants on the same day. An average fluorescence and s.d. was calculated from the mean fluorescence values for the biological replicates. Flow cytometry data was analysed using FlowJo software (FlowJo, LLC).

### Sample size and experimental repeatability

All qPCR experiments were run three times using the same sample to generate measures of variability, to bring our work into alignment with other work in the field. All other experiments containing error bars were performed on independent biological triplicates to generate measures of variability, to bring our work into alignment with other work in the field. No samples were excluded from our analyses. No sample randomization or investigator blinding was undertaken. All statistical tests used (one-tailed Student's *t*-test, unequal variances or one-tailed Mann–Whitney *U*-test) are in line with state-of-the-art work in the field. All experiments were attempted once.

### Code availability

All computer code is available on request.

### Data availability

The data that support the findings of this study are available from the corresponding author upon request. Sequencing data can be found at NCBI Sequence Read Archive under the accession code SRR4244056.

## Additional information

**How to cite this article:** Crook, N. *et al*. *In vivo* continuous evolution of genes and pathways in yeast. *Nat. Commun*. **7,** 13051 doi: 10.1038/ncomms13051 (2016).

## Supplementary Material

Supplementary Information Supplementary Figures 1-5, Supplementary Tables 1-14, Supplementary Notes 1-6 and Supplementary References

## Figures and Tables

**Table 1:** Mutation spectrum of *Ty1RT*-RNAPII compared with other polymerases.

	***Ty1RT*****-RNAPII**	**Mutazyme II**	**Taq**	**DNAQ926**	**TP-DNAP1**^**Y427A**^	**Yeast DNAP**
*Bias indicators*						
Ts/Tv	1.06	0.9	0.8	1.4	0.17	0.98
AT−>GC/GC−>AT	0.28	0.6	1.9	0.3	0.44	0.48
A,T−>N (%)	28.8	50.7	75.9	30	44.3	31.7
G,C−>N (%)	69.4	43.8	19.6	70	55.7	68.3
						
*Indels*						
Insertions (%)	0.08	0.7	0.3	N.R.	N.R.	3.01
Deletions (%)	1.6	4.8	4.2			
						
*Mutation frequency*						
Mutations per kb	0.15	3–16	4.9	0.05	0.00004	0.0000002

N.R., not reported.

DNAQ data taken from ref. 13. TP-DNAP1^Y427^ data taken from ref. 20. Yeast DNAP data taken from ref. 63.
