# Supplementary material for: In vivo continuous evolution of genes and pathways in yeast
Source: Nat Commun. 2016 Oct 17;7:13051. doi: 10.1038/ncomms13051 (PMC5071640; doi:10.1038/ncomms13051)
Supplement: Supplementary Information — Supplementary Figures 1-5, Supplementary Tables 1-14, Supplementary Notes 1-6 and Supplementary References [file ncomms13051-s1.pdf]

Supplementary Figures

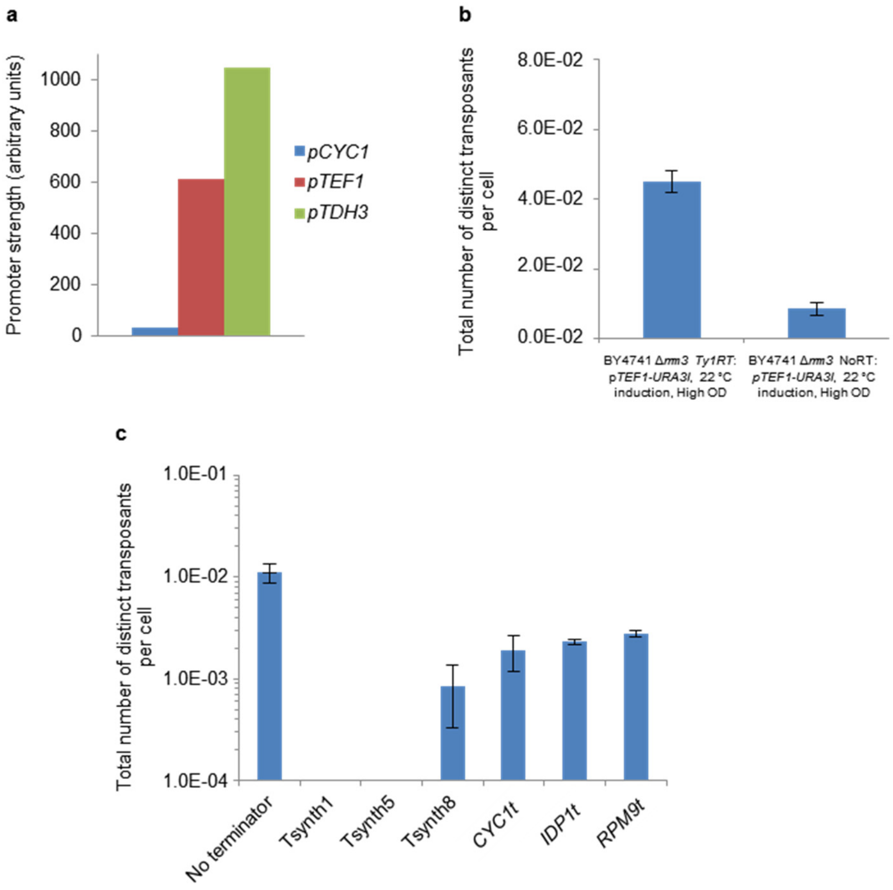

Supplementary Figure 1 | Improvement of Ty1 Transposition. **a**, Promoters used in this study. Indicated promoters were used to drive yellow fluorescent protein and fluorescence was measured using flow cytometry. **b**, Transposition of RT-containing and RT-less retroelements in a genomic context at 22ºC. **c**, The effect of terminators on transposition rate. No transpositions were observed for Tsynth1 or Tsynth5. Strains containing the appropriate retroelement were exposed to galactose at high OD for (**b**) and (**c**) for three days and then plated on uracil-deficient media to count transposants. Error bars in (**b**) and (**c**) represent the standard deviation of biological triplicates.

a

b

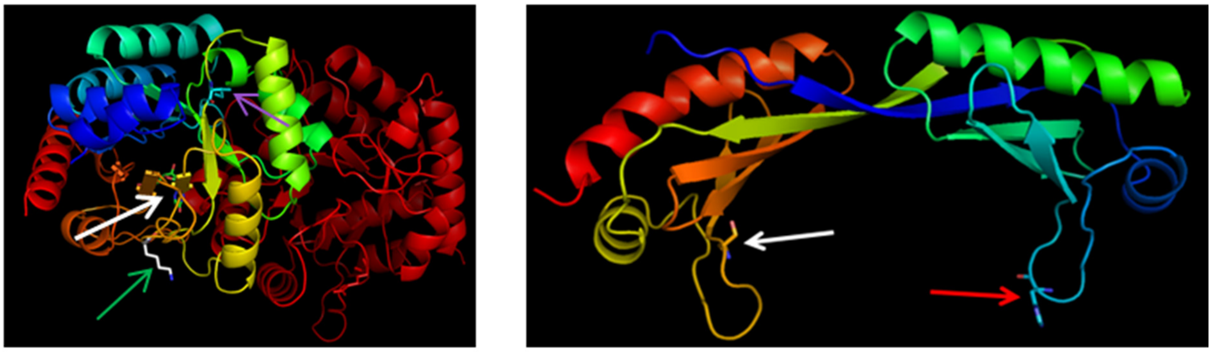

c

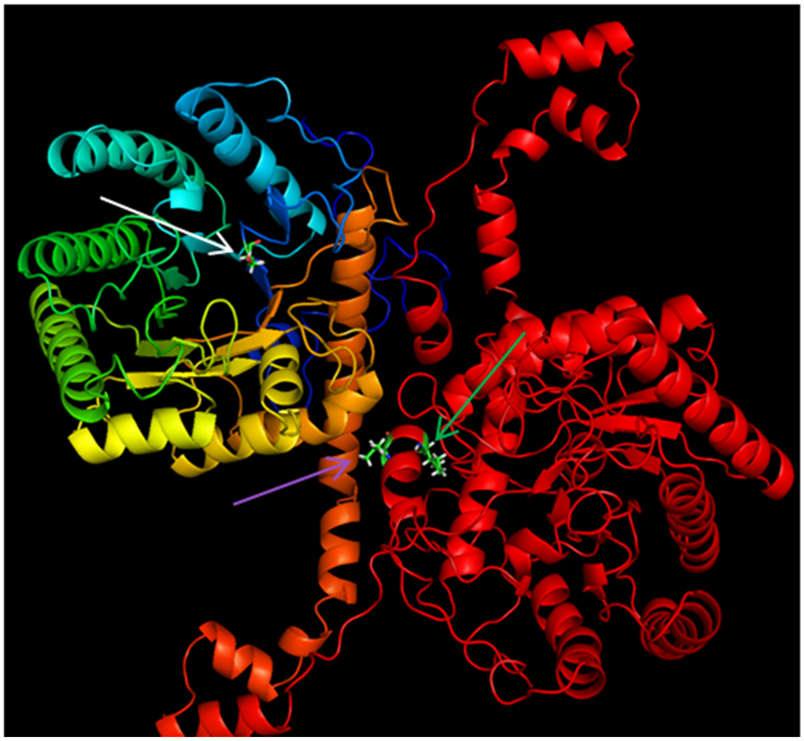

Supplementary Figure 2 | Predicted structure of ICE mutants. a, Predicted structure of the *ura3(3-5-2)p* homodimer. One monomer is colored red. The orotidine-5'-monophosphate ligand is shown in its catalytic site with a white arrow, the Arg^{145}Ile mutant is shown by the purple arrow, and the Arg^{186}Lys mutant is shown by the green arrow. **b**, Predicted structure of mutant *spt15-B6-1p*. The amino acid substitution Arg^{98}His indicated by the red arrow. The amino acid substitution Gly^{192}Ser which defines *spt15-B6-1* is indicated by the white arrow. **c**,

Predicted structure of the I3K-20 homodimer. One monomer is colored red. The Ala^{48}Ser mutation is indicated by the white arrow, the Ile^{433}Val mutation is indicated by the purple arrow, and the Met^{435}Ile mutation is indicated by the green arrow.

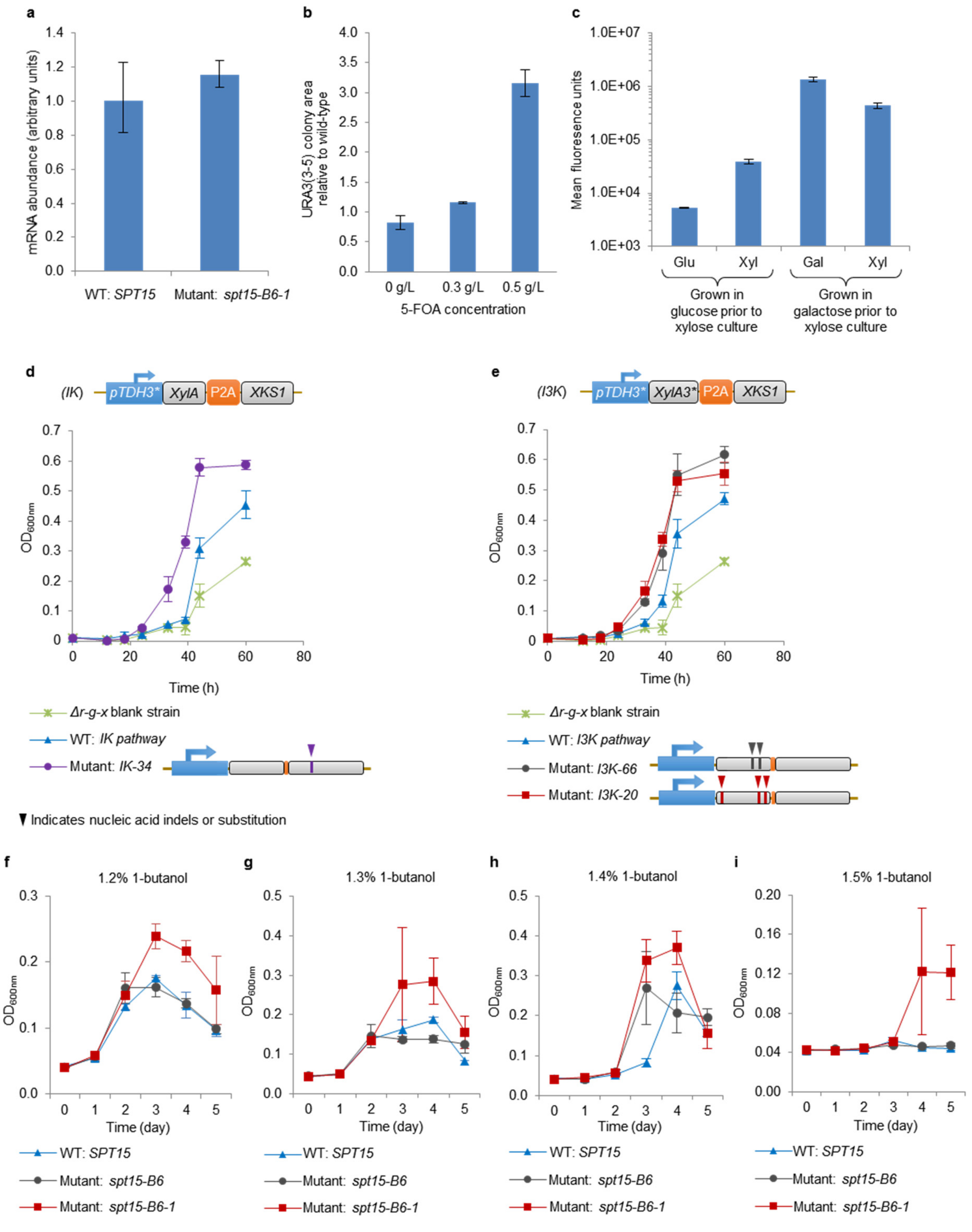

a
mRNA abundance (arbitrary units)
WT: SPT15
Mutant: spt15-B6-1
b
URA3(3-5) colony area relative to wild-type
0 g/L
0.3 g/L
0.5 g/L
5-FOA concentration
c
Mean fluoresence units
Glu
Xyl
Gal
Xyl
Grown in glucose prior to xylose culture
Grown in galactose prior to xylose culture
d
(IK)
pTDH3*
XylA
P2A
XKS1
OD600nm
Time (h)
Δr-g-x blank strain
WT: IK pathway
Mutant: IK-34
e
(I3K)
pTDH3*
XylA3*
P2A
XKS1
OD600nm
Time (h)
Δr-g-x blank strain
WT: I3K pathway
Mutant: I3K-66
Mutant: I3K-20
Indicates nucleic acid indels or substitution
f
1.2% 1-butanol
g
1.3% 1-butanol
h
1.4% 1-butanol
i
1.5% 1-butanol
OD600nm
Time (day)
WT: SPT15
Mutant: spt15-B6
Mutant: spt15-B6-1

Supplementary Figure 3 | Characterization of ICE mutants. a, Transcriptional rates of *SPT15* mutants. *SPT15* expression levels were measured through qRT-PCR. **b**, Growth of *URA3(3-5)*-containing strains in media lacking uracil and containing 5-FOA. Indicated strains were plated on solid media lacking uracil and containing the indicated concentration of 5-FOA. Colony area was measured using automated image analysis software. Error bars represent standard deviation observed among ~100 analyzed colonies. **c**, Activity of *pGAL1* in xylose-containing media. Strains containing a fluorescent reporter driven by *pGAL1* were cultured in the appropriate medium for several days, then re-seeded in a different carbon source overnight before measurement of fluorescence. Growth test of *xylA* (**d**) and *xylA3** (**e**) pathway mutants. Indicated strains were inoculated into 1 mL of xylose-containing minimal media at a starting optical density of 0.01 and optical density was measured over the course of 3 days. **f**, Growth test of *spt15* mutants in 1.2% 1-butanol, **g**, 1.3% 1-butanol, **h**, 1.4% 1-butanol, **i**, 1.5% 1-butanol. For (**a**), error bars represent standard deviation of technical triplicates. For (**b**-**i**), error bars represent the standard deviation of biological triplicates.

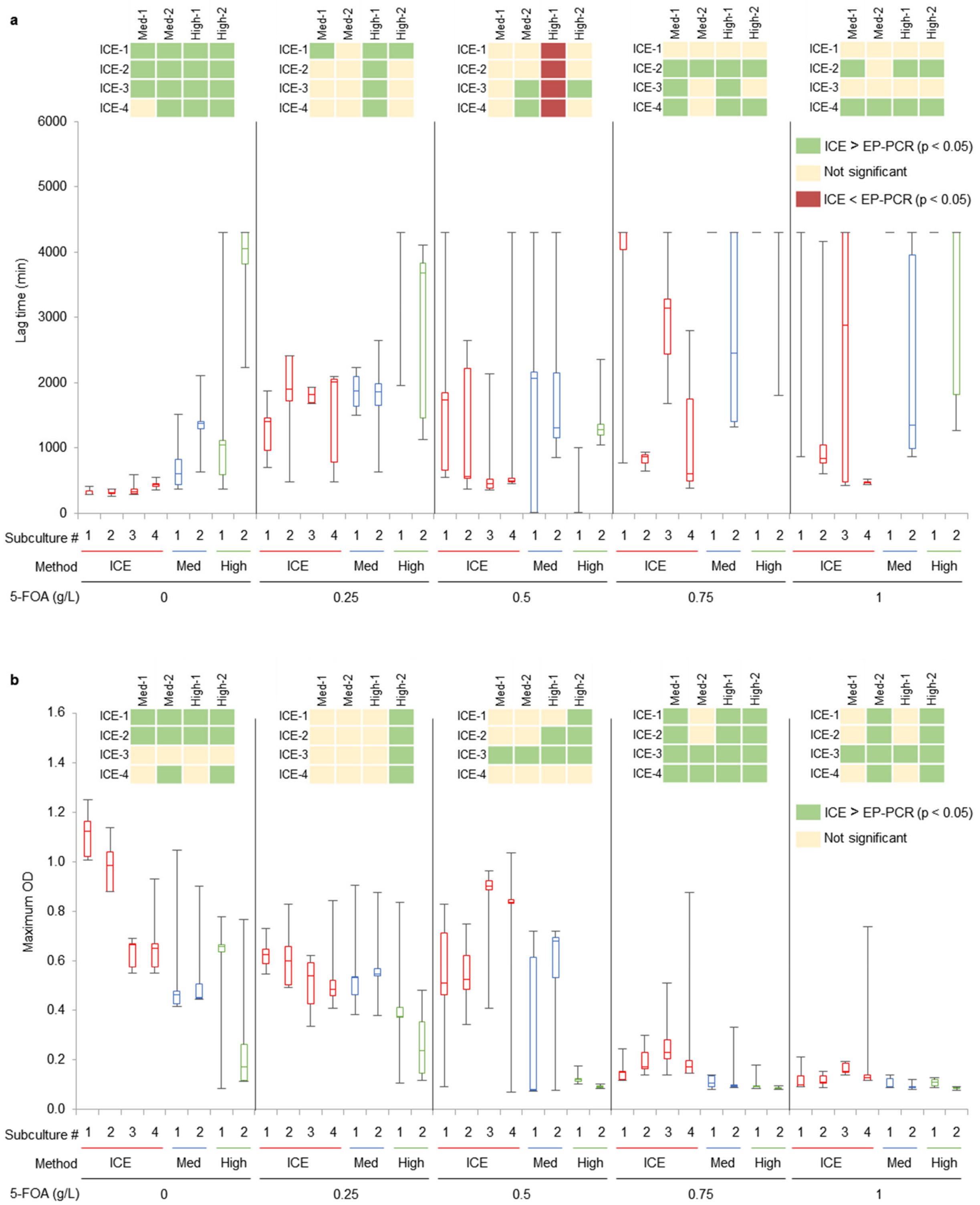

Supplementary Figure 4 | Comparison of Lag time and Max OD of ICE-derived and EP-PCR-derived mutants. a, Lag time. **b**, Max OD. Lines on boxplots represent, from top to

bottom, maximum observed value, 75th percentile, median, 25th percentile, and minimum observed value. P-values used to generate heatmaps were computed using a Mann-Whitney U-test.

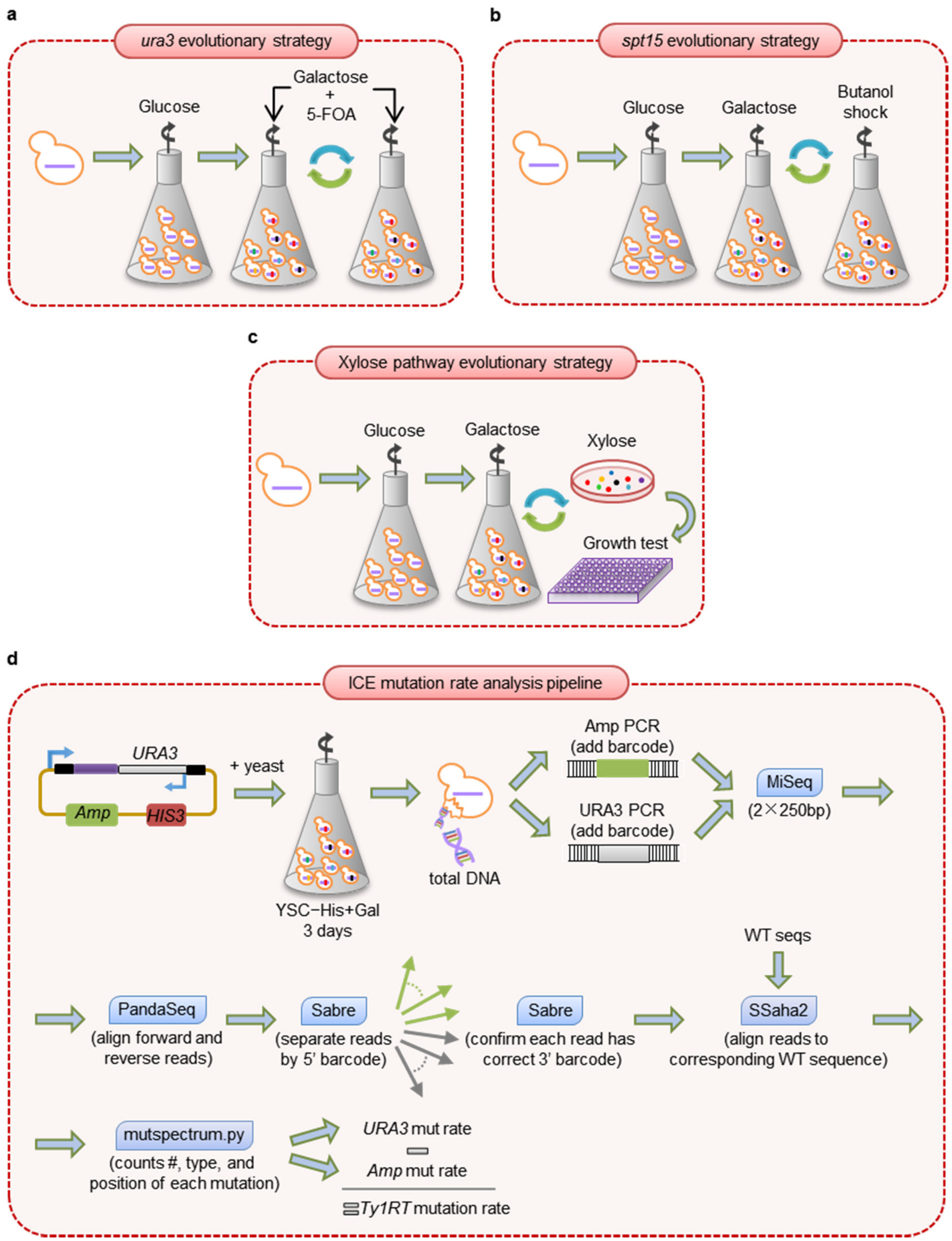

Supplementary Figure 5 | Selection methods employed during evolution of genetic cargos in this study and sequencing analysis workflow. **a**) Cells with *URA3*-containing retroelements were precultured in glucose and transferred to galactose-containing media for three days to induce mutagenesis. Induced cells were exposed to increasing concentrations of 5-FOA in

uracil-deficient, galactose containing media in order to continuously generate and select for *ura3* variants maintaining uracil biosynthesis activity while reducing activity on 5-FOA. After several serial transfers, single isolates were selected. Beneficial *ura3* mutants were transferred into a fresh Ty1/yeast genomic background and the induction/selection procedures were repeated during a second round. **b**) Cells with *SPT15*-containing retroelements were precultured in glucose and transferred to galactose-containing media for three days to induce mutagenesis. Induced cells were exposed to a short "shock" of high 1-butanol concentration to deplete detrimental variants, and single isolates were selected. Beneficial *spt15* mutants were transferred into a fresh Ty1/yeast genomic background and the induction/selection procedures were repeated during a second round. **c**) Cells with xylose pathway-containing retroelements were precultured in glucose and transferred to galactose-containing media for three days to induce mutagenesis. Induced cells were plated on xylose- containing agar plates in order to continuously generate and select for pathway variants with increased activity. Single isolates were selected by choosing the largest yeast colonies and subsequently assayed for increased growth in liquid culture. **d**) Computational workflow for analysis of Ty1 mutation rates through next-generation sequencing. All computer programs are available upon request.

Supplementary Tables S1 to S14

Supplementary Table 1: Mutations identified at position 135 in d*KanMX* reversion mutants.

Codon	# recovered
**TGA (stop codon in d*KanMX*)**	6
**TGG (Trp)**	17
**TGT (Cys, present in WT *KanMX*)**	7
**TCA (Ser)**	6
**AGA (Arg)**	4
**CGA (Arg)**	4
**GGA (Gly)**	3
**TGC (Cys)**	1
**TTA (Leu)**	1
**Total**	**49**

Supplementary Table 2: Comparison of ICE to other mutagenesis techniques**.**

Method	Mutation Rate (kb^{-1})	Total mutants generated ($round^{-1}$ $cell^{-1}$)	Distinct mutants generated (per round per 10^{10} cells transformed)			Localization of mutants	Mutagenesis occurs	Continuous diversity generation?	Effort per round	Notes
			100bp	1000bp	5000bp					
**ICE**	0.15	$3.7*10^{-2}$ [a]	$2.7*10^{4}$ [b]	$2.7*10^{6}$ [b]	$1.6*10^{7}$ [c]	Genome-wide or along defined cargo[d]	*in vivo*	Rounds can be continuous or discrete	Cargo integrated into genome, followed by cell outgrowth.	
**EP-PCR**	0-16	10^{-2} [e]	$1.2*10^{7}$ [fgh]	$9.5*10^{7}$ [fg]	$9.5*10^{7}$ [fg]	Along defined cargo	*in vitro*	No	Cargo is mutated using epPCR, cloned into an expression vector, amplified in *E. coli*, and transformed into yeast. [i]	[j]
**Orthogonal DNA polymerase** [1]	$4*10^{-5}$	1	$3*10^{2}$ [b]	$3*10^{3}$ [b]	$1.5*10^{4}$ [b]	Along defined cargo	*in vivo*	Yes	Cargo is integrated into plasmid, followed by cell outgrowth.	[k]
**Random drift** [2]	$2*10^{-7}$	1	$1.5*10^{2}$ [b]	$1.5*10^{3}$ [b]	$7.3*10^{3}$ [b]	Genome-wide	*in vivo*	Yes	Cell outgrowth.	
**YOGE** [3]	user-defined	$2*10^{-4}$	$1.9*10^{6}$ [l]			Site-directed	*in vivo* from library generated *in vitro*	Continual recombination of existing libraries [m]	Two independent libraries generated through epPCR or oligo synthesis, cloned into *E. coli*, and then combined using YOGE through multiple yeast transformations. [in]	[o]
**CRISPR-Cas9** [4]	user-defined	$7.5*10^{-5}$ [p], $2.2*10^{-5}$ [q], or 10^{-2} [r]	$7.1*10^{5}$ [lp], $2.1*10^{5}$ [lq], or $9.5*10^{7}$ [lr]			Site-directed	*in vivo* from library generated *in vitro*	Continual recombination of existing libraries [m]	Two independent libraries generated through epPCR or oligo synthesis, cloned into *E. coli*, and then combined using CRISPR/Cas9 through multiple yeast transformations. [in]	[s]
**Mating/ Recom-bination** [5]	user-defined	$6*10^{-2}$	$5.7*10^{8}$ [lt]			Site-directed	*in vivo* from library generated *in vitro*	Continual recombination of existing libraries [m]	Two independent libraries generated through epPCR or oligo synthesis, followed by cloning into *E. coli* and transformation into yeast. These two *in vivo* libraries are then combined through mating. [i]	

a) Fig. 2b.

b) This number was calculated using PEDEL[6] with a "Library size" of 10^{10} x (number of total mutants generated) and a "mean number of point mutations per sequence" of 0.15 x (cargo length). It is important to note that the number of distinct amino acid variants will be much lower than these values for random mutant libraries, ranging from 20-50%, depending on the nucleotide sequence being mutated[7]. For libraries incorporating synthetic oligonucleotides, such as the "site-directed" methods above, this efficiency can approach 100%.

c) Number of total mutants generated for a 5kb cargo has been calculated using a library size of $9.49*10^{-3}$ per cell (Supplementary Figure 2a).

d) Genome-wide mutagenesis occurs during continuous mutagenesis/selection as Ty1 integrates into alternate loci in the genome.

e) This is the maximum yeast plasmid transformation efficiency [8].

f) Consider M cells being transformed with a DNA library of size L. The probability that a particular member of L is delivered to a particular member of M is 1/L. The probability that this member of L is not delivered to this member of M is 1-(1/L). The probability that this member of L is not delivered to any member of M is $(1-(1/L))^M$. Thus, the probability that this member of L is delivered to at least one member of M is $1-(1-(1/L))^M$. Summing over all members of L, the expected value of the number of members of L which were delivered at least once to the cells in M is given by $L(1-(1-(1/L))^M)$. This calculation assumes that the DNA molecules composing L are numerous enough that successful transformation events do not significantly change the proportion of each member in L. This calculation also assumes that each member of L is at the same proportion. This is not true for error-prone-PCR-derived libraries since sequences with fewer mutations likely outnumber those with larger numbers of mutations. This bias will reduce the number of distinct mutants generated beyond the simple formula presented here.

g) For error-prone PCR, a 10^{-2} transformation efficiency implies 10^8 library-containing cells, so M for these cells is 10^8.

h) $1.2*10^7$ is the maximum number of distinct variants of a 100bp sequence which can be generated through error-prone PCR using a 16 kb^{-1} mutation rate, and so for this cell L was assumed to be $1.2*10^7$.

i) Most methods for diversity generation have been benchmarked for cell populations $\sim10^8$ in size. Effort has been scaled accordingly to bring these methods into line with the scale of ICE.

j) For each template size we have assumed a mutation rate of 16 kb^{-1} to generate an upper bound for the number of distinct mutants attainable by error-prone PCR. Although the required library size L is different for every experiment, and has been reported exceeding 10^{12} for *E. coli* [9], we have chosen 10^9 here as this is the minimum value enabling ~95% of transformed yeast cells to contain unique library members. Percentages approaching 100% are attained with severely diminishing returns.

k) Authors note a requirement for the mutated gene to be expressed from cytoplasmic RNAP with low expression capacity.

l) Consider M cells which already contain a library of size L1 (such that the number of cells which contain each member of L1 is M/L1), and which are being transformed with a different library of size L2. The probability that a particular member of L2 is delivered to a particular member of M is 1/L2. The probability that this member of L2 is not delivered to this member of M is 1-(1/L2). The probability that this member of L2 is not delivered to any member of M containing a particular member of L1 is $(1-(1/L2))^{M/L1}$. Thus, the probability that this member of L2 is delivered to at least member of M containing a particular member of L1 is $1-(1-(1/L2))^{M/L1}$. Summing over all members of L1 and L2, the expected value of the number of unique L1-L2 combinations is $L1*L2*(1-(1-(1/L2))^{M/L1})$. This represents the best-case scenario of no biases in library representation among L1 or L2 libraries. The general form of this expression is (sum(i=1 to L1) of $L2*(1-(1-(1/L2))^{Mi})$ where Mi is # cells transformed with each member of L1. The number of distinct mutants generated will be lower if, for example, there are a large population of WT cells in L1.

m) In our opinion, the major benefit of these approaches over methods such as error-prone PCR for random mutagenesis of genes and pathways is the ability to explore combinations of two distinct libraries, so that's the mode that is highlighted here. YOGE and CRISPR-Cas9 can also be used for generation of mutations throughout the yeast genome, but as this is a different goal than that of ICE, it is not considered in detail here.

n) This is the effort required to generate one library from 10^{10} total cells. Additional effort is required to combine multiple libraries.

o) "Number of total mutants generated" is a combination of 1% electoporation survival rate and 2% recombination frequency in survivors [3]. We have chosen two libraries of size 4400, as these are the minimum values enabling ~95% of transformed yeast cells to contain unique library members. To accomplish this task using a single library, the library would need to contain $1.9*10^7$ unique members in order for $1.9*10^6$ to make it into yeast.

p) 90bp dsoligo repair template

q) 1.4kb repair template

r) Plasmid repair template, yeast plasmid transformation frequency

s) Library size for plasmid repair template assumes pre-transformation of Cas9 and repair plasmid, followed by transformation of sgRNA plasmid using the LiAc/ssDNA method, as this is the method which results in the largest theoretical library size. If instead one wishes to generate the same diversity using a single library (instead of combining multiple libraries), this library must be at least $7.2*10^6$ (90bp dsoligo repair template), $2.1*10^6$ (1.4kb repair template), or $9.6*10^8$ (plasmid repair template).

t) This has been calculated assuming L2 is # distinct variants in donor cells

Supplementary Table 3: Enzyme kinetics and sequence analysis of XylA and XylA3* pathway mutants.

Mutant	Parent	ICE performance (rounds and cycles)	Selection condition	Growth rate (h^{-1})	V_{max} (µmol min^{-1} mg $protein^{-1}$)	K_m (mM)	Mutations
***I3K*-66**	*XylA3** pathway	one round and continuous cycles	YP + 20 g L^{-1} Xylose plate	0.140	0.126 ± 0.008	90.03 ± 14.40	$Ile^{433}Val$ (in XylA3*)
***I3K*-20**	*XylA3** pathway	one round and continuous cycles	YSC −His + 20 g L^{-1} Xylose plate	0.134	0.134 ± 0.003	109.45 ± 1.33	$Ala^{48}Ser$, $Ile^{433}Val$, $Met^{435}Ile$ (in XylA3*)
***IK*-34**	*XylA* pathway	one round and continuous cycles	YSC −His + 20 g L^{-1} Xylose plate	0.136	-	-	$Glu^{164}Lys$ (in XKS1)
***XylA3** pathway Wild type**		-	-	0.106	0.118 ± 0.007	52.06 ± 16.47	-
***XylA* pathway Wild type**		-	-	0.079	-	-	-

Supplementary Table 4: Media Recipes Used in this Study.

Medium	Ingredients	Supplier
Lysogeny Broth (LB)	10 g L^{-1} tryptone, 5 g L^{-1} yeast extract, 10 g L^{-1} sodium chloride	Teknova
LB Agar	LB, 15 g L^{-1} agar	This work
LB + ampicillin	LB + 100 µg mL^{-1} ampicillin	This work
LB + ampicillin Agar	LB + ampicillin, 15 g L^{-1} agar	This work
Yeast Extract, Peptone, Dextrose Broth (YPD)	10 g L^{-1} yeast extract, 20 g L^{-1} peptone, 20 g L^{-1} glucose.	This work
YPD + G418	YPD, 0.4g L^{-1} G418	This work
YPD Agar	YPD, 15 g L^{-1} agar	This work
YPD Agar + G418	YPD Agar, 0.4 g L^{-1} G418	This work
Yeast Extract, Peptone, Galactose Broth (YPG)	10 g L^{-1} yeast extract, 20 g L^{-1} peptone, 20 g L^{-1} galactose.	This work
YPG Agar	YPG, 15 g L^{-1} agar	This work
Yeast Extract, Peptone, Xylose Broth (YPX)	10 g L^{-1} yeast extract, 20 g L^{-1} peptone, 20 g L^{-1} xylose.	This work
YPX Agar	YPX, 15 g L^{-1} agar	This work
Yeast Synthetic Complete Media (YSC)	6.7 g L^{-1} yeast nitrogen base, 20 g L^{-1} glucose, 0.79 g L^{-1} CSM	This work
YSC Gal	6.7 g L^{-1} yeast nitrogen base, 20 g L^{-1} galactose, 0.79 g L^{-1} CSM	This work
YSC Xyl	6.7 g L^{-1} yeast nitrogen base, 20 g L^{-1} xylose, 0.79 g L^{-1} CSM	This work
YSC Agar	YSC, 15 g L^{-1} agar	This work
YSC Gal Agar	YSC Gal, 15 g L^{-1} agar	This work
YSC Xyl	YSC Xyl, 15 g L^{-1} agar	This work
YSC −His	6.7 g L^{-1} yeast nitrogen base, 20 g L^{-1} glucose, 0.77 g L^{-1} CSM −His	This work
YSC Gal −His	6.7 g L^{-1} yeast nitrogen base, 20 g L^{-1} galactose, 0.77 g L^{-1} CSM −His	This work
YSC Xyl −His	6.7 g L^{-1} yeast nitrogen base, 20 g L^{-1} xylose, 0.77 g L^{-1} CSM −His	This work
YSC –His Agar	YSC −His, 15 g L^{-1} agar	This work
YSC Gal –His Agar	YSC Gal −His, 15 g L^{-1} agar	This work
YSC Xyl –His Agar	YSC Xyl −His, 15 g L^{-1} agar	This work
YSC −His −Trp	6.7 g L^{-1} yeast nitrogen base, 20 g L^{-1} glucose, 0.72 g L^{-1} CSM −His −Trp	This work
YSC Gal −His −Trp	6.7 g L^{-1} yeast nitrogen base, 20 g L^{-1} galactose, 0.72 g L^{-1} CSM −His −Trp	This work
YSC Xyl −His −Trp	6.7 g L^{-1} yeast nitrogen base, 20 g L^{-1} xylose, 0.72 g L^{-1} CSM −His −Trp	This work
YSC −His −Trp Agar	YSC −His −Trp, 15 g L^{-1} agar	This work
YSC Gal −His −Trp Agar	YSC Gal −His −Trp, 15 g L^{-1} agar	This work
YSC Xyl −His −Trp Agar	YSC Xyl −His −Trp, 15 g L^{-1} agar	This work
YSC −Trp	6.7 g L^{-1} yeast nitrogen base, 20 g L^{-1} glucose, 0.74 g L^{-1} CSM −Trp	This work
YSC Gal −Trp	6.7 g L^{-1} yeast nitrogen base, 20 g L^{-1} galactose, 0.74 g L^{-1} CSM −Trp	This work
YSC Xyl −Trp	6.7 g L^{-1} yeast nitrogen base, 20 g L^{-1} xylose, 0.74 g L^{-1} CSM −Trp	This work
YSC −Trp Agar	YSC −Trp, 15 g L^{-1} agar	This work
YSC Gal −Trp Agar	YSC Gal −Trp, 15 g L^{-1} agar	This work
YSC Xyl −Trp Agar	YSC Xyl −Trp, 15 g L^{-1} agar	This work

Agar	Agar	Teknova
Ampicillin	Ampicillin	Sigma
G418	Geneticin	Gold Biotechnology
Yeast extract	Yeast extract	Amresco
Peptone	Peptone	Amresco
Glucose	Glucose	MP Biomedicals
Galactose	Galactose	Sigma Aldrich
Xylose	Xylose	Acros
Yeast nitrogen base	(for powder): 5 g ammonium sulfate, 2 µg biotin, 400 µg calcium pantothenate, 2 µg folic acid, 2 mg inositol, 400 µg niacin, 200 µg p-aminobenzoic acid, 400 µg pyridoxine hydrochloride, 200 µg riboflavin, 400 µg thiamin hydrochloride, 500 µg boric acid, 40 µg copper sulfate, 100 µg potassium iodide, 200 µg ferric chloride, 400 µg manganese sulfate, 200 µg sodium molybdate, 400 µg zinc sulfate, 1 g monopotassium phosphate, 0.5 g magnesium sulfate, 0.1 g sodium chloride, 0.1 g calcium chloride.	Difco
CSM	(for powder): 10 mg adenine, 50 mg L-arginine, 80 mg L-aspartic acid, 20 mg L-histidine, 50 mg L-isoleucine, 100mg L-leucine, 50 mg L-lysine, 20 mg L-methionine, 50 mg L-phenylalanine, 100mg L-threonine, 50 mg L-tryptophan, 50 mg L-tyrosine, 20 mg uracil, 140 mg L-valine.	Sunrise Science Products / MP Biomedicals
CSM −His	(for powder): 10 mg adenine, 50 mg L-arginine, 80 mg L-aspartic acid, 50 mg L-isoleucine, 100mg L-leucine, 50 mg L-lysine, 20 mg L-methionine, 50 mg L-phenylalanine, 100mg L-threonine, 50 mg L-tryptophan, 50 mg L-tyrosine, 20 mg uracil, 140 mg L-valine.	Sunrise Science Products / MP Biomedicals
CSM −His −Trp	(for powder): 10 mg adenine, 50 mg L-arginine, 80 mg L-aspartic acid, 50 mg L-isoleucine, 100mg L-leucine, 50 mg L-lysine, 20 mg L-methionine, 50 mg L-phenylalanine, 100mg L-threonine, 50 mg L-tyrosine, 20 mg uracil, 140 mg L-valine.	Sunrise Science Products / MP Biomedicals
CSM −Trp	(for powder): 10 mg adenine, 50 mg L-arginine, 80 mg L-aspartic acid, 20 mg L-histidine, 50 mg L-isoleucine, 100mg L-leucine, 50 mg L-lysine, 20 mg L-methionine, 50 mg L-phenylalanine, 100mg L-threonine, 50 mg L-tyrosine, 20 mg uracil, 140 mg L-valine.	Sunrise Science Products / MP Biomedicals

Supplementary Table 5: Plasmids obtained for this study.

Plasmid Name	Source
**p413-*pTEF1***	[10]
**p423-*pTDH3***	[10]
**p414-*pCYC1***	[10]
**p414-*pTDH3***	[10]
**p424-*pTEF1***	[10]
**p424-*pTDH3***	[10]
**p415-*pTEF1***	[10]
**p425-*pTDH3***	[10]
**p416-*pCYC1***	[10]
**p416-*pTEF1***	[10]
**p416-*pTDH3***	[10]
**p42K**	[11]
**pUG6**	[12]
**pSH47**	[13]
**p416-*pCYC1-YFP***	[14]
**p416-*pTEF1-YFP***	[14]
**p416-*pTDH3-YFP***	[14]
**hCas9**	[4]
**p416-*pTDH3-eGFP***	[14]
**p416-*pTEF1-LacZ***	[14]
**p416- *pTDH3-XylA***	[15]
**p416- *pTDH3-XylA3****	[15]
**pUAStef-UAScit-UASclb**	[16]
**pmStrawberry**	Clontech
**p426-*pTEF1***	[10]

Supplementary Table 6: Strains obtained for this study.

Strain	Genotype	Source
S. cerevisiae **BY4741**	*MATa; his3Δ1; leu2Δ0; met15Δ0; ura3Δ0*	EUROSCARF
S. cerevisiae **CEN.PK2**	*MATa; ura3-52; trp1-289; leu2-3,112; his3Δ1; MAL2-8*^{C}*; SUC2*	EUROSCARF
S. cerevisiae **S288C**	*MATα SUC2 gal2 mal2 mel flo1 flo8-1 hap1 ho bio1 bio6*	ATCC
S. cerevisiae **YSX3**	*MATα trp1-112 leu2*::*LEU2-PsXYL1 ura3*::*URA3-PsXYL2 Ty3*::*NEO-PsXYL3*	[17]
S. cerevisiae **BY4741 Δ*rrm3***	*MATa; his3Δ1; leu2Δ0; met15Δ0; ura3Δ0 rrm3::KanMX*	Yeast Haploid Knockout Collection
S. cerevisiae **BY4741 Δ*ice2***	*MATa; his3Δ1; leu2Δ0; met15Δ0; ura3Δ0 ice2::KanMX*	Yeast Haploid Knockout Collection
S. cerevisiae **BY4741 Δ*ckb2***	*MATa; his3Δ1; leu2Δ0; met15Δ0; ura3Δ0 ckb2::KanMX*	Yeast Haploid Knockout Collection
S. cerevisiae **BY4741 Δ*hir3***	*MATa; his3Δ1; leu2Δ0; met15Δ0; ura3Δ0 hir3::KanMX*	Yeast Haploid Knockout Collection
S. cerevisiae **BY4741 Δ*apl2***	*MATa; his3Δ1; leu2Δ0; met15Δ0; ura3Δ0 apl2::KanMX*	Yeast Haploid Knockout Collection
S. cerevisiae **BY4741 Δ*mrc1***	*MATa; his3Δ1; leu2Δ0; met15Δ0; ura3Δ0 mrc1::KanMX*	Yeast Haploid Knockout Collection
S. cerevisiae **BY4741 Δ*cac2***	*MATa; his3Δ1; leu2Δ0; met15Δ0; ura3Δ0 cac2::KanMX*	Yeast Haploid Knockout Collection
S. cerevisiae **BY4741 Δ*mre11***	*MATa; his3Δ1; leu2Δ0; met15Δ0; ura3Δ0 mre11::KanMX*	Yeast Haploid Knockout Collection
S. cerevisiae **BY4741 Δ*cac3***	*MATa; his3Δ1; leu2Δ0; met15Δ0; ura3Δ0 cac3::KanMX*	Yeast Haploid Knockout Collection
K. lactis **CBS 2359**	*MAT**a***	ATCC (8585)

Supplementary Table 7: Oligonucleotides used in this study (IDT).

Primer Name	Sequence
**GalCas9F**	AAAAGTATCAACAAAAAATTGTTAATATACCTCTATACTTTAACGTCAAGGAGAAAAAACCCCGGATATGGACAAGAAGTACTCCATTGG
**CYC1tCas9R**	AGGGCGTGAATGTAAGCGTGACATAACTAATTACATGATCACACCTTCCTCTTCTTCTTG
**Cas9CYC1tF**	AGGGCTGACCCCAAGAAGAAGAGGAAGGTGTGATCATGTAATTAGTTATGTCACGCTTAC
**gRNACYC1tR**	AAGTGGCACCGAGTCGGTGGTGCTTTTTTTGTTTTTTATGTCTCGAGCGTCCCAAAACCT
**gRNAF**	TGTACGCATGTAACATTATACTGAAA
**gRNAR**	AAAACGACGGCCAGTGAG
**PacIP416F**	/5PHOS/GG-TTAATTAA-GGTACCCAATTCGCCCT
**NotICYCtR**	/5PHOS/GCGGCCGC-CGAGCGTCCCAAAACCT
**NotIgRNAF**	GG-GCGGCCGC-AGACATAAAAAACAAAAAAAGCACCA
**PacIgRNAR**	GG-TTAATTAA-CTTTGAAAAGATAATGTATGATTATGCTTTCA
**SacIGalF**	CCCCCC-GAGCTC-ACGGATTAGAAGCCGCC
**SpeXbaIGalR**	GG-ACTAGT-TCTAGA-GGTTTTTTCTCCTTGACGTTAAAG
**GalpF1**	CTCACTAAAGGGAACAAAAGCTGGAGCTCCTAGTACGGATTAGAAGCCGC
**Gal1pFixR2**	TATACTAGAAGTTCTCCTCGAGGCGGTAGAGGAATAAGAAGTAATACAAACCGAAAATGT
**GalpF2**	CAGCTATGACCATGATTACGCCAAGCGCGCAATTAACCCTCACTAAAGGGAACAAAAGCT
**GalpR2**	TTCCATTGTTGATAAAGGCTATAATATTAGGTATACAGAATATACTAGAAGTTCTCCTCG
**TyH3GenomeF**	GCGCGGGCAAAGCCCAAAAG
**TyH3GenomeR**	TGCGCAAGCCCGGAATCGAA
**TyH3PCRF1**	TATCAACAATGGAATCCCAACAATTATCTCAACATTCACCCAATTCTCATGGTAGCGCCT
**TyH3PCRR1**	GAACGGTTTCAATTGGAGAAATTGGAACAGCCTTCAAAGCTGCAATCAGGTGAATTCGTT
**TyH3PCRF2**	CTTCTAGTATATTCTGTATACCTAATATTATAGCCTTTATCAACAATGGAATCCCAACAA
**TyH3PCRR2**	ACTTTTGGACCATCCATACCTGGTTTCAACTTAACTGGAACGGTTTCAATTGGAGAAATT
**TyH3PCRF3**	CTCGAGGAGAACTTCTAGTATATTCTGTATACCTAATATTATAGCC
**TyH3FlankR**	ACTTTTGGACCATCCATACCT
**HIVnoATGF**	GCTTTGAAGGCTGTTCCA
**HIVRThomR**	ACCGATTATTTAAAGCTGCAGCCCAAGCTTATCGATTTACAAGATCTTTCTAATACCGGC
**URA3F**	TTGTAAATCGATAAGCTTGGGCTGCAGCTTTAAATAATCGGTGTTAGTTTTGCTGGCCGC
**URA3AIR**	GCAGAAAAGCCTCCTTTAGTCCATATTAACATACCCGCGATGAAGGTTACGATTGGTTGA

**URA3R**	AAAAAATGAGCAGGCAAGATAAACGAAGGCAAAGATGTCGAA AGCTACATATAAGGAACG
**URA3AIF**	CTCGAATTTTTACTAACAAATGGTATTATTTATAACAGCCGCCC ATGTCTCTTTGAGCAA
**AInoass2F**	TCTAGAGGATCCCCGGGTACCGAGCTCGAATTTTTACTAACAA ATGGTATTATTTATAAC
**AInoass3F**	GTATGTTAATATGGACTAAAGGAGGCTTTTCTGCAGGTCGACT CTAGAGGATCCCCGGG
**LTRF1**	TGTACTAGAGGATCTATTACATTATGGGTGGTATGTTGGAATA GAAATCAACTATCATCT
**LTRR1**	CGCGCGTAATACGACTCACTATAGGGCGAATTGGGTACCTGAG AAATGGGTGAATGTTGA
**PPTNotIF**	GCGGCCGCGATCTATTACATTATGGGTGGTATGT
**LTRflankR**	AGGGTTTTCCCAGTCACG
**HISpromF**	CTTTGCCTTCGTTTATCTTGCC
**HISpromR**	ACCACCCATAATGTAATAGATCGCGGCCGCCTCTAGTACACTC TATATTTTTTTATGCCT
**P416F**	GGTACCCAATTCGCCCT
**P416R**	GAGCTCCAGCTTTTGTTCC
**Ty1RTHDF1**	CCGTTCACCTTCAATCGATGCTTCTCCACCGGAAAATAATTCAT CGCACAATATTGTTCC
**Ty1RTHDR1**	AAAGCTGCAGCCCAAGCTTATCGATCTAATGAATCCATTTGTTA GTTAATAGTTTAAATG
**Ty1RTHDF2**	CGCAGATAAGTGACCAAGAGACTGAGAAAAGGATTATACACC GTTCACCTTCAATCGATG
**Ty1RTHDR2**	CATATTTGAGAAGATGCGGCCAGCAAAACTAACACCGATTATT TAAAGCTGCAGCCCAAG
**RTmutF**	TCAACAGTAAGAAAAGATCATTAGAAGA
**RTmutR**	GGAAGGGATGCTAAGGTAGAG
**HistermR**	CACCGATTATTTAAAGCTGCAGCCC
**LTRTEFF2**	TACCACCCATAATGTAATAGATCGCGGCCGC-ATAGCTTCAAAATGTTTCTACTCCTTTT
**URA3TEFR2**	GCACGTTCCTTATATGTAGCTTTCGACAT-AAACTTAGATTAGATTGCTATGCTTTCTTT
**RT-URA3-BBf**	ATAAGCTTGGGCTGCAGC
**RT-URA3-BBr**	CGATCTAATGAATCCATTTG
**RT-eGFPf**	CATTTAAACTATTAACTAACAAATGGATTCATTAG-TGCGTAAAGGAGAAGAACTTTTCAC
**RT-eGFPr**	CCGATTATTTAAAGCTGCAGCCCAAGCTTATCGAT-TTAAACTGCTGCAGCGTAG
**RT-LacZf**	CATTTAAACTATTAACTAACAAATGGATTCATTAG-TGACCATGATTACGGATTCAC
**RT-LacZr**	CCGATTATTTAAAGCTGCAGCCCAAGCTTATCGAT-TTATTTTTGACACCAGACCAACTGG
**RT-CAN1f**	CATTTAAACTATTAACTAACAAATGGATTCATTAG-TGACAAATTCAAAAGAAGACGC
**RT-CAN1r**	CCGATTATTTAAAGCTGCAGCCCAAGCTTATCGAT-CTATGCTACAACATTCCAAA
**Lacz-URA3-BBf**	TAAATCGATAAGCTTGGGCTGC

**Lacz-URA3-BBr**	TTGACACCAGACCAACTGG
**LacZ-eGFPf**	CATTACCAGTTGGTCTGGTGTCAAAAATAA-TGCGTAAAGGAGAAGAACTTTTCAC
**LTRCYCF2**	CTATTCCAACATACCACCCATAATGTAATAGATCGCGGCCGC-ATTTGGCGAGCGTTGGT
**URA3CYCR2**	GTAGCAGCACGTTCCTTATATGTAGCTTTCGACAT-TTAGTGTGTGTATTTGTGTTTGCG
**LTRGPDF2**	ACCACCCATAATGTAATAGATCGCGGCCGC-AGTTTATCATTATCAATACTCGCCATTTC
**URA3GPDR2**	GAGTAGCAGCACGTTCCTTATATGTAGCTTTCGACAT-ATCCGTCGAAACTAAGTTCTGG
**PPTNotIF**	GCGGCCGC-GATCTATTACATTATGGGTGGTATGT
**reGREsptR**	TAACTAACAAATGGATTCATTAGATCGATAAGCTTGGGCTGCAGCTTTAAATAATCGGTG-TCACATTTTTCTAAATTCACTTAGCACAGG
**reGREsptF**	TGTTATAAATAATACCATTTGTTAGTAAAAATTCGGCGCGCCAAAAGCCTCCTTTAGTCCATATTAACATAC-TGGCCGATGAGGAACGTT
**reGREtefF**	GATGATAGTTGATTTCTATTCCAACATACCACCCATAATGTAATAGATCGCGGCCGC-ATAGCTTCAAAATGTTTCTACTCCTTTTTTACT
**reGREtefR**	CTAAAGGAGGCTTTTGGCGCGCCGAATTTTTACTAACAAATGGTATTATTTATAACAG-TAAACTTAGATTAGATTGCTATGCTTTCTTTC
**Intron*ura3(3-5)*F**	ATATGGACTAAAGGAGGCTTTTGGCGCGCCGAATTTTTACTAACAAATGGTATTATTTATAACAG-GCCTATAGGTTCCTTTGTTACTTCT
**LTRTEFF2**	TACCACCCATAATGTAATAGATCGCGGCCGC-ATAGCTTCAAAATGTTTCTACTCCTTTT
**Ty1URA3F**	CATTTAAACTATTAACTAACAAATGGATTCATTAGATCGATAAGCTTGGGCTGCAGCTTTAAATAATCGGTG-TTAGTTTTGCTGGCCGCA
**Intron*ura3(3-5)*R**	ATAATACCATTTGTTAGTAAAAATTCGGCGCGCCAAAAGCCTCCTTTAGTCCATATTAACATAC-CTTTTGATGTTAGCAGAATTGTCATG
**(m)GAXnewintronF**	CATCCCTGCGTCTACAGC
**(m)GAXnewintronR**	TGTAAATCTATTTCTTAAACTTCTTAAATTCTACTTTTATAGTTAGTCTTTTTTTTAGTTTTAAAACA-CCAGAACTTAGTTTCGACGGAT
**(m)GA3XnewintronXylA3for**	TGGTAGCACCAGAACCTTGATAC
**TART-FullMCSF**	CC-TTAATTAACCGCGGGGATCCCCCGGGCGGCCGCGATCTATTAC
**TART-FullMCSR**	CC-TTAATTAACCTGCAGGGAATTCCTAATGAATCCATTTGTTAGTTAATAGTTTAAATGT
**NotITEFF**	ATAAGAAT-GCGGCCGC-ATAGCTTCAAAATGTTTCTACTCCTT
**XmaITEFR**	TCCC-CCCGGG-AAACTTAGATTAGATTGCTATGCTTTCT
**SacIIXylAR**	C-CCGCGG-TTATTGATACATCGCGACAATAGCC
**XmaIXylAF**	TCCC-CCCGGG-ATGGCTAAAGAATATTTCCCTCAAA
**PacIXKS1F**	CC-TTAATTAA-ATGTTGTGTTCAGTAATTCAGAGAC

**EcoRIXKSR**	G-GAATTC-TTAGATGAGAGTCTTTTCCAGTTCG
**P2AXKS1F**	/5PHOS/CAAGATGGTGATGTTGAAGAAAATCCAGGACCA-TTGTGTTCAGTAATTCAGAGACAGA
**P2AXylAR**	/5PHOS/TTTTAATAAAGAAAAGTTGGTAGCACCAGAACC-TTGATACATCGCGACAATAGCC
**XylAMCSintronF**	GCGCCGAATTTTTACTAACAAATGGTATTATTTATAACAGT-CCCGGGAAACTTAGATTAGATTGC
**XylAMCSintronR**	GCCAAAAGCCTCCTTTAGTCCATATTAACATACTGGCTAAAGA ATATTTCCCTCAAATTC
**XmaIGPDR**	TCCC-CCCGGG-ATCCGTCGAAACTAAGTTCTGG
**NotImegaGPDF**	ATAAGAAT-GCGGCCGC-ATAGCTTCAAAATGTTTCTACTCCTT
**(m)GA3XnewintronRTfor**	ACCAAGAAGAACATTGCTGATGTG
**(m)GA3XnewintronP2Arev**	TGAATATGGAAAGAAGAATGGAGAACCGAAACAAACCTCAGG AAAACAAGAACTATACGAGGCTATTA-TCGCGATGTATCAAGGTTCTGG
**XmaIXylA3F**	TCCC-CCCGGG-ATGGCTAAAGAATATTTCCCTCAAA
**XylAGPDnointronF**	TTTGAATTTGAGGGAAATATTCTTTAGCCATATCCGTCGAAACT AAGTTCTGGTGTTTTA
**XylAGPDnointronR**	TAAAACACCAGAACTTAGTTTCGACGGATATGGCTAAAGAATA TTTCCCTCAAATTCAAA
**GREnewF**	TTTCACAGGTAGTTCTGGTCCATTGGTGAAAGTTTGCGGCTTGC AGAGCACAGAGGCCGCAGAATGT-GAACAAAAGCTGGAGCTCCTAGT
**GREnewR**	ACGAAATTTGCTATTTGTTAGAGTCTTTTACACCATTTGTCTCC ACACCTCCGCTTACATCAACACCA-CTTGGCCTCCTCTAGTACACT
**XbaIXKS1**	CCCCCC-TCTAGA-ATGTTGTGTTCAGTAATTCAGAGA
**XKS1XhoI**	CCCCCC-CTCGAG-TTAGATGAGAGTCTTTTCCAGTTC
**GRE3KOfor**	GTAATATAAATCGTAAAGGAAAATTGGAAATTTTTTAAAGATA GCTTCAAAATGTTTCTA
**GRE3KOrevnew**	TTGTTCATATCGTCGTTGAGTATGGATTTTACTGGCTGGA-TTAAGCAAGGATTTTCTTAA
**MRE115'F**	CCCCCC-CGTACG-TCTTTCCAACAAACCAAGCG
**MRE115'R**	CCCCCC-GTCGAC-AGTCGAGTTTTATCGGATCTGAGC
**MRE113'F**	CCCCCC-ACTAGT-TTGTACTTGATCCCTATATTATATTATATCCTATTTATAACC
**MRE113'R**	CCCCCC-CCGCGG-AGTTCTATTGTGTGTCCAGGC
**pUG6KOPCRnewF**	CCAGCTGAAGCTTCGTACG
**pUG6KOPCRnewR**	CCGGCAGATCCGCGG
**CAC35'F**	CCCCCC-CGTACG-GTGGTTTGTTGTCTGTCTGG
**CAC35'R**	CCCCCC-GTCGAC-CTTTGAAACTAAATTTGTATATTGTTTGTCAGAA
**CAC33'F**	CCCCCC-ACTAGT-CCTAAACGTTCCTTGAAGCCA
**CAC33'R**	CCCCCC-CCGCGG-GTTCGGCTTTGGACATTTCG
**BYHIR35'F**	CCCCCC-CGTACG-ACTAGCAATGATTCCGTTTTACATTT
**BYHIR35'R**	CCCCCC-GTCGAC-AATAAGCTTTATCTAGAATCTGTGTTGAGG
**BYHIR33'F**	CCCCCC-ACTAGT-GATGACCATATTTTGGAAGAAGTGTG

**BYHIR33'R**	CCCCCC-CCGCGG-CAAATCTTTATCGTAATCAGATAATTTTTCCAA
**CAC25'F**	CCCCCC-CGTACG-AGAAAGGTCCTCAGATTGAGC
**CAC25'R**	CCCCCC-GTCGAC-TGTCCTGCCCCTTTGCT
**CAC23'F**	CCCCCC-ACTAGT-TTTTTAATATATTTAATGCGGTACATAAGAATGCC
**CAC23'R**	CCCCCC-CCGCGG-TCACGAGAGATGAGTCCACC
**BefICE2F**	ATGATTCACTGTCACTTAGTGAGC
**AftACE2R**	CTAAGAGTCTGTTTAGATCAACAGTCT
**BefRRM1F**	CATAGAACCGAGTGTAACACCA
**AftRRM1R**	AGGATTCTCCGAATAACCTCTAGC
**APL25'F**	CCCCCC-CGTACG-TATCCTGATGGAGCACTTCG
**APL25'R**	CCCCCC-GTCGAC-AGTTGAAACTGTTTTTAAGTGCAGT
**APL23'F**	CCCCCC-ACTAGT-CTATAAACGTCCGTTGTAGTGAACT
**APL23'R**	CCCCCC-CCGCGG-CCTGACATCTTTGGACGTGG
**IMT4p**	CCG-GAGCTC-AACATCCAGT
**IMT4t**	GGC-GGTACC-TTATAGTCTATAGCTTAAAT
**sup4p**	CCG-GAGCTC-ACCATCTTGG
**IMT4p**	CCG-GAGCTC-AACATCCAGT
**XbaITAL1for**	CCG-TCTAGA-ATGTCCTCCAACTCCCTTGAAC
**XhoITAL1rev**	CCCG-CTCGAG-TTAGAATCTGGCTTCCAATTGTTCC
**SacItefTAL1for**	CCG-GAGCTC-ATAGCTTCAAAATGTTTCTACTC
**SacITAL1cyc1rev**	GGC-GAGCTC-CAAATTAAAGCCTTCGAGCGTCC
**MCS-Fwd-SpeI**	G-ACTAGT-ATGTCTAAAGGTGAAGAATTATTCACTGG
**MCS-Rev-2**	CCCCG-CTCGAG-TTATTTGTACAATTCATCCATACCATGGG
**P2AYFPGlcF**	ACAAGATGGTGATGTTGAAGAAAATCCAGGACCA-TCTAAAGGTGAAGAATTATTCACTGG
**P2AstrawGlcR**	TTTAATAAAGAAAAGTTGGTAGCACCAGAACC-CTTGTACAGCTCGTCCATGC
**SpeImStrawberryF**	CC-ACTAGT-ATGGTGAGCAAGGGCG
**EcoRImStrawberryR**	G-GAATTC-TTTATCGATCGATT-CTACTTGTACAGCTCGTCCAT
**MCS-Fwd-SalI-2**	TAACGC-GTCGAC-ATGTCTAAAGGTGAAGAATTATTCACTGG
**mStraw-YFPf**	GATCCCCCGGG-ATGGTGAGCAAGGGCGAG
**mStraw-YFPr**	ACTCGAGGAATTC-TTATTTGTACAATTCATCCATACCATGGG
**His3AIgenomeflankF**	ATCGATAAGCTTGGGCTGC
**ARTR**	TTAATTCTTAGTATTCCATGTGTCTCGT
**HispromSPT15F**	AAAAAATGAGCAGGCAAGATAAACGAAGGCAAAG-ATGGCCGATGAGGAACGT
**HistermSPT15R**	AAGCTTGGGCTGCAGCTTTAAATAATCGGTG-TCACATTTTTCTAAATTCACTTAGCACA
**Spt15TEFR**	AACTCCTTTAAACGTTCCTCATCGGCCAT-AAACTTAGATTAGATTGCTATGCTTTCTTT

**SPT15intronR**	GTTAGTAAAAATTCAAAAGCCTCCTTTAGTCCATATTAACATACTGGCCGATGAGGAACG
**SPT15intronF**	AAATGGTATTATTTATAACAGTAAACTTAGATTAGATTGCTATGCTTTCTT
**IntronSiteF**	CGCCGAATTTTTACTAACAAATGGTATTATTTATAACAGT
**IntronSiteR**	CGCCAAAAGCCTCCTTTAGTCCATATTAAC
**TEFmutssacF**	CCC-GAGCTC-ATAGCTTCAAAATGTTTCTACTCCTT
**p416ura3EcoR1rev**	CCG-GAATTC-TTAGTTTTGCTGGCCGCATC
**XhoISPT15R**	CCCCG-CTCGAG-TCACATTTTTCTAAATTCACTTAGCACA
**BefCAC2F**	CGTTTCTGAGAGGTAACTGAGG
**CAC2BegR**	GATTCTTGCTGTGTATTTGG
**CAC2EndF**	TGATTTAGCATGGTCTGAGG
**AftCAC2R**	TTGTTGCTGTTGGTCATTGG
**BefCAC3F**	ACAACCACTTCACCCAAACCC
**CAC3BegR**	TTGGGAAGATGTAAATGAGG
**CAC3EndF**	AAGAAGATGGGTTAGTCAAGC
**AftCAC3R**	TGGAAATGTTGTAGAGTGGAGG
**BefAPL2F**	TTCTCAACCATCCAAGTCGG
**APL2BegR**	CCAACTCAACGCATAAATCC
**APL2EndF**	TAACGATGATGTGCTATTGG
**AftAPL2R**	CTTGTTGATCTTTCTTCCCACC
**BefMRE11F**	ATTGATGGCTGATGACGTGG
**MRE11BegR**	TTGGTAGAGTGACTTCTTGG
**MRE11EndF**	CCAACGAGCAAACCCAAACG
**AftMRE11R**	TGTGTTTGAGGGCTCCTTGG
**BefBYHIR3F**	TCTACGCGGTCCATAATCTCC
**HIR3BegR**	ATCTAGCGTAGGAGAAGAATTGC
**HIR3EndF**	ATTTGACAGCGTTTGCTTGG
**AftBYHIR3R**	TTGACGCAAAGGAAATGTGG
**BefGRE3F**	ATGGGCGCATTACTACAAGAAG
**AftGRE3R**	CTGTTTGACGCACTGATGGGT
**BegGRE3R**	GACCTTCGGAGATGGCTTTC
**EndGRE3F**	AACCATCCAGGCAGTACCAC
**URA3RTPCRF**	ATTGTTAGCGGTTTGAAGCAGGCG
**URA3RTPCRR**	GAGCCCTTGCATGACAATTCTGCT
**Alg9F**	ATCGTGAAATTGCAGGCAGCTTGG
**Alg9R**	CATGGCAACGGCAGAAGGCAATAA
**Spt15-qPCR2f**	GCAATCTAATCTAAGTTTATGGCCGATGAGGA
**Spt15-qPCR2r**	GTTGCTGGTTTTGTACCATCTCGATTCTGG
**NGSAmp1F**	ACTGAT-GCCAACTTACTTCTGACAACG
**NGSAmp1R**	GCTACC-CCGCCTCCATCCAGTC

**NGSAmp2F**	CGTACG-GCCAACTTACTTCTGACAACG
**NGSAmp2R**	TGACAT-CCGCCTCCATCCAGTC
**NGSnointron1F**	GATACA-ATCGCGGCCGCCC
**NGSnointron1R**	GGAACT-AGAATGGGCAGACATTACGAATG
**NGSnointron2F**	AGTCAA-ATCGCGGCCGCCC
**NGSnointron2R**	TAACCG-AGAATGGGCAGACATTACGAATG
**NGSnointron3F**	AGCTTT-ATCGCGGCCGCCC
**NGSnointron3R**	TACAAG-AGAATGGGCAGACATTACGAATG
**NGSnointron4F**	GGCTAC-ATCGCGGCCGCCC
**NGSnointron4R**	AAGCTA-AGAATGGGCAGACATTACGAATG
**NGSnointron5F**	ATACGA-ATCGCGGCCGCCC
**NGSnointron5R**	CTGATC-AGAATGGGCAGACATTACGAATG
**NGSnointron6F**	TTACTG-ATCGCGGCCGCCC
**NGSnointron6R**	AGTTCC-AGAATGGGCAGACATTACGAATG
**NGSnointron7F**	ACTTGA-ATCGCGGCCGCCC
**NGSnointron7R**	GATCTG-AGAATGGGCAGACATTACGAATG
**NGSnointron8F**	ACATCT-ATCGCGGCCGCCC
**NGSnointron8R**	AATCGT-AGAATGGGCAGACATTACGAATG
**NGSnointron9F**	GCCAAT-ATCGCGGCCGCCC
**NGSnointron9R**	CACTGT-AGAATGGGCAGACATTACGAATG
**NGSnointron10F**	AGAATC-ATCGCGGCCGCCC
**NGSnointron10R**	GCCTAA-AGAATGGGCAGACATTACGAATG
**NGSnointron11F**	CTGCAG-ATCGCGGCCGCCC
**NGSnointron11R**	ACATCG-AGAATGGGCAGACATTACGAATG
**NGSnointron12F**	ATCACG-ATCGCGGCCGCCC
**NGSnointron12R**	CACGTA-AGAATGGGCAGACATTACGAATG
**NGSnointron13F**	TCACAT-ATCGCGGCCGCCC
**NGSnointron13R**	TATAGA-AGAATGGGCAGACATTACGAATG
**NGSnointron14F**	TGCAAA-ATCGCGGCCGCCC
**NGSnointron14R**	GTGCCA-AGAATGGGCAGACATTACGAATG
**NGSnointron15F**	TGTTAG-ATCGCGGCCGCCC
**NGSnointron15R**	ATAGAA-AGAATGGGCAGACATTACGAATG
**NGSnointron16F**	TCGAAG-ATCGCGGCCGCCC
**NGSnointron16R**	GAATGA-AGAATGGGCAGACATTACGAATG
**NGSnointron17F**	TACAGC-ATCGCGGCCGCCC
**NGSnointron17R**	TCTGAG-AGAATGGGCAGACATTACGAATG
**NGSnointron18F**	CTATAC-ATCGCGGCCGCCC
**NGSnointron18R**	AGCTAG-AGAATGGGCAGACATTACGAATG
**NGSnointron19F**	CGGAAT-ATCGCGGCCGCCC
**NGSnointron19R**	GCCATG-AGAATGGGCAGACATTACGAATG

**NGSnointron20F**	CACGAT-ATCGCGGCCGCCC
**NGSnointron20R**	GCTCAT-AGAATGGGCAGACATTACGAATG
**SacI-HSXf**	CC-GAGCTC-GGGTTCATTACAGCAGC
**PstI-HSXr**	CC-CTGCAG-TATATTTACCATCAACTCCGC
**EcoRITkc1F**	G-GAATTC-TTTGAAAGATGATACTCTTTATTTCTAGACAG
**SbfITkc1R**	GG-CCTGCAGG-TATATATATATAACTGTCTAGAAATAAAGAGTATCATCTT
**EcoRITkc5F**	G-GAATTC-TTTGAAAGATGATACTCTTTATTTATATATATATATATATATATATATA
**SbfITkc5R**	GG-CCTGCAGG-TATATATATATATATATATATATAAATAAAGAGTATCATCTTTCAAA
**EcoRITkc8SbfIF**	G-GAATTC-TTTGAAAAAATTTATTAAAAAAAAAATATATA-CCTGCAGG-CC
**SbfITkc8EcoRIR**	GG-CCTGCAGG-TATATATTTTTTTTTAATAAATTTTTTCAAA-GAATTC-C
**URA3AICYC1F**	CCG-GAATTC-CGAGCGTCCCAAAACCTT
**URA3AICYC1Rnew**	CCG-CCTGCAGG-TCATGTAATTAGTTATGTCACGCTTACA
**URA3AIIDP1F**	CCG-GAATTC-GATGGTAATGATCCGAACTTGG
**URA3AIIDP1Rnew**	CCG-CCTGCAGG-TCGAATTTACGTAGCCCAATCTAC
**URA3AIPRM9F**	CCG-GAATTC-ATTTTCAACATCGTATTTTCCGAAGC
**URA3AIPRM9Rnew**	CCG-CCTGCAGG-ACAGAAGACGGGAGACACT
**GREcontrol-fwd**	GTCTGTTATTAATTTCACAGGTAGTTCTGGTCCATTGGTGAAAGTTTGCGGCTTGCAGAGCACAGAGGCCGCAGAATGT-GTGATGACAAAACCTCTTCCG
**TART-BB-f**	T-CAAACAGGAATCGAATGCAACCG
**TART-BB-r**	AATTGTCCTTTTAACAGCGATCGC
**ip-G418f**	T-GAAAGAAATGCATAAGCTTTTGC
**ip-G418r**	AGACTTGTTCAACAGGCC
**G418-stop-f**	CGAATTCACTGGCCGTCG
**G418-stop-r**	CAAGCTTGGCGTAATCATGG
**G418-stop2-f**	GACGTTGTAAAACGACGGCCAGTGAATTCGTCTGTAGCCCTCAACGGAAA
**G418-stop2-r**	AGGTCCGCCGGCGTTGGACGAGCGGGTGCAACTAATTGACGGGAGT
**JMW-0077**	CGAATTCACTGGCCGTCG
**JMW-0078**	CAAGCTTGGCGTAATCATGG
**JMW-0092**	GACGTTGTAAAACGACGGCCAGTGAATTCGTCTGTAGCCCTCAACGGAAA
**JMW-0093**	AGGTCCGCCGGCGTTGGACGAGCGGGTGCAACTAATTGACGGGAGT
**JMW-0094**	TGCACCCGCTCGTCCAACGCCGGCGGACCTCGGTGCAACACTACTTCAACTTC
**JMW-0095**	ACAGCTATGACCATGATTACGCCAAGCTTGTTGTTCTCCTCATTCCAGGTG
**JMW-0101**	TCTGTAGCCCTCAACGGAAA

**JMW-0102**	TTGTTCTCCTCATTCCAGGTG
**JMW-0163**	TGACACCGATTATTTAAAGCTGCAG
**JMW-0164**	CTTTGCCTTCGTTTATCTTGCC
**JMW-0165**	TATATATATCGTATGCTGCAGCTTTAAATAATCGGTGTCAGCCT GATCTGTTTAGCTTGC
**JMW-0166**	TATACTAAAAAATGAGCAGGCAAGATAAACGAAGGCAAAGAC TGGATGGCGGCGTTAGTA
**JA-211**	AAACTTAGATTAGATTGCTATGC
**JA-240**	TAAAGCTGCAGCCCAA
**JMW-0210**	ATTCATTAGATCGATAAGCTTGG
**JMW-0211**	TTGCTCATTAGAAAGAAAGCAT
**JMW-0225**	TAGCCAGGAGGAGAGTGACGGCTCCTATGTTGTGTGG
**JMW-0226**	ATGACGGAAGATGCTCGGGCGGGGTATCGTATGCTTC
**JMW-0227**	GCGGAACCCCTATTTGTTTATT
**JMW-0228**	GCTGTTTCCTGTGTGAAATTGTT
**JMW-0229**	TTGTGAGCGGATAACAATTTCACACAGGAAACAGCTTTGCTTC TCTCTTCTTCCTGTG
**JMW-0230**	CACACAACATAGGAGCCGTCACTCTCCTCCTGGCTATTG
**JMW-0231**	AAGCATACGATACCCCGCCCGAGCATCTTCCGTCATTA
**JMW-0232**	GTATTTAGAAAAATAAACAAATAGGGGTTCCGCGCCTTTTAGG CCCAGAATTAGG
**JMW-0280**	TTTGCTTCTCTCTTCTTCCTGTG
**JMW-0281**	TCCACAACCAGTTTCATTCG

Supplementary Table 8: DNA fragments generated in this study.

Fragment Name	Template	Forward Primer/Restriction Enzyme	Reverse Primer/Restriction Enzyme
CAS9	hCas9 vector	GalCas9F	CYC1tCas9R
CYC1t	p415-*pTDH3*	Cas9CYC1tF	gRNACYC1tR
***URA3*gRNA**	*URA3*gRNA	gRNAF	gRNAR
**p416-*pGAL1*frag**	p416-*pGAL1*	XbaI	XbaI
pGAL1	*S. cerevisiae* BY4741 genome	SacIGalF	SpeXbaIGalR
**MCSBB**	p416-*pGAL1-CAS9-CYC1t*-URA3gRNA	PacIP416F	NotICYC1tR
***TRP1*gRNA**	TRP1gRNA	NotIgRNAF	PacIgRNAR
***pGAL1*frag1**	*S. cerevisiae* BY4741 genome	GalpF1	Gal1pFixR2
***pGAL1*frag2**	GALfrag1	GalpF2	GalpR2
**Ty1frag1**	*S. cerevisiae* BY4741 genome	TyH3GenomeF	TyH3GenomeR
**Ty1frag2**	Ty1frag1	TyH3PCRF1	TyH3PCRR1
**Ty1frag3**	Ty1frag2	TyH3PCRF2	TyH3PCRR2
**Ty1frag4**	Ty1frag3	TyH3PCRF3	TyH3FlankR
***HIVRT*frag1**	Synthetic *HIVRT*	HIVnoATGF	HIVRThomR
***URA3AI*frag1**	*S. cerevisiae* s288c genome	URA3F	URA3AIR
***URA3*frag1**	*S. cerevisiae* s288c genome	URA3R	URA3AIF
***URA3*frag2**	URA3frag1	URA3R	AInoass2F
***URA3*frag3**	URA3frag2	URA3R	AInoass3F
**LTRfrag1**	Synthetic Ty1	LTRF1	LTRR1
**LTRfrag2**	LTRfrag1	PPTNotIF	LTRflankR
***HIS3*promfrag1**	*S. cerevisiae* BY4741 genome	HISpromF	HISpromR
**p423frag1**	p423-*pTDH3*	P416F	P416R
***Ty1RT*frag1**	Synthetic *Ty1RT*	Ty1RTHDF1	Ty1RTHDR1
***Ty1RT*frag2**	*Ty1RT*frag1	Ty1RTHDF2	Ty1RTHDR2
**pGALmTy1frag1**	pGALmTy1-*HIVRT-URA3I-pHIS3*	RTmutF	RTmutR
**pGALmTy1-*Ty1RT* BB**	pGALmTy1-*Ty1RT-URA3I-pHIS3*	HispromF	HistermR
***pTEF1*hom**	p416-*pTEF1*	LTRTEFF2	URA3TEFR2
***Ty1RT-URA3*-BB**	pGALmTy1-*Ty1RT-URA3I-pHIS3*	RT-URA3-BBf	RT-URA3-BBr
**Cargo-*eGFP***	p416-*pTEF1-eGFP*	RT-eGFPf	RT-eGFPr
**Cargo-*LacZ***	p416-*pTEF1-LacZ*	RT-LacZf	RT-LacZr
**Cargo-*CAN1***	*S. cerevisiae* BY4741 genome	RT-CAN1f	RT-CAN1r

**Cargo-*LacZ*-BB**	pGALmTy1-*Ty1RT-LacZ-URA3I-pHIS3*	Lacz-URA3-BBf	Lacz-URA3-BBr
**Cargo-*eGFP-LacZ*-ins**	p416-*pTEF1-eGFP*	LacZ-eGFPf	RT-eGFPr
**pGALmTy1-*HIVRT* BB**	pGALmTy1-*HIVRT-URA3I-pHIS3*	HispromF	HistermR
***pCYC1*hom**	p416-*pCYC1*	LTRCYCF2	URA3CYCR2
pTDH3	p416-*pTDH3*	LTRGPDF2	URA3GPDR2
**MutBB**	pGALmTy1-*Ty1RT-URA3I-pHIS3*	PPTNotIF	HistermR
**Smut6up**	p413-*spt15-B6*	reGREsptR	reGREsptF
**Smut6down**	p413-*spt15-B6*	reGREtefR	reGREtefF
**U3-5up**	p413-*ura3(3-5)*	Intron*ura3(3-5)*F	LTRTEFF2
**U3-5down**	p413-*ura3(3-5)*	Ty1URA3F	Intron*ura3(3-5)*R
**(m)GAXnewintron**	(m)GAXnewintron(gblock)	(m)GAXnewintronF	(m)GAXnewintronR
**(m)GA3Xnewintron**	(m)GAX3newintron(gblock)	(m)GA3XnewintronXylA3for	(m)GAXnewintronR
**P2A-RT**	pGALmTy1-*Ty1RT-GAX-oldintron*	(m)GA3XnewintronRTfor	(m)GA3XnewintronP2Arev
**Ty1*TAX***	pGALmTy1-*Ty1RT-TAX*	XylAMCSintronF	XylAMCSintronR
**Ty1*XylAXKS***	pGALmTy1-*Ty1RT-pTEF1-XylA-XKS1*	P2AXKS1F	P2AXylAR
**FullMCSFrag**	pGALmTy1-*Ty1RT-URA3I-pHIS3*	TART-FullMCSF	TART-FullMCSR
pTEF1	p413-*pTEF1*	NotITEFF	XmaITEFR
XylA	p416-*pTDH3-XylA*	XmaIXylAF	SacIIXylAR
XKS1	*S. cerevisiae* BY4741 genome	PacIXKS1F	EcoRIXKSR
pTDH3*	pUAStef-UAScit-UASclb	NotImegaGPDF	XmaIGPDR
**pGALmTy1-*Ty1RT-mGA3X-oldintron*frag**	pGALmTy1-*Ty1RT-GA3X-oldintron*	XmaI	EcoRI
**Ty1*TA3X***	pGALmTy1-*Ty1RT-TA3X*	XylAMCSintronF	XylAMCSintronR
**Ty1*XylA3XKS***	pGALmTy1-*Ty1RT-pTEF1-XylA3-XKS1*	P2AXKS1F	P2AXylAR
XylA3	p416-*pTDH3-XylA3**	XmaIXylA3F	SacIIXylAR
**pGALmTy1-*Ty1RT-mGAX-oldintron*frag**	pGALmTy1-*Ty1RT-mGAX-oldintron*	XmaI	XbaI
**pGALmTy1-*Ty1RT-mGA3X-oldintron*frag2**	pGALmTy1-*Ty1RT-GA3X-oldintron*	AscI	None
XylAnointron	N/A	XylAGPDnointronF	XylAGPDnointronR
**pGALmTy1-*Ty1RT-mGAX-oldintron*frag2**	pGALmTy1-*Ty1RT-mGAX-oldintron*	AscI	None
**pGALmTy1-*Ty1RT-SPT15I-pTEF1* cassette**	pGALmTy1-*Ty1RT-SPT15I-pTEF1*	GREnewF	GREnewR

**pGALmTy1-*Ty1RT-spt15-B6I-pTEF1* cassette**	pGALmTy1-*Ty1RT-spt15-B6I*	GREnewF	GREnewR
**pGALmTy1-*Ty1RT-URA3I-pTEF1* cassette**	pGALmTy1-*Ty1RT-URA3I-pTEF1*	GREnewF	GREnewR
**pGAlmTy1-*Ty1RT-ura3(3-5)I-pTEF1* cassette**	pGALmTy1-*Ty1RT-ura3(3-5)I-pTEF1*	GREnewF	GREnewR
**pGALmTy1-*Ty1RT-XylA3*I*-P2A-*XKS1-pTDH3** cassette**	pGALmTy1-*Ty1RT-XylA3*I*-P2A-*XKS1-pTDH3**	GREnewF	GREnewR
**pGALmTy1-*Ty1RT-XylAI*-P2A-*XKS1-pTDH3** cassette**	pGALmTy1-*Ty1RT-XylAI*-P2A-*XKS1-pTDH3**	GREnewF	GREnewR
***GRE3*KO+*XKS1* cassette**	p415-*pTEF1-XKS1*	GRE3KOfor	GRE3KOrevnew
**XbaI*XKS1*XhoI**	*S. cerevisiae* BY4741 genome	XbaIXKS1	XKS1XhoI
**pGALmTy1-*URA3I-pTEF1* cassette**	pGALmTy1-*URA3I-pTEF1*	GREnewF	GREnewR
**pGALmTy1-*Ty1RT-XylA3**-P2A-*XKS1-pTDH3** cassette**	pGALmTy1-*Ty1RT-XylA3**-P2A-*XKS1-pTDH3**	GREnewF	GREnewR
**pGALmTy1-*Ty1RT-XylA*-P2A-*XKS1-pTDH3** cassette**	pGALmTy1-*Ty1RT-XylA*-P2A-*XKS1-TDH3p**	GREnewF	GREnewR
***MRE11* cassette**	pUG6-*MRE115-KanMX-MRE113*	pUG6KOPCRnewF	pUG6KOPCRnewR
***MRE11*5**	*S. cerevisiae* BY4741 genome	MRE115'F	MRE115'R
***MRE11*3**	*S. cerevisiae* BY4741 genome	MRE113'F	MRE113'R
***CAC3* cassette**	pUG6-*CAC35-KanMX-CAC33*	pUG6KOPCRnewF	pUG6KOPCRnewR
***CAC3*5**	*S. cerevisiae* BY4741 genome	CAC35'F	CAC35'R
***CAC3*3**	*S. cerevisiae* BY4741 genome	CAC33'F	CAC33'R
***HIR3* cassette**	pUG6-*HIR35-KanMX-HIR33*	pUG6KOPCRnewF	pUG6KOPCRnewR
***HIR3*5**	*S. cerevisiae* BY4741 genome	BYHIR35'F	BYHIR35'R
***HIR3*3**	*S. cerevisiae* BY4741 genome	BYHIR33'F	BYHIR33'R
***CAC2* cassette**	pUG6-*CAC25-KanMX-CAC23*	pUG6KOPCRnewF	pUG6KOPCRnewR
***CAC2*5**	*S. cerevisiae* BY4741 genome	CAC25'F	CAC25'R

CAC23	*S. cerevisiae* BY4741 genome	CAC23'F	CAC23'R
***APL2* cassette**	pUG6-*APL25-KanMX-APL23*	pUG6KOPCRnewF	pUG6KOPCRnewR
APL25	*S. cerevisiae* BY4741 genome	APL25'F	APL25'R
APL23	*S. cerevisiae* BY4741 genome	APL23'F	APL23'R
pIMT4*-tRNA^{iMet}-*IMT4t	*pIMT4*-tRNA^{iMet}-*IMT4t* (gblock)	IMT4p	IMT4t
pSUP4*-tRNA^{iMet}-*RPR1t	*pSUP4*-tRNA^{iMet}-*RPR1t* (gblock)	sup4p	rpr1t
pSUP4*-tRNA^{iMet}-*IMT4t	*pSUP4*-tRNA^{iMet}-*IMT4t* (gblock)	sup4p	IMT4t
TAL1	*S. cerevisiae* BY4741 genome	XbaITAL1for	XhoITAL1rev
pTEF1-TAL1	p416-*pTEF1-TAL1*	SacItefTAL1for	SacITAL1cyc1rev
YFP	P416-*pTDH3-YFP*	MCS-Fwd-SpeI	MCS-Rev-2
mStraw-YFP	pGALmTy1-*Ty1RT-mStrawberry*-P2A-*YFP*	mStraw-YFPf	mStraw-YFPr
**P2Afrag**	p416-*pTDH3-mStrawberry-YFP*	P2AYFPGlcF	P2AstrawGlcR
**df*YFP***	P416-*pTDH3-YFP*	MCS-Fwd-SalI-2	MCS-Rev-2
mStawberry	p*mStrawberry*	SpeImStrawberryF	EcoRImStrawberryR
**ART BB**	pGALmTy1-*Ty1RT-URA3I-pTEF1*	ARTR	His3AIgenomeflankF
SPT15intron	pGALmTy1-*Ty1RT*-MCS-*SPT15intronnosite-pTEF1*	IntronSiteF	IntronSiteR
Spt15intronnosite	pGALmTy1-*Ty1RT-SPT15-pTEF1*	SPT15intronF	SPT15intronR
**pGALmTy1-*Ty1RT-SPT15*frag**	pGALmTy1-*Ty1RT-SPT15*	NotI	N/A
**pGALmTy1-*HIVRT-URA3I-pHIS3*frag**	pGALmTy1-*HIVRT-URA3I-pHIS3*	XmaI	N/A
***SPT15-pTEF1* cassette**	p416-*pTEF1-SPT15*	LTRTEFF2	Spt15tefR
SPT15	*S. cerevisiae* BY4741 genome	HispromSPT15F	HistermSPT15R
***SPT15*clone**	*SPT15* round 1 isolate gDNA	TEFmutssacF	XhoISPT15R
spt15-B6	*SPT15* round 1 isolate gDNA	TEFmutssacF	XhoISPT15R
spt15-B6-1	*SPT15* round 2 isolate gDNA	TEFmutssacF	XhoISPT15R
URA3	*URA3* round 1 isolate gDNA	TEFmutssacF	p416ura3EcoR1rev
ura3(3-5)	*URA3* round 1 isolate gDNA	TEFmutssacF	p416ura3EcoR1rev
ura3(3-5-2)	*URA3* round 2 isolate gDNA	TEFmutssacF	p416ura3EcoR1rev
Tkc1	n/a (anneal)	EcoRITkc1F	SbfITkc1R
Tkc5	n/a (anneal)	EcoRITkc5F	SbfITkc5R

Tkc8	n/a (anneal)	EcoRITkc8SbfIF	SbfITkc8EcoRIR
CYC1t	*S. cerevisiae* BY4741 genome	URA3AICYC1F	URA3AICYC1Rnew
IDP1t	*S. cerevisiae* BY4741 genome	URA3AIIDP1F	URA3AIIDP1Rnew
PRM9t	*S. cerevisiae* BY4741 genome	URA3AIPRM9F	URA3AIPRM9Rnew
**d*KanMX-pTEF1* cassette**	pGALmTy1-*Ty1RT*-d*KanMX-pTEF1*	GREcontrol-fwd	GREnewR
**pGALmTy1-*Ty1RT*-d*KanMX-pTEF1* cassette**	pGALmTy1-*Ty1RT*-d*KanMX-pTEF1*	GREnewF	GREnewR
**pGALmTy1-*Ty1RT-pTEF1* BB**	pGALmTy1-*Ty1RT-URA3I-pTEF1*	TART-BB-f	TART-BB-r
KanMX	pUG6	ip-G418f	ip-G418r
KanMX-1	pGALmTy1-*Ty1RT-KanMX-pTEF1*	G418-stop-f	G418-stop-r
**d*KanMX***	pGALmTy1-*Ty1RT-KanMX-1-pTEF1*	G418-stop2-f	G418-stop2-r
**Linear_pUC19**	pUC19	JMW-0077	JMW-0078
***KlURA3*_US-Linker**	*K. lactis* genomic DNA	JMW-0092	JMW-0093
**Linker-*KlURA3*_DS**	*K. lactis* genomic DNA	JMW-0094	JMW-0095
***KlURA3*_US-Linker-*KlURA3*_DS**	pUC19-Δ*KlURA3*	JMW-0101	JMW-0102
**p42X-TART-*pTEF1-ScURA3AI*-NoMarker**	p423-TART-*pTEF1-ScURA3AI*	JMW-0163	JMW-0164
***KanMX_Kl*Marker**	p42K	JMW-0165	JMW-0166
***KlURA3AI* Cargo**	Synthetic *KlURA3AI*	JMW-0210	JMW-0211
**p42K-TART-*pTEF1*-NoCargo**	p42K-TART-*pTEF1-ScURA3AI*	JA-211	JA-240
**TART-*pTEF1-KlURA3AI-KanMX***	p42K-TART-*pTEF1-KlURA3AI*	JMW-0225	JMW-0226
***AmpR*-ColE1**	p42K-TART-*pTEF1-KlURA3AI*	JMW-0227	JMW-0228
***KlNDT80*_US**	*K. lactis* genomic DNA	JMW-0229	JMW-0230
***KlNDT80*_DS**	*K. lactis* genomic DNA	JMW-0231	JMW-0232
***NDT80*_US-TART-*pTEF1-KlURA3AI-KanMX-NDT80*_DS**	p42K-*NDT80*_US-TART-*pTEF1-KlURA3AI-NDT80*_DS	JMW-0280	JMW-0281

Supplementary Table 9: Plasmids generated through restriction enzyme cloning.

Plasmid Name	Backbone	Insert	Rest. Enz. 1	Rest. Enz. 2
**p416-*pGAL1***	p416-*pTDH3*	*pGAL1*	SacI	XbaI
**p416-*pGAL1-CAS9-CYC1t*-MCS**	MCSBB	N/A	N/A	N/A
**p416-*pGAL1-CAS9-CYC1t-TRP1*gRNA**	p416-*pGAL1-CAS9-CYC1t*-MCS	*TRP1*gRNA	NotI	PacI
**pGALmTy1-*URA3I-pTEF1***	ART BB	N/A	N/A	N/A
**pGALmTy1-*Ty1RT-SPT15I-pTEF1***	*SPT15intron*	N/A	N/A	N/A
**pGALmTy1-*Ty1RT*-MCS-*SPT15intronnosite-pTEF1***	*SPT15intronnosite*	N/A	N/A	N/A
**p413-*pTEF1-SPT15***	p413-*pTEF1*	*SPT15*clone	SacI	XhoI
**p413-*spt15-B6***	p413-*pTEF1*	*spt15-B6*	SacI	XhoI
**p413-*spt15-B6-1***	p413-*pTEF1*	*spt15-B6-1*	SacI	XhoI
**p413-*pTEF1-URA3***	p413-*pTEF1*	*URA3*	SacI	EcoRI
**p413-*ura3(3-5)***	p413-*pTEF1*	*ura3(3-5)*	SacI	EcoRI
**p413-*ura3(3-5-2)***	p413-*pTEF1*	*ura3(3-5-2)*	SacI	EcoRI
**pGALmTy1-*Ty1RT-mGAX-oldintron***	pGALmTy1-*Ty1RT-TAXintron*	*pTDH3**	NotI	XmaI
**pGALmTy1-*Ty1RT-TAXintron***	Ty1*TAX*	N/A	N/A	N/A
**pGALmTy1-*Ty1RT-TAX***	Ty1*XylAXKS*	N/A	N/A	N/A
**pGALmTy1-*Ty1RT-pTEF1-XylA-XKS1***	pGALmTy1-*Ty1RT-pTEF1-XylA*	*XKS1*	PacI	EcoRI
**pGALmTy1-*Ty1RT-pTEF1-XylA***	pGALmTy1-*Ty1RT-pTEF1*	*XylA*	XmaI	SacII
**pGALmTy1-*Ty1RT-pTEF1***	pGALmTy1-*Ty1RT*-FullMCS	*pTEF1*	NotI	XmaI
**pGALmTy1-*Ty1RT*-FullMCS**	FullMCSfrag	N/A	N/A	N/A
**pGALmTy1-*Ty1RT-mGA3X-oldintron***	pGALmTy1-*Ty1RT-TA3Xintron*	*pTDH3**	NotI	XmaI
**pGALmTy1-*Ty1RT-TA3Xintron***	Ty1*TA3X*	N/A	N/A	N/A
**pGALmTy1-*Ty1RT-TA3X***	Ty1*XylA3XKS*	N/A	N/A	N/A
**pGALmTy1-*Ty1RT-pTEF1-XylA3-XKS1***	pGALmTy1-*Ty1RT-pTEF1-XylA3*	*XKS1*	PacI	EcoRI
**pGALmTy1-*Ty1RT-pTEF1-XylA3***	pGALmTy1-*Ty1RT-pTEF1*	*XylA3*	XmaI	SacII
**p415-*pTEF1-XKS1***	p415-*pTEF1*	XbaI*XKS1*XhoI	XbaI	XhoI
**pUG6-*MRE115-KanMX-MRE11*3**	pUG6-*MRE115-KanMX*	*MRE11*3	SpeI	SacII
**pUG6-*MRE115-KanMX***	pUG6	*MRE115*	BsiWI	SalI
**pUG6-*CAC35-KanMX-CAC3*3**	pUG6-*CAC35*	*CAC3*3	SpeI	SacII
**pUG6-*CAC35***	pUG6	*CAC35*	BsiWI	SalI
**pUG6-*HIR35-KanMX-HIR3*3**	pUG6-*HIR35-KanMX*	*HIR3*3	SpeI	SacII
**pUG6-*HIR35-KanMX***	pUG6	*HIR35*	BsiWI	SalI
**pUG6-*CAC25-KanMX-CAC23***	pUG6-*CAC25-KanMX*	*CAC2*3	SpeI	SacII
**pUG6-*CAC25-KanMX***	pUG6	*CAC2*5	BsiWI	SalI

**pUG6-*APL25-KanMX-APL23***	pUG6-*APL25-KanMX*	*APL23*	SpeI	SacII
**pUG6-*APL25-KanMX***	pUG6	*APL25*	BsiWI	SalI
**p414-*pIMT4*-tRNA^{iMet}-*IMT4t***	p414-*pCYC1*	*pIMT4*-tRNA^{iMet}-*IMT4t*	KpnI	SacI
**p414-*pSUP4*-tRNA^{iMet}-*RPR1t***	p414-*pCYC1*	*pSUP4*-tRNA^{iMet}-*RPR1t*	KpnI	SacI
**p414-*pSUP4*-tRNA^{iMet}-*IMT4t***	p414-*pCYC1*	*pSUP4*-tRNA^{iMet}-*IMT4t*	KpnI	SacI
**p424-*pIMT4*-tRNA^{iMet}-*IMT4t***	p424-*pTEF1*	*pIMT4*-tRNA^{iMet}-*IMT4t*	KpnI	SacI
**p424-*pSUP4*-tRNA^{iMet}-*RPR1t***	p424-*pTEF1*	*pSUP4*-tRNA^{iMet}-*RPR1t*	KpnI	SacI
**p424-*pSUP4*-tRNA^{iMet}-*IMT4t***	p424-*pTEF1*	*pSUP4*-tRNA^{iMet}-*IMT4t*	KpnI	SacI
**p416-*pTEF1-TAL1***	p416-*pTEF1*	*TAL1*	XbaI	XhoI
**p424-*pTEF1-TAL1-CYC1t-pIMT4*-tRNA^{iMet}-*IMT4t***	p424-*pIMT4*-tRNAiMet-*IMT4t*	*pTEF1-TAL1*	SacI	N/A
**p413-*pGAL1-YFP***	p413-*pTEF1-YFP*	*pGAL1*	SacI	SpeI
**p413-*pTEF1-YFP***	p413-*pTEF1*	*YFP*	SpeI	XhoI
**pGALmTy1-*Ty1RT-LacZ-YFP-mStrawberry-URA3I-pHIS3***	pGALmTy1-*Ty1RT-LacZ-URA3I-pHIS3*	*mStraw-YFP*	EcoRI	N/A
**pGALmTy1-*Ty1RT-mStrawberry*-P2A-*YFP***	P2Afrag	N/A	N/A	N/A
**p416-*pTDH3-mStrawberry-YFP***	p416-*pTDH3-mStrawberry*	df*YFP*	SalI	xhoI
**p416-*pTDH3-mStrawberry***	p416-*pTDH3*	*mstrawberry*	SpeI	EcoRI
**pGALmTy1-*Ty1RT-URA3I-pTEF1-Tsynth1***	pGALmTy1-*Ty1RT-URA3I-pTEF1*	*Tkc1*	EcoRI	SbfI
**pGALmTy1-*Ty1RT-URA3I-pTEF1-Tsynth5***	pGALmTy1-*Ty1RT-URA3I-pTEF1*	*Tkc5*	EcoRI	SbfI
**pGALmTy1-*Ty1RT-URA3I-pTEF1-Tsynth8***	pGALmTy1-*Ty1RT-URA3I-pTEF1*	*Tkc8*	EcoRI	SbfI
**pGALmTy1-*Ty1RT-URA3I-pTEF1-CYC1t***	pGALmTy1-*Ty1RT-URA3I-pTEF1*	*CYC1t*	EcoRI	SbfI
**pGALmTy1-*Ty1RT-URA3I-pTEF1-IDP1t***	pGALmTy1-*Ty1RT-URA3I-pTEF1*	*IDP1t*	EcoRI	SbfI
**pGALmTy1-*Ty1RT-URA3I-pTEF1-PRM9t***	pGALmTy1-*Ty1RT-URA3I-pTEF1*	*PRM9t*	EcoRI	SbfI
**pGALmTy1-*Ty1RT-KanMX-1-pTEF1***	*KanMX-1*	n/a	n/a – blunt ligation	
**pGALmTy1-*Ty1RT*-d*KanMX-pTEF1***	d*KanMX*	n/a	n/a – blunt ligation	

Supplementary Table 10: Plasmids generated through homologous recombination cloning or Gibson Assembly.

Plasmid Name	Fragments Used For Assembly
**p416-*pGAL1-CAS9-CYC1t-URA3*gRNA**	*CAS9*, *CYC1t*, *URA3*gRNA, p416-*pGAL1*frag
**pGALmTy1-*HIVRT-URA3I-pHIS3***	*pGAL1*frag2, Ty1frag4, *HIVRT*frag1, *URA3AI*frag1, *URA3*frag3, LTRfrag2, *HIS3*promfrag1, and p423frag1
**pGALmTy1-*Ty1RT-URA3I-pHIS*3**	*Ty1RT*frag2, pGALmTy1frag1
**pGALmTy1-*Ty1RT-URA3I-pTEF1***	pGALmTy1-*Ty1RT* BB, *pTEF1*hom
**pGALmTy1-*Ty1RT-eGFP-URA3I-pHIS3***	*Ty1RT-URA3*-BB, Cargo-*eGFP*
**pGALmTy1-*Ty1RT-CAN1-URA3I-pHIS3***	*Ty1RT-URA3*-BB, Cargo-*CAN1*
**pGALmTy1-*Ty1RT-LacZ-URA3I-pHIS3***	*Ty1RT-URA3*-BB, Cargo-*LacZ*
**pGALmTy1-*Ty1RT-LacZ-eGFP-URA3I-pHIS3***	Cargo-*LacZ*-BB, Cargo-*eGFP-LacZ*-ins
**pGALmTy1-*Ty1RT-URA3I-pCYC1***	pGALmTy1-*Ty1RT* BB, *pCYC1*hom
**pGALmTy1-*Ty1RT-URA3I-pTDH3***	pGALmTy1-*Ty1RT* BB, *pTDH3*
**pGALmTy1-*Ty1RT-spt15-B6I***	MutBB, Smut6up, Smut6down
**pGAlmTy1-*Ty1RT-ura3(3-5)I-pTEF1***	MutBB, U3-5up, U3-5down
**pGALmTy1-*Ty1RT-XylA3*I*-P2A-*XKS1-pTDH3****	*(m)GA3X*newintron, P2A-*Ty1RT*, pGALmTy1-*Ty1RT-mGA3X-oldintronfrag*
**pGALmTy1-*Ty1RT-XylAI*-P2A-*XKS1-pTDH3****	*(m)GAX*newintron, pGALmTy1-*Ty1RT-mGAX-oldintronfrag*
**pGALmTy1-*Ty1RT-XylA3**-P2A-*XKS1-pTDH3****	pGALmTy1-*Ty1RT-mGA3X-oldintronfrag2*, *XylAnointron*
**pGALmTy1-*Ty1RT-XylA*-P2A-*XKS1-pTDH3****	pGALmTy1-*Ty1RT-mGAX-oldintronfrag2*, *XylAnointron*
**pGALmTy1-*Ty1RT-SPT15-pTEF1***	pGALmTy1-*Ty1RT-SPT15*frag, *SPT15-pTEF1* cassette
**pGALmTy1-*Ty1RT-SPT15***	pGALmTy1-*HIVRT-URA3I-pHIS3*frag, *SPT15*
**pGALmTy1-*Ty1RT-KanMX-pTEF1***	pGALmTy1-*Ty1RT-pTEF1* BB, *KanMX*
**pUC19-Δ*KlURA3***	Linear_pUC19, *KlURA3*_US-Linker, Linker-*KlURA3*_DS
**p42K-TART-*pTEF1-ScURA3AI***	p42X-TART-*pTEF1-URA3AI*-NoMarker, *KanMX_Kl*Marker
**p42K-TART-*pTEF1-KlURA3AI***	p42K-TART-*pTEF1*-NoCargo, *KlURA3AI* Cargo
**pUC-*NDT80*_US-TART-*pTEF1-KlURA3AI-KanMX-NDT80*_DS**	*AmpR*-ColE1 (pUC19-based), *KlNDT80*_US, TART-*pTEF1-KlURA3AI-KanMX*, *KlNDT80*_DS

Supplementary Table 11: Strains generated through genome editing.

Strain Name	Parent Strain	PCR Fragment
S. cerevisiae **BY4741 Δ*rrm3 trp1::Ty1-Ty1RT-SPT15I-pTEF1***	Strain **6** (See below)	pGALmTy1-*Ty1RT-SPT15I-pTEF1* cassette
S. cerevisiae **BY4741 Δ*rrm3 trp1::Ty1-Ty1RT-spt15-B6I-pTEF1***	Strain **6** (See below)	pGALmTy1-*Ty1RT-spt15-B6I-pTEF1* cassette
S. cerevisiae **BY4741 Δ*rrm3 trp1::Ty1-Ty1RT-URA3I-pTEF1***	Strain **6** (See below)	pGALmTy1-*Ty1RT-URA3I-pTEF1* cassette
S. cerevisiae **BY4741 Δ*rrm3 trp1::Ty1-Ty1RT-ura3(3-5)I-pTEF1***	Strain **6** (See below)	pGAlmTy1-*Ty1RT-ura3(3-5)I-pTEF1* cassette
S. cerevisiae **BY4741Δ*r-g-x trp1::Ty1-Ty1RT-XylA3*I-P2A-XKS1-pTDH3****	Strain **14** (See below)	pGALmTy1-*Ty1RT-XylA3*I*-P2A-*XKS1-pTDH3** cassette
S. cerevisiae **BY4741Δ*r-g-x trp1::Ty1-Ty1RT-XylAI-P2A-XKS1-pTDH3****	Strain **14** (See below)	pGALmTy1-*Ty1RT-XylAI*-P2A-*XKS1-pTDH3** cassette
S. cerevisiae **BY4741Δ*r-g-x***	*S. cerevisiae* BY4741 Δ*rrm3*	*GRE3*KO+*XKS1* cassette
S. cerevisiae **BY4741 Δ*rrm3 trp1::Ty1-URA3I-pTEF1***	*S. cerevisiae* BY4741 Δ*rrm3*	pGAlmTy1-*URA3I-pTEF1* cassette
S. cerevisiae **BY4741Δ*r-g-x trp1::Ty1-Ty1RT-XylA3*-P2A-XKS1-pTDH3****	Strain **14** (See below)	pGALmTy1-*Ty1RT-XylA3**-P2A-*XKS1-pTDH3** cassette
S. cerevisiae **BY4741Δ*r-g-x trp1::Ty1-Ty1RT-xyla3xks1-66***	*S. cerevisiae* BY4741Δ*r-g-x trp1::Ty1-Ty1RT-XylA3*-P2A-XKS1-pTDH3**	
S. cerevisiae **BY4741Δ*r-g-x trp1::Ty1-Ty1RT-xyla3xks1-20***	*S. cerevisiae* BY4741Δ*r-g-x trp1::Ty1-Ty1RT-XylA3*-P2A-XKS1-pTDH3**	
S. cerevisiae **BY4741Δ*r-g-x trp1::Ty1-Ty1RT-XylA-P2A-XKS1-pTDH3****	Strain **14** (See below)	pGALmTy1-*Ty1RT-XylA*-P2A-*XKS1-pTDH3** cassette
S. cerevisiae **BY4741Δ*r-g-x trp1::Ty1-Ty1RT-xylaxks1-34***	*S. cerevisiae* BY4741Δ*r-g-x trp1::Ty1-Ty1RT-XylA-P2A-XKS1-pTDH3**	
S. cerevisiae **BY4741 Δ*apl2* Δ*mre11***	*S. cerevisiae* BY4741 Δ*apl2* Clean	*MRE11* cassette
S. cerevisiae **BY4741 Δ*mre11* Clean**	Strain **54** (See below)	
S. cerevisiae **BY4741 Δ*hir3* Clean**	Strain **55** (See below)	
S. cerevisiae **BY4741 Δ*apl2* Clean**	Strain **56** (See below)	
S. cerevisiae **BY4741 Δ*mre11-2***	*S. cerevisiae* BY4741	*MRE11* cassette
S. cerevisiae **BY4741 Δ*hir3-2***	*S. cerevisiae* BY4741	*HIR3* cassette
S. cerevisiae **BY4741 Δ*apl2-2***	*S. cerevisiae* BY4741	*APL2* cassette
S. cerevisiae **BY4741 Δ*hir3*Δ*cac3***	*S. cerevisiae* BY4741 Δ*hir3* Clean	*CAC3* cassette
S. cerevisiae **BY4741 Δ*hir3*Δ*apl2***	*S. cerevisiae* BY4741 Δ*apl2* Clean	*HIR3* cassette
S. cerevisiae **BY4741 Δ*hir3*Δ*mre11***	*S. cerevisiae* BY4741 Δ*mre11* clean	*HIR3* cassette

S. cerevisiae* BY4741 Δ*hir3*Δ*cac2	*S. cerevisiae* BY4741 Δ*hir3* Clean	*CAC2* cassette
S. cerevisiae* BY4741 *trp1::dKanMX-pTEF1	Strain **6** (See below)	d*KanMX-pTEF1* cassette
S. cerevisiae* BY4741 *trp1::Ty1-Ty1RT-dKanMX-pTEF1	Strain **6** (See below)	pGALmTy1-*Ty1RT*-d*KanMX-pTEF1* cassette
K. lactis* CBS 2359 Δ*KlURA3	*K. lactis* CBS 2359	*KlURA3*_US-Linker-*KlURA3*_DS
K. lactis* CBS 2359 Δ*KlURA3 KlNDT80::TART-pTEF1-KlURA3AI	*K. lactis* CBS 2359 Δ*KlURA3*	*NDT80*_US-TART-*pTEF1-KlURA3AI-KanMX-NDT80*_DS

Supplementary Table 12: Strains generated through plasmid transformation.

Strain No.	Plasmid 1	Host	Growth Medium
**1**	pGALmTy1-*Ty1RT-URA3I-pHIS3*	*S. cerevisiae* CEN.PK2	YSC −His
**2**	pGALmTy1-*Ty1RT-URA3I-pHIS3*	*S. cerevisiae* BY4741	YSC −His
**3**	pGALmTy1-*Ty1RT-URA3I-pTEF1*	*S. cerevisiae* BY4741	YSC −His
**4**	pGALmTy1-*Ty1RT-URA3I-pTEF1*	*S. cerevisiae* BY4741 Δ*rrm3*	YSC −His
**5**	None	*S. cerevisiae* BY4741 Δ*rrm3 trp1::Ty1-Ty1RT-URA3I-pTEF1*	YSC −His
**6**	p424-*pIMT4*-tRNA^{iMet}-*IMT4t*	*S. cerevisiae* BY4741 Δ*rrm3 trp1::Ty1-Ty1RT-URA3I-pTEF1*	YSC −His −Trp
**7**	p413-*pTEF1*	*S. cerevisiae* BY4741 Δ*rrm3*	YSC −His
**8**	p413-*pTEF1-SPT15*	*S. cerevisiae* BY4741 Δ*rrm3*	YSC −His
**9**	p413-*spt15-B6*	*S. cerevisiae* BY4741 Δ*rrm3*	YSC −His
**10**	p413-*spt15-B6-1*	*S. cerevisiae* BY4741 Δ*rrm3*	YSC −His
**11**	p413-*pTEF1-URA3*	*S. cerevisiae* BY4741 Δ*rrm3*	YSC −His
**12**	p413-*ura3(3-5)*	*S. cerevisiae* BY4741 Δ*rrm3*	YSC −His
**13**	p413-*ura3(3-5-2)*	*S. cerevisiae* BY4741 Δ*rrm3*	YSC −His
**14**	p424-*pIMT4*-tRNA^{iMet}-*IMT4t-TAL1*	*S. cerevisiae* BY4741Δ*r-g-x*	YSC −His −Trp
**15**	p424-*pIMT4*-tRNA^{iMet}-*IMT4t-TAL1*	*S. cerevisiae* BY4741Δ*r-g-x trp1::Ty1-Ty1RT-XylA3*-P2A-XKS1-pTDH3**	YSC −His −Trp
**16**	p424-*pIMT4*-tRNA^{iMet}-*IMT4t-TAL1*	*S. cerevisiae* BY4741Δ*r-g-x trp1::Ty1-Ty1RT-xyla3xks1-66*	YSC −His −Trp
**17**	p424-*pIMT4*-tRNA^{iMet}-*IMT4t-TAL1*	*S. cerevisiae* BY4741Δ*r-g-x trp1::Ty1-Ty1RT-xyla3xks1-20*	YSC −His −Trp
**18**	p424-*pIMT4*-tRNA^{iMet}-*IMT4t-TAL1*	*S. cerevisiae* BY4741Δ*r-g-x trp1::Ty1-Ty1RT-XylA-P2A-XKS1-pTDH3**	YSC −His −Trp
**19**	p424-*pIMT4*-tRNA^{iMet}-*IMT4t-TAL1*	*S. cerevisiae* BY4741Δ*r-g-x trp1::Ty1-Ty1RT-xylaxks1-34*	YSC −His −Trp
**20**	p413-*pGAL1-YFP*	*S. cerevisiae* YSX3	YSC −His
**21**	pGALmTy1-*Ty1RT-URA3I-pHIS3*	*S. cerevisiae* BY4741 Δ*rrm3*	YSC −His
**22**	pGALmTy1-*Ty1RT-eGFP-URA3I-pHIS3*	*S. cerevisiae* BY4741 Δ*rrm3*	YSC −His
**23**	pGALmTy1-*Ty1RT-CAN1-URA3I-pHIS3*	*S. cerevisiae* BY4741 Δ*rrm3*	YSC −His
**24**	pGALmTy1-*Ty1RT-LacZ-URA3I-pHIS3*	*S. cerevisiae* BY4741 Δ*rrm3*	YSC −His
**25**	pGALmTy1-*Ty1RT-LacZ-eGFP-URA3I-pHIS3*	*S. cerevisiae* BY4741 Δ*rrm3*	YSC −His
**26**	pGALmTy1-*Ty1RT-LacZ-YFP-mStrawberry-URA3I-pHIS3*	*S. cerevisiae* BY4741 Δ*rrm3*	YSC −His
**27**	p424-*pIMT4*-tRNA^{iMet}-*IMT4t*	*S. cerevisiae* BY4741 Δ*rrm3 trp1::Ty1-URA3I-pTEF1*	YSC −His −Trp
**28**	pGALmTy1-*URA3I-pTEF1*	*S. cerevisiae* BY4741	YSC −His
**29**	p416-*pCYC1-YFP*	*S. cerevisiae* BY4741	YSC −Ura
**30**	p416-*pTEF1-YFP*	*S. cerevisiae* BY4741	YSC −Ura
**31**	p416-*pTDH3-YFP*	*S. cerevisiae* BY4741	YSC −Ura
**32**	pGALmTy1-*Ty1RT-URA3I-pCYC1*	*S. cerevisiae* BY4741	YSC −His
**33**	pGALmTy1-*Ty1RT-URA3I-pTDH3*	*S. cerevisiae* BY4741	YSC −His
**34**	pGALmTy1-*Ty1RT-URA3I-pHIS3*	*S. cerevisiae* BY4741 Δ*apl2* Δ*mre11*	YSC −His
**35**	pGALmTy1-*Ty1RT-URA3I-pHIS3*	*S. cerevisiae* BY4741 Δ*ice2*	YSC −His
**36**	pGALmTy1-*Ty1RT-URA3I-pHIS3*	*S. cerevisiae* BY4741 Δ*ckb2*	YSC −His

**37**	pGALmTy1-*Ty1RT-URA3I-pHIS3*	*S. cerevisiae* BY4741 Δ*hir3*	YSC −His
**38**	pGALmTy1-*Ty1RT-URA3I-pHIS3*	*S. cerevisiae* BY4741 Δ*apl2*	YSC −His
**39**	pGALmTy1-*Ty1RT-URA3I-pHIS3*	*S. cerevisiae* BY4741 Δ*hir3*Δ*cac3*	YSC −His
**40**	pGALmTy1-*Ty1RT-URA3I-pHIS3*	*S. cerevisiae* BY4741 Δ*mrc1*	YSC −His
**41**	pGALmTy1-*Ty1RT-URA3I-pHIS3*	*S. cerevisiae* BY4741 Δ*hir3*Δ*apl2*	YSC −His
**42**	pGALmTy1-*Ty1RT-URA3I-pHIS3*	*S. cerevisiae* BY4741 Δ*hir3*Δ*mre11*	YSC −His
**43**	pGALmTy1-*Ty1RT-URA3I-pHIS3*	*S. cerevisiae* BY4741 Δ*cac2*	YSC −His
**44**	pGALmTy1-*Ty1RT-URA3I-pHIS3*	*S. cerevisiae* BY4741 Δ*mre11*	YSC −His
**45**	pGALmTy1-*Ty1RT-URA3I-pHIS3*	*S. cerevisiae* BY4741 Δ*hir3*Δ*cac2*	YSC −His
**46**	pGALmTy1-*Ty1RT-URA3I-pHIS3*	*S. cerevisiae* BY4741 Δ*cac3*	YSC −His
**47**	p414-*pTDH3*	*S. cerevisiae* BY4741 Δ*rrm3* *trp1::Ty1-Ty1RT-URA3I-pTEF1*	YSC −His −Trp
**48**	p414-*pSUP4*-tRNA^{iMet}-*RPR1t*	*S. cerevisiae* BY4741 Δ*rrm3* *trp1::Ty1-Ty1RT-URA3I-pTEF1*	YSC −His −Trp
**49**	p414-*pSUP4*-tRNA^{iMet}-*IMT4t*	*S. cerevisiae* BY4741 Δ*rrm3* *trp1::Ty1-Ty1RT-URA3I-pTEF1*	YSC −His −Trp
**50**	p414-*pIMT4*-tRNA^{iMet}-*IMT4t*	*S. cerevisiae* BY4741 Δ*rrm3* *trp1::Ty1-Ty1RT-URA3I-pTEF1*	YSC −His −Trp
**51**	p424-*pTDH3*	*S. cerevisiae* BY4741 Δ*rrm3* *trp1::Ty1-Ty1RT-URA3I-pTEF1*	YSC −His −Trp
**52**	P424- *pSUP4*-tRNA^{iMet}-*RPR1t*	*S. cerevisiae* BY4741 Δ*rrm3* *trp1::Ty1-Ty1RT-URA3I-pTEF1*	YSC −His −Trp
**53**	P424- *pSUP4*-tRNA^{iMet}-*IMT4t*	*S. cerevisiae* BY4741 Δ*rrm3* *trp1::Ty1-Ty1RT-URA3I-pTEF1*	YSC −His −Trp
**54**	pSH47	*S. cerevisiae* BY4741 Δ*mre11-2*	YSC −Ura
**55**	pSH47	*S. cerevisiae* BY4741 Δ*hir3-2*	YSC −Ura
**56**	pSH47	*S. cerevisiae* BY4741 Δ*apl2-2*	YSC −Ura
**57**	P424-*pIMT4*-tRNA^{iMet}-*IMT4t*	*S. cerevisiae* BY4741Δ*r-g-x* *trp1::Ty1-Ty1RT-XylA3*I-P2A-XKS1-pTDH3**	YSC −His
**58**	P424-*pIMT4*-tRNA^{iMet}-*IMT4t*	*S. cerevisiae* BY4741Δ*r-g-x* *trp1::Ty1-Ty1RT-XylAI-P2A-XKS1-pTDH3**	YSC −His
**59**	p416-*pGAL1-CAS9-CYC1t-TRP1*gRNA	*S. cerevisiae* BY4741 Δ*rrm3*	YSC −Ura
**60**	p416-*pGAL1-CAS9-CYC1t-TRP1*gRNA	*S. cerevisiae* BY4741Δ*r-g-x*	YSC −Ura
**61**	pGALmTy1-*Ty1RT-URA3I-pTEF1-Tsynth1*	*S. cerevisiae* BY4741	YSC −His
**62**	pGALmTy1-*Ty1RT-URA3I-pTEF1-Tsynth5*	*S. cerevisiae* BY4741	YSC −His
**63**	pGALmTy1-*Ty1RT-URA3I-pTEF1-Tsynth8*	*S. cerevisiae* BY4741	YSC −His
**64**	pGALmTy1-*Ty1RT-URA3I-pTEF1-CYC1t*	*S. cerevisiae* BY4741	YSC −His
**65**	pGALmTy1-*Ty1RT-URA3I-pTEF1-IDP1t*	*S. cerevisiae* BY4741	YSC −His
**66**	pGALmTy1-*Ty1RT-URA3I-pTEF1-PRM9t*	*S. cerevisiae* BY4741	YSC −His
**67**	P424-*pIMT4*-tRNA^{iMet}-*IMT4t*	*S. cerevisiae* BY4741 *trp1::dKanMX-pTEF1*	YSC −His −Trp
**68**	P424-*pIMT4*-tRNA^{iMet}-*IMT4t*	*S. cerevisiae* BY4741 *trp1::Ty1-Ty1RT-dKanMX-pTEF1*	YSC −His −Trp
**69**	p416-*pGAL1-CAS9-CYC1t-TRP1*gRNA	*S. cerevisiae* BY4741	YSC −Ura
**70**	None	*K. lactis* CBS 2359 Δ*KlURA3* *KlNDT80::TART-pTEF1-KlURA3AI*	YSC

Supplementary Table 13: Strains used in experiments described in this study.

Figure	Category	Series	Strain No.
**Figure 2b: Iterative improvement of synthetic Ty1 transposition rate**	BY4741 *Ty1RT*: *pHIS3-URA3I*		2
	BY4741 *Ty1RT*: *pTEF1-URA3I*		3
	BY4741 Δ*rrm3 Ty1RT*: *pTEF1-URA3I*		4
	BY4741 Δ*rrm3 Ty1RT*: *pTEF1-URA3I*		5
	BY4741 Δ*rrm3 Ty1RT*: *pTEF1-URA3I*, High OD		5
	BY4741 Δ*rrm3 Ty1RT*: *pTEF1-URA3I*, 22°C induction, High OD		5
	BY4741 Δ*rrm3 Ty1RT*: *pTEF1-URA3I*, 22°C induction, High OD, $tRNA^{iMet}$ overexpression		6
**Figure 3a: Substitution of alternative promoters in retroelement**	BY4741 *Ty1RT*		2
	BY4741 *Ty1RT pCYC1*		32
	BY4741 *Ty1RT pTEF1*		3
	BY4741 *Ty1RT pTDH3*		33
**Figure 3b: Transposition rates for BY4741 knockout strains**	Wild type		2
	Δ*apl2* Δ*mre11*		34
	Δ*ice2*		35
	Δ*ckb2*		36
	Δ*hir3*		37
	Δ*apl2*		38
	Δ*hir3*Δ*cac3*		39
	Δ*mrc1*		40
	Δ*hir3*Δ*apl2*		41
	Δ*hir3*Δ*mre11*		42
	Δ*cac2*		43
	Δ*mre11*		44
	Δ*hir3*Δ*cac2*		45
	Δ*cac3*		46
	Δ*rrm3*		21
**Figure 3c: Transcript levels of engineered retroelements**	Transposition Repressed	*Ty1RT* WT	3
		Ty1RT Δ*rrm3*	4
		No RT	28
	Transposition Induced	*Ty1RT* WT	3
		Ty1RT Δ*rrm3*	4
		No RT	28
**Figure 3d: cDNA levels of engineered retroelements**	Transposition Repressed	*Ty1RT* WT	3
		Ty1RT Δ*rrm3*	4
		No RT	28
	Transposition Induced	*Ty1RT* WT	3
		Ty1RT Δ*rrm3*	4
		No RT	28
**Figure 3e: Transposition rate of strains overexpressing the**	Low copy	no tRNA overexpression	47
		pSUP4-$tRNA^{iMet}$-*RPR1t*	48

**initiator methionine tRNA IMT4**		*pSUP4*-tRNA^{iMet}-*IMT4t*	49
		pIMT4-tRNA^{iMet}-*IMT4t*	50
	High copy	no tRNA overexpression	51
		pSUP4-tRNA^{iMet}-*RPR1t*	52
		pSUP4-tRNA^{iMet}-*IMT4t*	53
		pIMT4-tRNA^{iMet}-*IMT4t*	6
**Figure 3f: tRNA^{iMet} overexpression improves cDNA synthesis**	Low copy	No tRNA Glc	47
		No tRNA Gal	47
		With tRNA Glc	50
		With tRNA Gal	50
	High copy	No tRNA Glc	51
		No tRNA Gal	51
		With tRNA Glc	6
		With tRNA Gal	6
**Figure 3g: Transposition rate of retroelements containing cargo of various sizes**	3 Day Induction	1135 bp	21
		1890 bp	22
		2907 bp	23
		4219 bp	24
		4964 bp	25
		5768 bp	26
	5 Day Induction	1135 bp	21
		1890 bp	22
		2907 bp	23
		4219 bp	24
		4964 bp	25
		5768 bp	26
	7 Day Induction	1135 bp	21
		1890 bp	22
		2907 bp	23
		4219 bp	24
		4964 bp	25
		5768 bp	26
**Figure 4a: dKanMX reversion mutation assay**	d*KanMX* in genome		67
	d*KanMX* in ICE retroelement		68
**Figure 4b: Mutation distribution of Ty1RT**	*URA3* with RT		3
	Amp without RT		3
**Figure 4c: Mutation frequencies of Ty1RT**	*URA3* with RT		3
	Amp without RT		3
**Figure 5: Implementation of ICE for improvement of three distinct classes of genetic cargo**	a: ICE of *URA3*	WT: *URA3*	11
		Mutant: *ura3(3-5)*	12
		Mutant: *ura3(3-5-2)*	13
	b: ICE of *SPT15*	WT: *SPT15*	8
		Mutant: *spt15-B6*	9
		Mutant: *spt15-B6-1*	10
	c: ICE of xylose isomerase pathway	WT: *IK* pathway	18
		Mutant: *IK*-34	19
	d: ICE of xylose isomerase pathway	WT: *I3K* pathway	15
		Mutant: *I3K*-66	16
		Mutant: *I3K*-20	17
**Figure 7: Transposition rates for alternate yeast strains**	BY4741		2
	CEN.PK2		1

	K. lactis	70
**Supplementary Fig. 1a: Promoters used in this study**	*pCYC1*	29
	pTEF1	30
	pTDH3	31
**Supplementary Fig. 1b: Transposition of RT-containing and RT-less retroelements at 22ºC**	BY4741 Δ*rrm3 Ty1RT*: *TEF1p-URA3I*, 22 °C induction, High OD	6
	BY4741 Δ*rrm3* NoRT: *TEF1p-URA3I*, 22 °C induction, High OD	27
**Supplementary Fig. 1c: Effect of terminators on rate of transposition**	No Terminator	3
	Tsynth1	61
	Tsynth5	62
	Tsynth8	63
	CYC1t	64
	IDP1t	65
	PRM9t	66
**Supplementary Fig. 3a: Transcriptional rates of *SPT15* mutants**	WT: *SPT15*	8
	Mutant: *Spt15-B6-1*	10
**Supplementary Fig. 3b: Growth of *ura3(3-5)*-containing strains in media lacking uracil and containing 5-FOA**	0 g L^{-1}	12
	0.3 g L^{-1}	12
	0.5 g L^{-1}	12
**Supplementary Fig. 3c: Activity of *Gal1p* in xylose-containing media**	Glu (grown in glucose)	20
	Xyl (grown in glucose)	20
	Gal (grown in galactose)	20
	Xyl (grown in galactose)	20
**Supplementary Fig. 3d: Growth test of *xylA* pathway mutants**	Δ*r-g-x* blank strain	14
	WT: *IK* pathway	18
	Mutant: *IK-34*	19
**Supplementary Fig. 3e: Growth test of *xylA3** pathway mutants**	Δ*r-g-x* blank strain	14
	WT: *I3K* pathway	15
	Mutant: *I3K-66*	16
	Mutant: *I3K-20*	17
**Supplementary Fig. 3f-i: Growth test of *SPT15* variants**	WT: *SPT15*	8
	Mutant: *SPT15-B6*	9
	Mutant: *SPT15-B6-1*	10

Supplementary Table 14: Genes Synthesized.

Name	Sequence	Source
**Synthetic *HIVRT***	ATGGCTTTGAAGGCTGTTCCAATTTCTCCAATTGAAACCGT<br>TCCAGTTAAGTTGAAACCAGGTATGGATGGTCCAAAAGTT<br>AAGCAATGGCCTTTGACCGAAGAAAAGATTAAGGCTTTGG<br>TTGAAATCTGCACCGAAATGGAAAAAGAAGGTAAGATCTC<br>TAAGATCGGTCCAGAAAATCCATACAACACTCCAGTTTTCG<br>CCATCAAGAAAAAGGATTCTACTAAGTGGAGAAAGTTGGT<br>TGACTTCAGAGAATTGAACAAGAGAACCCAAGATTTCTGG<br>GAAGTTCAATTGGGTATTCCACATCCAGCTGGTTTGAAGAA<br>GAAAAAGTCTGTTACCGTTATGGATGTTGGTGATGCTTACT<br>TTTCTGTTCCATTGGACGAAGATTTCAGAAAGTACACTGCT<br>TTCACCATCCCATCCATTAACAACGAAACTCCAGGTATCAG<br>ATACCAATACAACGTTTTGCCACAAGGTTGGAAAGGTTCTC<br>CAGCTATTTTTCAATCTTCCATGACCAAGATTTTGGAACCA<br>TTCAAGAAGCAAAACCCAGACATCGTCATCTACCAATATAT<br>GGATGACTTGTACGTTGGTTCCGATTTGGAAATTGGTCAAC<br>ACAGAACTAAGATCGAAGAATTGAGACAACACTTGTTGAG<br>ATGGGGTTTGACTACTCCAGATAAGAAGCACCAAAAAGAA<br>CCACCATTTTTGTGGATGGGTTACGAATTGCATCCTGATAA<br>GTGGACTGTTCAACCTATAGTTTTGCCAGAAAAAGACTCTT<br>GGACCGTTAACGACATCCAAAAATTGGTCGGTAAATTGAA<br>CTGGGCCTCTCAAATCTATCCAGGTATTAAGGTTAGACAAT<br>TGTGCAAGTTGTTGAGAGGTACAAAGGCTTTGACTGAAGTT<br>ATTCCATTGACAGAAGAAGCCGAATTGGAATTGGCTGAAA<br>ACAGAGAAATTTTGAAAGAACCAGTTCACGGTGTTTACTAC<br>GATCCATCTAAAGATTTGATCGCCGAAATCCAAAAACAAG<br>GTCAAGGTCAATGGACCTACCAAATCTACCAAGAACCTTTT<br>AAGAACTTGAAAACCGGTAAGTACGCTAGAATGAGAGGTG<br>CTCATACAAACGATGTCAAGCAATTGACTGAAGCCGTTCA<br>AAAGATTACCACCGAATCTATAGTTATCTGGGGTAAGACTC<br>CAAAGTTCAAGTTGCCAATTCAAAAAGAAACTTGGGAAAC<br>CTGGTGGACTGAATATTGGCAAGCTACTTGGATTCCAGAAT<br>GGGAATTTGTTAATACTCCACCATTGGTCAAGTTGTGGTAT<br>CAATTGGAAAAAGAACCTATCGTTGGTGCCGAAACTTTTTA<br>TGTTGATGGTGCTGCTAACATGGAAACTAAGTTGGGTAAA<br>GCTGGTTACGTTACCAACAAAGGTAGACAAAAAGTTGTCC<br>CATTGACCAACACTACCAATCAAAAGACTGAATTGCAAGC<br>TATCTACTTGGCCTTGCAAGATTCTGGTTTGGAAGTTAACA<br>TCGTTACCGATTCTCAATACGCCTTGGGTATTATTCAAGCT<br>CAACCAGATAAGTCCGAATCCGAATTGGTTAATCAAATCAT<br>CGAACAATTGATCAAGAAAGAAAAGGTTTACTTGGCTTGG<br>GTTCCAGCTCATAAGGGTATTGGTGGTAATGAACAAGTTGA<br>CAAGTTGGTTTCTGCCGGTATTAGAAAGATCTTGTAA	Life Technologies
**Synthetic Ty1**	CTCGAGGAGAACTTCTAGTATATTCTGTATACCTAATATTA<br>TAGCCTTTATCAACAGATATCTGGTAGCGCCTGTGCTTCGG<br>TTACTTCTAAGGAAGTCCACACAAATCAAGATCCGTTAGAC<br>GTTTCAGCTTCCAAAACAGAAGATTGTGAGAAGGCTTCCAC<br>TATCGATGCATGCGAATTCTTAATTAATATTACATTATGGG<br>TGGTGGCGCGCCTGTTGGAATAGAAATCAACTATCATCTAC<br>TAACTAGTATTTACATTACTAGTATATTATCATATACGGTG<br>TTAGAAGATGACGCAAATGATGAGAAATAGTCATCTAAAT<br>TAGTGGAAGCTGAAACGCAAGGATTGATAATGTAATAGGA<br>TCAATGAATATAAACATATAAAATGATGATAATAATATTTA	Life Technologies

	TAGAATTGTGTAGAATTGCAGATTCCCTTTTATGGATTCCT<br>AAATCCTTGAGGAGAACTTCTAGTATATTCTGTATACCTAA<br>TATTATAGCCTTTATCAACAATGGAATCCCAACAATTATCT<br>CAACATTCACCCATTTCTCA	
**Synthetic *Ty1RT***	ATGAATAATTCATCGCACAATATTGTTCCTATCAAAACGCC<br>AACTACTGTTTCTGAACAGAATACCGAGGAATCTATCATCG<br>CTGATCTCCCACTCCCTGATCTACCTCCAGAATCTCCTACC<br>GAATTTCCTGACCCATTTAAAGAACTCCCACCGATAAATTC<br>TCGTCAAACTAATTCCAGTTTGGGTGGTATTGGTGACTCTA<br>ATGCCTATACTACTATCAACAGTAAGAAAAGATCATTAGA<br>AGATAATGAAACTGAAATTAAGGTATCACGAGACACATGG<br>AATACTAAGAATATGCGTAGTTTAGAACCTCCGAGATCGA<br>AGAAACGTATTCACCTGATTGCAGCTGTAAAAGCAGTAAA<br>ATCAATCAAACCAATACGGACAACCTTACGATACGATGAG<br>GCAATCACCTATAATAAAGATATTAAAGAAAAAGAAAAAT<br>ATATCGAGGCATACCACAAAGAAGTCAATCAACTGTTGAA<br>GATGAAAACTTGGGACACTGACGAATATTATGACAGAAAA<br>GAAATAGACCCTAAAAGAGTAATAAACTCAATGTTTATCTT<br>CAACAAGAAACGTGACGGTACTCATAAAGCTAGATTTGTT<br>GCAAGAGGTGATATTCAGCATCCTGACACTTACGACTCAG<br>GTATGCAATCCAATACCGTACATCACTATGCATTAATGACA<br>TCCCTGTCACTTGCATTAGACAATAACTACTATATTACACA<br>ATTAGACATATCTTCGGCATATTTGTATGCAGACATCAAAG<br>AAGAATTATACATAAGACCTCCACCACATTTAGGAATGAA<br>TGATAAGTTGATACGTTTGAAGAAATCACTTTATGGATTGA<br>AACAAAGTGGAGCGAACTGGTACGAAACTATCAAATCATA<br>CCTGATACAACAATGTGGTATGGAAGAAGTTCGTGGATGG<br>TCATGCGTATTTAAAAACAGTCAAGTGACAATTTGTTTATT<br>CGTAGATGATATGGTATTGTTTAGCAAAAATCTAAATTCAA<br>ACAAAAGAATTATAGAGAAGCTTAAGATGCAATACGACAC<br>CAAGATTATAAATCTAGGCGAAAGTGATGAGGAAATTCAA<br>TATGACATACTTGGCTTAGAAATCAAATATCAAAGAGGTA<br>AATACATGAAATTAGGTATGGAAAACTCATTAACTGAGAA<br>AATACCCAAATTAAACGTACCTTTGAATCCAAAAGGAAGA<br>AAACTTAGCGCTCCAGGTCAACCAGGTCTTTATATAGACCA<br>GGATGAACTAGAAATAGATGAAGATGAATACAAAGAGAA<br>GGTACATGAAATGCAAAAGTTGATTGGTCTAGCTTCATATG<br>TTGGATATAAATTTAGATTTGACTTACTATACTACATCAAC<br>ACACTTGCTCAACATATACTATTCCCCTCTAGGCAAGTTTT<br>AGACATGACATATGAGTTGATACAATTCATGTGGGACACT<br>AGAGATAAACAACTGATATGGCACAAAAACAAACCTACCG<br>AGCCAGATAATAAACTAGTCGCAATAAGTGATGCTTCGTAT<br>GGCAACCAACCGTATTATAAATCACAAATTGGCAACATAT<br>ATTTACTTAATGGAAAGGTAATTGGAGGAAAGTCCACCAA<br>GGCTTCATTAACATGTACTTCAACTACGGAAGCAGAAATAC<br>ACGCGATAAGTGAATCTGTCCCATTATTAAATAATCTAAGT<br>TACCTGATACAAGAACTTAACAAGAAACCAATTATTAAAG<br>GCTTACTTACTGATAGTAGATCAACGATCAGTATAATTAAG<br>TCTACAAATGAAGAGAAATTTAGAAACAGATTTTTTGGCAC<br>AAAGGCAATGAGACTTAGAGATGAAGTATCAGGTAATAAT<br>TTATACGTATACTACATCGAGACCAAGAAGAACATTGCTG<br>ATGTGATGACAAAACCTCTTCCGATAAAAACATTTAAACTA<br>TTAACTAACAAATGGATTCATTAG	Life Technologies
***pIMT4*-tRNA^{iMet}-*IMT4t* (gblock)**	CCGGAGCTCAACATCCAGTTCTTTCAAGTTGGCTAAGCGCC<br>GTGGCGCAGTGGAAGCGCGCAGGGCTCATAACCCTGATGT<br>CCTCGGATCGAAACCGAGCGGCGCTAATTTTTCATTTCTTT	IDT

	TTGCCCGCAACAATAACACAGATCGCAATCATTTAAGCTAT AGACTATAAGGTACCGCC	
***pSUP4*-tRNA^{iMet}-*IMT4t* (gblock)**	CCGGAGCTCACCATCTTGGAAGGACCGGATAATTATTTGAA ATCTCTTTTTCAATTGTATATGTGTTATGTAGTATACTCTTT CTTCAACAGCGCCGTGGCGCAGTGGAAGCGCGCAGGGCTC ATAACCCTGATGTCCTCGGATCGAAACCGAGCGGCGCTAA TTTTTCATTTCTTTTTGCCCGCAACAATAACACAGATCGCA ATCATTTAAGCTATAGACTATAAGGTACCGCC	IDT
***pSUP4*-tRNA^{iMet}-*RPR1t* (gblock)**	CCGGAGCTCACCATCTTGGAAGGACCGGATAATTATTTGAA ATCTCTTTTTCAATTGTATATGTGTTATGTAGTATACTCTTT CTTCAACAGCGCCGTGGCGCAGTGGAAGCGCGCAGGGCTC ATAACCCTGATGTCCTCGGATCGAAACCGAGCGGCGCTATC TTTCTTTTTTAATTTCACTTATTTGCGGGTACCGCC	IDT
***URA3*gRNA (gblock)**	TGTACGCATGTAACATTATACTGAAAACCTTGCTTGAGAAG GTTTTGGGACGCTCGAGACATAAAAAACAAAAAAAGCACC ACCGACTCGGTGCCACTTTTTCAAGTTGATAACGGACTAGC CTTATTTTAACTTGCTATTTCTAGCTCTAAAACCACCGTGTG CATTCGTAATGGATCATTTATCTTTCACTGCGGAGAAGTTT CGAACGCCGAAACATGCGCACCAACTTTCACTTCTACAGCG TTTGACCAAAATCTTTTGAACAGAACATTGTAGGGTGTGAA AAAATGCGCACCTTTACCGCTAGCCCAAGAGGGCACTACA AAATCTAGAGTTGTACTTCAAACGTACATGTAATCACCTTG TATATACTCGAAAGAAAACATCAAGTTTCTGTATAAATATG AGTGAAAGCATAATCATACATTATCTTTTCAAAGGGTACCC AATTCGCCCTATAGTGAGTCGTATTACGCGCGCTCACTGGC CGTCGTTTT	IDT
***TRP1*gRNA (gblock)**	AGACATAAAAAACAAAAAAAGCACCACCGACTCGGTGCCA CTTTTTCAAGTTGATAACGGACTAGCCTTATTTTAACTTGCT ATTTCTAGCTCTAAAACAGGAACTCTTGGTATTCTTGGATC ATTTATCTTTCACTGCGGAGAAGTTTCGAACGCCGAAACAT GCGCACCAACTTTCACTTCTACAGCGTTTGACCAAAATCTT TTGAACAGAACATTGTAGGGTGTGAAAAAATGCGCACCTT TACCGCTAGCCCAAGAGGGCACTACAAAATCTAGAGTTGT ACTTCAAACGTACATGTAATCACCTTGTATATACTCGAAAG AAAACATCAAGTTTCTGTATAAATATGAGTGAAAGCATAA TCATACATTATCTTTTCAAAG	IDT
***(m)GAXnewintron* (gblock)**	TATTTCCTCAAGTGACGGGATGCCGCTATTGAGTATGCACA TCCCTGCGTCTACAGCGCAAGCCAACTCATGCTCGAACGTA TGACCGGCTAGAGTAGCGTGATTAACTTCAATATTGACTTT GAAGTCTTTATCTAGATTATGAGCTTTCAAGAAACCGATTG CAGTCTCGGTGTCTACGTCGTATTGGTGTTTAGTAGGTTCC ATAGGCTTAGGTTCAATTAGGAAGGTATGTTAATATGGACT AAAGGAGGCTTTTGGCGCGCCGAATTTTTACTAACAAATGG TATTATTTATAACAGGTACCTTTAAAGCCTTTACTACGTGC ATAGTCCCTAGCCATGGTCAACATGGTTGCCATATGTTCTT TTTCTCTTTTTTGATCAGTATTAAGTAATGACATATAACCTT CTCTACCGCCCCAAAACACGTAATTTTCTGCGCCCAATTCG ATGCCTGCGTCGATTGCGTTTTTAATTTGTACAATAGCCCTC GCAACCACGTCAAAATCAGGATTAGTTGAAGCTCCGTTCAT ATACCTTTTATGACCAAATACATTAGCAGTTGACCATAATA ACTTAATCCCAGTTTCCTTCTGTTTTCTTTCAAGTACGCGA CAACTGCCTTCAAGTTGGATTCATATTCCTCAATAGAGTTA CCTTCAGATACTAAATCCACGTCATGAAAACAATAGTAAG GAATACCTAATTTTTGCATTATCTCGAAACCAGCATCAACT TTTTGCTTTGCTATTTCAATTGCGTCTGTACCTTCGTTCCAT	IDT

	GGGAAACTTTTAGTCCCTCCTCCAAATTGATCTGCTCCTTC<br>AGCACACAAGGTGTGCCACCAAGCCATGGCAAATCTTAAC<br>CAGTCTTTCATTTTTTTACCCATGACCTCTTTTTCGGCATCA<br>TAGTAATGAAAGGCCAATGGGTTCTTACTATCTTTACCCTC<br>GAATTTTATCTTTTGAATTTGAGGGAAATATTCTTTAGCCAT<br>cccgggatccgtcgaaactaagttctggCTTCTCAACTTTCAACTCTTTCGC<br>CAACGGAACTGGAGTCGTTAAGC	
(m)GA3Xnewintron **(gblock)**	TGGTAGCACCAGAACCTTGATACATCGCGATAATAGCCTCG<br>TATAGTTCTTGTTTTCCTGAGGTTTGTTTCGGTTCTCCATTC<br>TTCTTTCCATATTCATAGACTTGCTCCAATGTCAGTTTCCCA<br>TCTTCAAAATCTTTTCCTATTCCAGAATCGAATGAAGCATA<br>TCTTTCCTTCTTCATTTTTGTGTATGGAGACTCCTGTAACAA<br>TTTAGCTGCATTCTCCAATGCACGAGCCATGGCATCCATAC<br>CAGACACATGAGCGATAATAATATCTTCTAAGTCAGTCGA<br>ATTACGTCTTGTCTTAGCGTCGAAGTTTGTTCCACCGGTTAC<br>AAAACCGCCACCCCTTATAATTTCCATCCAAGCCTGTACCA<br>ATTCGTACTGGTCGATAGGAAACTGGTCAGTGTCCCAGCCA<br>TTTTGATAGTCACCCCTATTGGCATCTATGCTACCTAACATC<br>CCTGCGTCTACAGCGCAAGCCAACTCATGCTCGAACGTATG<br>ACCGGCTAGAGTAGCGTGATTAACTTCAATATTGACTTTGA<br>AGTCTTTATCTAGATTATGAGCTTTCAAGAAACCGATTGCA<br>GTCTCAGTGTCTACGTCGTATTGGTGTTTAGTAGGTTCCAT<br>AGGCTTAGGTTCAATAAGGAAGGTATGTTAATATGGACTA<br>AAGGAGGCTTTTGGCGCGCCGAATTTTTACTAACAAATGGT<br>ATTATTTATAACAGGTACCTTTAAAGCCTTTACTACGTGCA<br>TAGTCCCTAGCCATGGTCAACATGGTTGCCATATGTTCTTTT<br>TCTCTTTTTTGATCAGTATTAAGTAATGACATATAACCTTCT<br>CTACCGCCCCAAAACACGTAATTTTCTGCGCCCAATTCGAT<br>GCCTGTGTCGATTGCGTTTTTAATTTGTACAATAGCCCTCGC<br>AACCACATCAAAATCAGGATTAGTCGAAGCTCCGTTCATAT<br>ACCTTTTATGACCAAATACATTAGCAGATGACCATAATAAC<br>TTAATCCCAGTTTCCTTCTGTTTATCTTTCAAGTACGCGACA<br>ACTGCCTTCAAGTTGGATTCATATTCCCCAATAGAGTTACC<br>TTCAGATACTAAATCCACGTCATGAAAACAATAGTAAGGA<br>ATACCTAATTTTTGCATTATCTCGAAACCAGCATCAACTTTT<br>TGCTTTGCTATTTCAATTGCGTCTGTACCTTCGTTCCATGGG<br>AAACTTTTAGTCCCTCCTCCAAATTGATCTGCTCCTTCAGCA<br>CACAAGGTGTGCCACCAAGCCATGGCAAATCTTAACCAGT<br>CTTTCATTTTTTTACCCATGACCTCTTTTTCGGCATCATAGT<br>AATGAAAGGCCAATGGGTTCTTGCTATCTTTACCATCGAAT<br>TTTATCTTTTGAATTTGAGGGAAATATTCTTTAGCCATcccgg<br>gatccgtcgaaactaagttctgg	IDT
KlURA3AI **(gBlock)**	ATTCATTAGATCGATAAGCTTGGGCTGCAGCTTTAAATAAT<br>CGGTGTTAATGGGGAGCGCTGATTCTCTTTTGGTACGCTTC<br>CCATCCAGCATTTCTGTATCTTTCACCTTCAACCTTAGGATC<br>TCTACCCTTGGCGAAAAGTCCTCTGCCAACAATGATGATAT<br>CTGATCCACCACTTACAACTTCGTCGACGGTTCTGTACTGC<br>TGACCCAATGCATCGCCTTTGTCGTCTAAACCTACACCTGG<br>GGTCATGATTAGCCAATCAAACCCTTCTTCTCTGGTATGTT<br>AATATGGACTAAAGGAGGCTTTTCTGCAGGTCGACTCTAGA<br>GGATCCCCGGGTACCGAGCTCGAATTTTTACTAACAAATGG<br>TATTATTTATAACAGCCTCCCATATCGTTCTGAGCAATGAA<br>CCCAATAACGAAATCTTTATCACTCTTTGCAATATCAACGG<br>TACCCTTAGTATATTCACCGTGTGCTAGAGAACCCTTGGAA<br>GACAATTCAGCAAGCATCAATAATCCCCTTGGTTCTTTGGT<br>GACCTCTTGCGCACCTTGTTTCAAGCCAGCAACAATACCAG	IDT

CACCAGTAACCCCGTGGGCGTTGGTGATATCAGACCATTCT<br>GCGATACGGTAAACGCCCGATGTATATTGTAATTTGACTGT<br>GTTACCGATATCGGCGAATTTTCTGTCCTCAAATATCAAGA<br>ACTTGTATTTCTCTGCCAATGCTTTCAATGGAACGACAGTA<br>CCCTCATAACTGAAATCATCCAAGATATCAACGTGTGTTTT<br>CAAAAGGCAAATGTATGGACCCAACGTTTCAACAAGTTTC<br>AATAGCTCATCAGTCGAACGAACGTCAAGAGAAGCACACA<br>AATTGGTCTTCTTTTCATCCATTAAACGTAAAAGTTTCGAT<br>GCAACCGGACTTGCATGAGTCTCAGCTCTACTGGTATATGA<br>TTTTGTGGACATAAACTTAGATTAGATTGCTATGCTTTCTTT<br>CTAATGAGCAA

Supplementary Notes

Supplementary Note 1: Analysis of *in vivo* mutation rate through next-generation sequencing

In order to gain a detailed picture of the mutational rate and spectrum enabled by Ty1, yeast cells containing *URA3*-containing, Ty1 retrotransposons were induced in galactose for three days, after which intron-less *URA3* amplicons were generated via PCR of total DNA. As a negative control encompassing background genetic drift, PCR error rate, and sequencing error, a region of the Ampicillin resistance gene (*Amp*) of the same length (which is not reverse transcribed) was also amplified (See Methods). These amplicons were then sequenced (Supplementary Fig. 5d). Analysis of identified mutants showed a uniform error distribution across each amplicon, with *URA3* consistently showing a higher mutation rate (0.28 kb^{-1}) than *Amp* (0.13 kb^{-1}) (Fig. 4b). This 0.15 kb^{-1} increase in error rate above the combined effects of drift, PCR error, and sequencing error was thus due to Ty1 and was also reflected in an increased frequency of observing a given number of mutations per 200bp read in *URA3* versus *Amp* (Fig. 4c). Finally, Ty1 exhibited a mutational spectrum commensurate with other commonly-used error-prone polymerases and displayed an error rate which is useful for directed evolution of genes and pathways (Table 1). Collectively, this analysis indicated that the Ty1 retrotransposon is a useful vehicle for introducing mutations to defined genes and pathways *in vivo*.

Supplementary Note 2: Analysis of *in vivo* mutation rate using dKanMX reversion assay

In order to directly compare the rates of Ty1-induced mutations and background genetic drift, the d*KanMX* marker was constructed for use in a mutation reversion assay, containing two point mutations that together prevent functional activity. The first is T405A, which introduces an artificial stop codon at the 135th residue, in the middle of the proposed active site[18].

Interestingly, this mutation alone does not inactivate *KanMX*, and expression of this variant confers G418 resistance. The second mutation, T530A, is a missense variant resulting in Trp177Leu. Together, these mutations inactivate *KanMX*, and do not allow growth in the presence of G418.

To separate the effect of background genetic drift absent the optimized Ty1 retroelement, d*KanMX* was either integrated directly into the genome (g-dKanMX) or incorporated into a Ty1 mutagenesis cassette, which was also integrated into the genome (ICE-dKanMX). Both strains were grown to stationary phase and exposed to galactose, then plated on media containing G418. The rate of reversion mutations could then be measured by counting G418-resistant colonies. Genomic DNA from 39 colonies of both strains was extracted, a PCR designed to amplify position 135 of d*KanMX* was performed, and amplicons were sequenced via Sanger sequencing. In 34 of 39 resistant colonies sequenced, mutations at position 135 reverted the artificial stop codon to one of several non-stop codons, thus activating the gene and conferring a G418-resistant phenotype. We hypothesize that the 5 remaining colonies displaying a G418-resistant phenotype in the absence of a mutation at residue 135 may be due to a mutation elsewhere in *KanMX* (e.g. position 177), or a mutation elsewhere in the yeast genome (e.g. a mutation generating an amber suppressor tRNA). To this end, we repeated this experiment with an additional 10 colonies, this time amplifying and sequencing both mutated loci (position 135 and 177). Of these 10, 9 have mutations at position 135 that were previously observed. However, no other mutations in *dKanMX* were observed, including at position 177, in any of the 10 sequenced revertants. As stated above, the generation of an amber suppressor tRNA may be the cause of the remaining resistant isolate. We therefore conservatively conclude that at least 88% (43/49) of G418-resistant colonies appeared as a result of direct Ty1-mediated mutagenesis to d*KanMX*.

This conservative rate of reversion mutants present in ICE-d*KanMX* above that of g-d*KanMX* (Fig. 4a) was then compared to the expected rate of mutation based on sequencing experiments described previously. With this established rate of 0.15 point mutations per bp, each transposition of the 717 bp d*KanMX* gene will result in an average of 0.10755 mutations. Given an approximate transposition rate of 10^8 per liter of culture (based on the length of the cargo cassette), 1.0 mL of culture plated on G418-containing media is expected to contain 10,755 mutations in d*KanMX*. Neglecting indel mutations, there are 8 mutations out of the 2151 possible that will eliminate the stop codon from d*KanMX*; assuming the mutations are distributed randomly and uniformly throughout the gene, this results in approximately 40 reversion mutations per 1.0 mL of culture that will be able to grow on the G418 plate. This matches remarkably closely to the rate of colony formation observed (0.88*58 colonies per mL of culture= 51 per mL of culture), confirming the utility of this assay and the rate of mutation in ICE cargo.

Supplementary Note 3: Characterization of ura3 variants

The first-round mutant *ura3(3-5)* contained a single coding mutation ($Arg^{145}Ile$) that resides on an outer loop of the URA3p $(\beta/\alpha)_8$ barrel and which is distal to both the homodimer interface and catalytic site (Supplementary Fig. 2a). After isolation and sequencing of *ura3* mutants enriched by the first round of screening, the capacities of each *ura3* variant to convert 5-FOA to 5-fluorouracil, while maintaining activity in the uracil biosynthesis pathway, were performed by integrating each variant into a low-copy vector and transformed this expression cassette into BY4741 Δ*rrm3*. Cells containing either a mutant *ura3* or wild-type *URA3* were then plated on solid media lacking uracil and containing 5-FOA, and relative growth rate was quickly determined by comparing colony size. Strains expressing *ura3(3-5)* enabled over 3-fold increases

to colony area relative to those expressing the wild-type gene, indicating a decreased propensity of these mutants to convert 5-FOA into 5-fluorouracil while retaining their function in the uracil biosynthesis pathway (Supplementary Fig. 3b). Importantly, cells containing this mutant did not exhibit decreased growth rate relative to those containing wild-type *URA3* in uracil-deficient media *without* 5-FOA, indicating that their increased specificity came with no observable fitness tradeoff under these conditions.

ura3(3-5-2), the best-performing mutant from the second round, contained a second coding mutation ($Arg^{186}Lys$) that was likewise distal to the active site and the homodimer interface (Supplementary Fig. 1a). *ura3(3-5-2)* was also cloned into a low-copy vector and transformed into BY4741. Cells containing either *ura3(3-5)*, *ura3(3-5-2),* or wild-type *URA3* were then subjected to growth assays in liquid media lacking uracil but containing from 0 g L^{-1} 5-FOA to 1 g L^{-1} 5-FOA (which is the concentration generally used for efficient counter-selection of the *URA3* marker[19]). Maximum exponential growth rates at each condition were measured in order to compare the relative specificity of each enzyme. In all conditions, strains containing *ura3(3-5-2)* grew the fastest followed by *ura3(3-5)* and finally *URA3*. In particular, cells containing *ura3(3-5)* and *ura3(3-5-2)* attained IC_{50} values for 5-FOA 1.6- and 2.5-fold higher than strains expressing wild-type *URA3*, respectively (Fig. 4a). Again, neither mutant strain exhibited decreased growth rate relative to those containing wild-type *URA3* in uracil-deficient media without 5-FOA, confirming this increased specificity did not practically affect native activity.

Supplementary Note 4: Characterization of spt15 variants

The best-performing mutant isolated from the first round, *spt15-B6*, contains a single coding mutation ($Arg^{98}His$) near the DNA-binding domain of this protein (Supplementary Fig.

2b), suggesting a putative mode of action. *Spt15-B6-1,* the best-performing mutant from the second round of ICE contained a second coding mutation ($Gly^{192}Ser$) along with two indels in the TEF1 promoter (Fig. 4b). This coding sequence mutation, like that of *spt15-B6*, resides in the DNA-binding domain (Supplementary Fig. 2b).

After isolation and sequencing of *spt15* mutants enriched by our initial screening, viability analyses of *spt15* mutants were performed by integrating each *spt15* mutant into a low-copy vector and transforming this expression cassette into wild-type BY4741. These strains, along with controls expressing either wild-type *SPT15* or an empty vector, were grown to stationary phase and then subjected to a killing concentration of 1-butanol (3.5% by volume). After 0, 1, 2, and 3.5 hours, a small volume of each culture was plated to determine the number of viable cells remaining (see Methods). This analysis indicated that cells containing *spt15-B6* exhibited a 1.7-fold higher viability in lethal 1-butanol concentrations compared to wild-type, while cells containing *spt15-B6-1* exhibited up to 1.95-fold higher viability relative to wild-type under the same conditions (Fig. 4b).

These mutants were then further tested for any potential growth improvements in butanol-containing media. The same strains, along with controls expressing either wild-type *SPT15* or an empty vector, were grown to stationary phase and then resuspended at a low OD in media containing between 1.2% and 1.5% butanol. These were then grown in anaerobic sealed culture tubes. *Spt15-B6-1* conferred 32% and 44% increased growth over wild-type at 1.3% and 1.4% 1-butanol, respectively ($p<0.05$). As *spt15-B6-1* also contained mutations to *pTEF1*, a qRT-PCR experiment was carried out to investigate potential changes to transcription levels in order to provide insights into the observed phenotype (see "Expression Analysis" above). However, expression measurements indicated no difference between the rate of transcription

enabled by *pTEF1* and the promoter contained in *spt15-B6-1* (Supplementary Fig. 3a) under these test-tube, exponential growth conditions.

Supplementary Note 5: Development of an optimal *in vivo* mutagenesis host for screening xylose pathway variants

We performed several modifications to our optimized Ty1-containing strain in order to enable selection for growth on xylose. In particular, *GRE3*, which encodes an aldose reductase, was knocked out in order to reduce competitive xylose utilization and allow any potential improvements in xylose isomerase activity to confer a greater phenotypic advantage, thus increasing the sensitivity of our growth-based screen[20]. An additional copy of *XKS1* was also integrated into the genome to boost downstream metabolic flux. Finally, since overexpression of transaldolase (*TAL1*) has been shown to improve xylose consumption rate[21], the *TAL1* gene was expressed under the control of *pTEF1* and cloned into the $tRNA^{iMet}$ overexpression vector. The resulting plasmid was introduced into BY4741 Δ*rrm3* containing the *GRE3* knockout and *XKS1* overexpression, resulting in BY4741 Δ*r-g-x*. The two-gene xylose catabolic pathways consisting of xylose isomerase and xylulokinase were driven by a strong hybrid *pTDH3* promoter (UAS_{TEF}-UAS_{CIT}-UAS_{CLB}-P_{GPD})[22] and pathway genes were joined using ribosome-cleavable 2A sites[23]. These pathways were then inserted into the synthetic retroelement and integrated into the genome of BY4741 Δ*r-g-x*, collectively forming the parent strains for ICE of each xylose pathway.

Supplementary Note 6: Characterization of xylose pathway variants

The *I3K-66* and *I3K-20* strains contain mutants with one ($Ile^{433}Val$) and three ($Ala^{48}Ser$, $Ile^{433}Val$, $Met^{435}Ile$) amino acid substitutions, respectively. *I3K-66* also contains one silent mutation in *xyla3** (A1029G). Interestingly, A48S lies inside the $(\beta/\alpha)_8$ barrel of *xylA*, which

houses the dual Mg catalytic core and thus potentially influences the active site of this enzyme. $Ile^{433}Val$ and $Met^{435}Ile$ both lie proximal to the homodimer interface and potentially influence the stability of the XylA3*p catalytic tetramer[24] (Supplementary Fig. 2c). Enzymatic assays of the isolated mutants indicated increased V_{max} values (0.126 ± 0.008 and 0.134 ± 0.003 μmol min^{-1} mg $protein^{-1}$ for *I3K*-66 and *I3K*-20, respectively) compared to wild-type (0.118 ± 0.007 μmol min^{-1} mg $protein^{-1}$) (Supplementary Table 3). In comparing these values to prior work, it is important to note that *XKS1* was not overexpressed during the characterization of xylose consumption kinetics in[15], whereas it was in this work. However, in both works, Vmax and Km are computed using whole cell extracts, so the entire pathway will contribute to these values. Since isomerization is reversible, high xylulose concentrations due to the absence of sufficient *XKS1* may have increased the apparent Km in the Lee, *et. al.* study. However, because we overexpressed *XKS1* in this study, xylulose is readily removed as it is formed and thus does not compete with xylose for active sites in *XylA*, thus lowering the apparent Km.

After screening, the growth rates of strains containing xylose pathway variants were characterized through growth in 1mL cultures containing 20 g L^{-1} xylose (Supplementary Fig. 3d and 3e). It was observed that mutant *IK-34* displayed a 1.7-fold increase in growth rate over the control as well as a significantly shorter lag phase. For the *I3K* multi-gene cassette, *I3K-66* and *I3K-20* conferred roughly 1.3-fold improvements to growth rate (Supplementary Fig. 3d and 3e, Supplementary Table 3) concomitantly with a decrease in lag time. In order to characterize the growth curves of each mutant in more detail, optical density was measured over short (15 minute) intervals through the use of a Bioscreen C. After scaledown from 1 mL to 200 μL in this setting, mutants *IK-34, I3K-66*, and *I3K-20* displayed 20.5%, 14.0%, and 16.2% improvements to growth rate in xylose-containing media over their respective wild-type,

respectively (Fig. 4c). Mirroring results at the 1mL scale, mutant *IK-34* exhibited an 18 hour shorter lag, while mutants *I3K-66* and *I3K*-20 exhibited a 6 hour shorter lag phase. These analyses produced similar results and indicated that variants of strains containing *IK* and *I3K* exhibited significantly increased growth rates and significantly reduced lag times compared with that of their parent strains.

These mutants were isolated at the following frequency at the end of the selection experiment: IK-34: 2/52. I3K-66: 1/66, I3K-20: 1/26. To assess and calculate actual enrichment values, we first calculated the ratio of these clones we would have expected based on no selective pressure. To generate these variants, we induced mutagenesis in 4.6kb pathway for 3 days. Based on Supplementary Figure 2a, this yields ~3*10^-3 transposants per cell. Since we induced mutagenesis in 50mL of OD=1 culture, the total number of transposants generated is 50*10^7*1*3*10^-3=1.5*10^6. Given that IK-34 and I3K-66 have one mutation each while 13K-20 has three, we can compute the expected copy number of each 1-mutant and 3-mutant variants in our generated library using PEDEL[6]. This analysis shows that each 1-mutant variant is at a copy number of 37.6, while each 3-mutant variant is unique. This indicates that the expected probability of finding IK-34 and I3K-66 is 37.6/1.5*10^6=2.5*10^-5, and of finding I3K-20 is 6.7*10^-7 if no selection was occurring. Given the observed recovery frequencies of these variants, we concluded that IK-34 is enriched 1500-fold, I3K-66 610-fold, and I3K-20 57000-fold over the initial library.

We were initially surprised that none of these variants contained mutations in all of the promoter and coding sequences. As one potential explanation, we posit that in multigene pathways, it is only really necessary to improve the rate-limiting step in order for pathway performance to be increased, which may only map to one enzyme or promoter region. For long

evolutionary time courses, multiple genes and regulatory elements will each eventually undergo mutations as they become the rate-limiting step in further improvements.

Supplementary References

1. Ravikumar A, Arrieta A, Liu CC. An orthogonal DNA replication system in yeast. *Nat Chem Biol* **10**, 175-177 (2014).

2. Zhu YO, Siegal ML, Hall DW, Petrov DA. Precise estimates of mutation rate and spectrum in yeast. *Proc Natl Acad Sci USA* **111**, E2310-E2318 (2014).

3. DiCarlo JE, Conley AJ, Penttilä M, Jäntti J, Wang HH, Church GM. Yeast Oligo-Mediated Genome Engineering (YOGE). *ACS Synth Biol* **2**, 741-749 (2013).

4. DiCarlo JE, Norville JE, Mali P, Rios X, Aach J, Church GM. Genome engineering in *Saccharomyces cerevisiae* using CRISPR-Cas systems. *Nucleic Acids Res* **41**, 4336-4343 (2013).

5. Romanini DW, Peralta-Yahya P, Mondol V, Cornish VW. A Heritable Recombination System for Synthetic Darwinian Evolution in Yeast. *ACS Synth Biol* **1**, 602-609 (2012).

6. Firth AE, Patrick WM. GLUE-IT and PEDEL-AA: new programmes for analyzing protein diversity in randomized libraries. *Nucleic Acids Res* **36**, W281-W285 (2008).

7. Volles MJ, Lansbury PT, Jr. A computer program for the estimation of protein and nucleic acid sequence diversity in random point mutagenesis libraries. *Nucleic Acids Res* **33**, 3667-3677 (2005).

8. Gietz RD, Schiestl RH. High-efficiency yeast transformation using the LiAc/SS carrier DNA/PEG method. *Nat Protoc* **2**, 31-34 (2007).

9. Keefe AD, Szostak JW. Functional proteins from a random-sequence library. *Nature* **410**, 715-718 (2001).

10. Mumberg D, Muller R, Funk M. Yeast vectors for the controlled expression of heterologous proteins in different genetic backgrounds. *Gene* **156**, 119-122 (1995).

11. Karim AS, Curran KA, Alper HS. Characterization of plasmid burden and copy number in Saccharomyces cerevisiae for optimization of metabolic engineering applications. *FEMS yeast research* **13**, 10.1111/1567-1364.12016 (2013).

12. Guldener U, Heck S, Fielder T, Beinhauer J, Hegemann JH. A new efficient gene disruption cassette for repeated use in budding yeast. *Nucleic Acids Res* **24**, 2519-2524 (1996).

13. Hegemann JH, Heick SB. Delete and repeat: a comprehensive toolkit for sequential gene knockout in the budding yeast *Saccharomyces cerevisiae*. *Methods Mol Biol* **765**, 189-206 (2011).

14. Crook NC, Freeman ES, Alper HS. Re-engineering multicloning sites for function and convenience. *Nucleic Acids Res* **39**, e92 (2011).

15. Lee SM, Jellison T, Alper HS. Directed evolution of xylose isomerase for improved xylose catabolism and fermentation in the yeast Saccharomyces cerevisiae. *Appl Environ Microbiol* **78**, 5708-5716 (2012).

16. Blazeck J, Garg R, Reed B, Alper HS. Controlling promoter strength and regulation in *Saccharomyces cerevisiae* using synthetic hybrid promoters. *Biotechnol Bioeng*, (2012).

17. Jin Y-S, Ni H, Laplaza JM, Jeffries TW. Optimal Growth and Ethanol Production from Xylose by Recombinant Saccharomyces cerevisiae Require Moderate d-Xylulokinase Activity. *Appl Environ Microbiol* **69**, 495-503 (2003).

18. Kim C, Mobashery S. Phosphoryl transfer by aminoglycoside 3'-phosphotransferases and manifestation of antibiotic resistance. *Bioorg Chem* **33**, 149-158 (2005).

19. Boeke JD, Trueheart J, Natsoulis G, Fink GR. 5-Fluoroorotic acid as a selective agent in yeast molecular genetics. *Methods Enzymol* **154**, 164-175 (1987).

20. Träff KL, Otero Cordero RR, van Zyl WH, Hahn-Hägerdal B. Deletion of the GRE3 Aldose Reductase Gene and Its Influence on Xylose Metabolism in Recombinant Strains of Saccharomyces cerevisiae Expressing thexylA and XKS1 Genes. *Appl Environ Microbiol* **67**, 5668-5674 (2001).

21. Jin YS, Ni H, Laplaza JM, Jeffries TW. Optimal growth and ethanol production from xylose by recombinant Saccharomyces cerevisiae require moderate D-xylulokinase activity. *Appl Environ Microbiol* **69**, 495-503 (2003).

22. Blazeck J, Garg R, Reed B, Alper HS. Controlling promoter strength and regulation in Saccharomyces cerevisiae using synthetic hybrid promoters. *Biotechnol Bioeng* **109**, 2884-2895 (2012).

23. Beekwilder J*, et al.* Polycistronic expression of a beta-carotene biosynthetic pathway in Saccharomyces cerevisiae coupled to beta-ionone production. *J Biotechnol*, (2014).

24. Lavie A, Allen KN, Petsko GA, Ringe D. X-ray crystallographic structures of D-xylose isomerase-substrate complexes position the substrate and provide evidence for metal movement during catalysis. *Biochemistry* **33**, 5469-5480 (1994).
